# A realist impact evaluation of a tool to strengthen equity in local government policy-making

**DOI:** 10.1186/s12939-024-02266-5

**Published:** 2024-09-10

**Authors:** Sally Schultz, Felicity Beissmann, Christina Zorbas, Serene Yoong, Anna Peeters, Kathryn Backholer

**Affiliations:** 1 Global Centre for Preventive Health and Nutrition, School of Health and Social Development, Institute for Health Transformation, Deakin University, 1 Gheringhap Street, Geelong, VIC Australia; 2 City of Greater Bendigo, Galkangu Bendigo, VIC 189-229 Lyttleton Terrace Australia

**Keywords:** Health equity tool, Health equity impact assessment, Local government, Intersectionality

## Abstract

**Background:**

Local governments have a critical role to play in addressing health inequities. Health equity impact assessments are recommended to help governments apply an equity lens to the development and implementation of policies and programs. Despite evidence of equity-positive benefits of such tools, adoption remains limited, prompting calls for evaluations to assess their impact and identify factors that will promote uptake across various contexts.

**Methods:**

We conducted a mixed method study to evaluate the impact of an equity impact assessment (EIA) tool and process on policies and organisational capacity in a local government in Victoria, Australia, and identify factors that influenced this impact. We analysed 33 documents related to 18 EIAs, and conducted surveys (*n* = 40) and in-depth interviews (*n* = 17) with staff involved in EIAs.

**Results:**

Almost all (17 of 18) EIAs resulted in equity-positive changes to policies and programs, most frequently addressing individual-level factors, such as making community communications and consultations more accessible to under-represented or under-served groups. Structural-level recommendations from one EIA, such as increasing diversity in decision-making panels, were found to impact both the current policy and a broad range of future, related projects and services. Improvements in equity-centric organisational culture and capacity (including staff awareness, skills and confidence) and increased engagement with under-represented communities were also reported. Factors perceived to influence the impact of EIA’s related to organisational commitment and capacity to prioritise equity, process-level factors related to the type and timing of EIAs, and extent of implementation support.

**Conclusion:**

Our study supports wider uptake of health equity impact assessments in local government policies and programs. Legislation, leadership and resources from upper-tiers of government can help increase the adoption of equity tools to reduce disparities in population health.

**Supplementary Information:**

The online version contains supplementary material available at 10.1186/s12939-024-02266-5.

## Background

The existence of unjust health inequities is well established and globally acknowledged as a critical human rights issue [1–3]. Addressing the social determinants of health, such as income, education, housing, discrimination, social exclusion and access to health services, is fundamental for reducing health inequities [4]. Differences in the way individuals experience the social determinants of health stems from the unequal distribution of power and resources within society. Government policies, plans, programs and services (herein referred to as policies) have a substantial influence on the distribution of societal power and resources [5, 6]. For example, an Australian study that examined data from 158 qualitative interviews found structures of capitalism, neoliberalism, colonisation, sexism and racism were embedded in Australia’s macroeconomics, employment, social protection, welfare, health, infrastructure and land use planning policies, resulting in policies that favour some groups while disadvantaging others [7]. In Australia, groups who disproportionately experience health inequities include, but are not limited to, those experiencing socioeconomic disadvantage, Aboriginal and Torres Strait Islander peoples, culturally and linguistically diverse communities, people living in remote areas and women [8–10].

All levels of government have a responsibility to ensure that policies actively work to rebalance power and resources and avoid perpetuating health inequities. Local government (also known as a municipality, City or council) have a specific responsibility for community health, as well as infrastructure and services related to the social determinants of health, such as local roads, parks and recreation spaces, and are an important context for addressing inequities [5, 11]. Echoing recommendations by health agencies globally, our recent study found that local government stakeholders identified equity tools as one mechanism to strengthen health equity in policy-making, and tools should be applied across a broad range of local government policy actions [12–16].

Health equity impact assessments are one type of equity tool available to governments. Health equity impact assessments are a document and/or process comprising steps and questions that aim to help users identify (i) how a policy may differentially impact groups in the population, (ii) barriers to equitable access and benefit, and (iii) actionable recommendations to promote fairer outcomes [17]. Examples include equity-focused health impact assessments (EF-HIAs) which incorporate equity-focused questions into the well-established Health Impact Assessment (HIA) tool,) the UK’s Health Equity Audit (HEA) and Health Inequality Impact Assessment (HIIA), New Zealand’s Health Equity Assessment Tool (HEAT), the World Health Organization’s Urban Health Equity Assessment and Response Tool (Urban HEART) and the Intersectionality-Based Policy Analysis (IBPA) framework [17–23].

Studies evaluating health equity impact assessments in the international literature are limited. In Australia and New Zealand, retrospective case-study evaluations of EF-HIA’s and HEAT reported variable impacts on the development of health policies. When positive equity impacts were reported, it included the adoption of policies that removed financial and transport barriers, improved engagement with under-represented groups to inform policy development and reprioritisation of resources targeted at health equity strategies [24–26]. However, for some cases, it was less clear if changes made to policy development processes were informed by the health equity impact assessment itself, or would have occurred during regular program development processes. These case studies, and others from Canada, also found a range of organisational-level impacts from health equity impact assessments, including increased dialogue and understanding of equity, engagement with a broader stakeholder group in planning processes, improved connections with community, and increased use of equity-related data and information to inform decisions [24–28]. Despite the existence of equity impact assessment tools and some promising equity benefits reported in health policy and planning contexts, the adoption of such tools across all levels of government, including local government, remains limited. This has prompted calls from public health researchers for further studies to assess the impact of health equity impact assessments in different contexts and identify factors that will promote their uptake and likely effectiveness in policy decision-making [29].

There is also a growing interest in the concept of intersectionality, which acknowledges that people’s lives are shaped by their identities (e.g. gender, ethnicity, ability, age) and social factors (e.g. socioeconomic status, migration status, geographic location, housing status, family violence), which combine to create intersecting forms of privilege and oppression depending on a person’s power within society’s structures and systems [30]. Equity impact assessments underpinned by an intersectional lens can help local government policy-makers understand how historic and current practices contribute to power imbalances and discrimination that contribute to health inequities.

In this study, we aimed to (i) evaluate the impact of an intersectional equity impact assessment (EIA) tool on policies, programs, services and organisational capacity in a Victorian local government and (ii) identify the factors that influenced the impact of the EIA tool and process, as perceived by local government employees.

## Materials and methods

### Study setting

The City of Greater Bendigo (referred to herein as “the City”) is a large regional local government authority located in Victoria, Australia. In 2021, the City introduced an Equity Impact Assessment (EIA) tool and process (Supplementary material). The EIA tool was informed by the Public Sector’s Gender Impact Assessment (GIA) Toolkit and Template, developed by the State’s Commission for Gender Equality in the Public Sector and designed to support the implementation of Victoria’s Gender Equality Act 2020 (The Act). The Act requires all public sector organisations, including local governments, to conduct a GIA on policies, programs and services that have a direct and significant public impact [31, 32]. The City’s EIAs follow a similar format to other health equity impact assessment tools. EIAs are underpinned by an intersectional lens that explores how factors related to ‘people’, ‘place’ and ‘experiences’ intersect to contribute to gender inequality and other forms of inequity. Like GIAs, EIAs at the City are intended to be applied across all policies, programs and services with a direct and significant public impact, including those that directly impact health (i.e. policies that relate to community health and wellbeing) and other policy areas (i.e. policies related to directorates responsible for infrastructure, assets and strategy).

EIAs are conducted by City staff (EIA Leads) who are leading the policy, program or service development or review process. A number of strategies support the implementation of EIAs. EIA Champions are staff members who have received specialised training in applying an intersectional gender lens on policies, and provide mentoring support to EIA Leads. The EIA Lead invites staff members and other relevant stakeholders to an EIA Workshop with a priority on recruiting people with diverse equity lenses. Workshops begin with an introduction to gender equality and intersectionality, followed by a discussion guided by the EIA toolkit to identify a key focus area for the EIA Lead to explore further. The EIA Lead is then responsible for conducting further research related to the focus area and develop recommendation/s. A centralised role provides specialist equity support and oversees the process to ensure EIAs meet their legislative obligations.

### Study design

We conducted a realist impact evaluation of the City’s EIAs. Realist evaluation is a theory-based approach that begins by clarifying the program theory, including relationships between the context and mechanisms of a program, and the expected outcomes [33]. It assumes that nothing works everywhere for everyone and is interested in how, why and for whom an initiative works. Realist evaluation is often used for evaluating new initiatives or programs, particularly when the intention is to scale up the intervention or implement it in other contexts [29, 34, 35]. Our impact evaluation constituted a mixed method study design using a combination of document analyses, a quantitative and qualitative survey and semi-structured interviews. By examining and triangulating information collected through these different methods, we were able to corroborate data, leading to more robust and comprehensive findings [36].

### Conceptual frameworks

Our study was guided by two frameworks. Firstly, the ‘UN Women’s Intersectionality Framework for Action’ was developed to help policymakers think holistically about how they support empowerment of those experiencing intersectional discrimination. It describes four constructs that act upon individual-level factors: (i) building agency, commitment, knowledge and skills; (ii) improving access to and control over resources and opportunities, as well as addressing system and structural-level factors; (iii) shifting social norms, attitudes and exclusionary practices, and (iv) developing more equitable laws and policies, resource allocation and accountability mechanisms [30]. This analytical framework was used to categorise the recommendations produced by the City’s EIA process based on how the recommendations attempted to address intersectional discrimination. Secondly, the ‘Revised framework for evaluating the impact and effectiveness of equity focused health impact assessments’ encompasses a range of contextual, process and potential impact factors to guide evaluations of equity-oriented impact assessments [24]. This framework guided survey and interview instruments to explore potential proximal and distal impacts of the EIA process, as well as contextual factors that may influence effectiveness of the EIA process.

### Sample, recruitment and data collection

The City provided documents related to all EIAs completed between October 2021 and February 2024 for analysis. For each EIA, we reviewed all documents provided, including completed EIA documents, EIA workshop presentations, policy documents that the EIA was conducted on, and progress reports related to EIA outcomes. To address research question 1, SS extracted the following data, which was cross-checked by FB: name of policy, directorate responsible for the policy (Healthy Communities and Environments, Presentation and Assets, Strategy and Growth, Corporate Performance), if the policy was new or under review, whether recommendations were approved and implemented (fully approved, partially approved, not approved) and the extent recommendations have been implemented (full, partial, in planning).

We invited all employees who had been involved in an EIA (fully completed EIAs and those still in progress) to participate in an online survey and/or an interview to explore perceptions about the impact of EIAs on policies, programs and services, and the factors that influenced these impacts (research questions 1 and 2). An authorised officer within the City sent all participants an email inviting them to take part in the survey and interview.

Our research team developed the online survey with input from the City. We administered the survey through the Qualtrics platform, incorporating both quantitative and open-ended qualitative questions, which took approximately 10 min to complete. Participants self-reported data on the number of EIAs they had been involved in (“1”, “2–3”, “4–5”, “6 + ”), type of policy the EIAs were conducted on (policy, program, service, plan/strategy/framework) and their role in the EIA process (“EIA Lead”, “EIA Champion”, “EIA workshop participant”, “EIA recommendation authorising manager”). The overall perceived impact of the EIA process was measured by asking participants whether the EIA process identified and addressed barriers to equity, and if it resulted in a more equitable policy, using a 5-point Likert scale, including “strongly agree”, “agree”, “neither agree/disagree or unsure”, “disagree” and “strongly disagree”. The perceived impact of the EIA process on organisational capacity was measured in terms of its value to existing policy processes and its enhancement of equity considerations in workforce culture, awareness, knowledge and confidence, using a 5-point Likert scale including “much better”, “somewhat better”, “about the same”, “somewhat worse”, “much worse” and “not applicable/unsure”. The survey was open from October 2023 – January 2024. All responses were anonymous.

Staff members who expressed interest in participating in a semi-structured interview were interviewed either face-to-face or via Microsoft Teams between November 2023 – January 2024. Participants were asked questions related to their perceptions of the impact that the EIA process had on policies and organisational capacity, as well as the perceived barriers and enablers of effective EIAs. Interviews were recorded, with participant consent, and data was transcribed verbatim. Participants were given the opportunity to review and edit a copy of the transcript. All data was de-identified.

### Data analysis

We used a convergence model of triangulation to analyse mixed methods data concurrently, with results compared, contrasted and merged for interpretation.

We descriptively summarised the frequency of characteristics related to: directorate responsible for the policy, if policy was new or under review, whether recommendations were approved and extent of implementation. The first author (SS) categorised recommendations produced by the EIAs according to the four constructs of the UN Women’s Intersectionality Framework for Action, which was blind cross-checked by another author (CZ), resolving discrepancies through discussion.

We analysed the quantitative survey data descriptively by calculating means, standard deviations and frequencies, as appropriate. Given the sample size (*n* = 40) and to aid interpretation of results, we grouped responses to Likert scale variables into three categories: Agreement-based questions were collapsed into three responses, including Agree (strongly agree and somewhat agree); Disagree (strongly disagree and somewhat disagree) and Neither agree or disagree / Unsure. Variables related to change in equity-related organisational capacity were grouped into four categories: Better (much better and somewhat better); Worse (much worse and somewhat worse); About the same; Unsure / Not applicable.

We managed data from interview transcripts and open-ended responses in the survey using NVivo 14 (IQR International software) and conducted inductive analyses using Braun and Clarke’s six steps of reflexive thematical analysis [37]. In step one, the lead author (SS) read interview transcripts, reflecting and taking notes. SS re-read the interview transcripts and generated initial codes (step 2) guided by the research questions and conceptual frameworks. In step three, SS constructed initial themes, before meeting with the research team to review and workshop key themes (step 4). Themes were then refined further for clarity and to ensure they answered the research questions (step 5), and were finally described in the form of a model, supported by text and illustrative quotes (step 6).

### Researcher reflexivity

This study is an external evaluation and as such the majority of the research team is independent of the City. The second author (FB) led the development and implementation of the EIA tool at the City, providing valuable and nuanced understanding of the tool and context to inform the study design and interpretation of results, but did not participate in data collection or preliminary analysis to maintain impartiality. All authors have extensive expertise in policy research and/or practice for enhancing health equity. The lead author (SS) practiced reflexivity throughout data collection and interpretation, documenting insights after each interview. These insights informed discussions with the broader research team, where results were interpreted with explicit regard to intersectionality theory and local government policy-making structures.

## Results

### Sample characteristics

We analysed 33 documents related to 18 EIAs. The majority (78%) of EIAs related to new policies, with the remaining focused on policies that were under review. EIAs included in the analysis related to policies from across the City, with the majority (55%) from the Healthy Communities and Environments directorate.

Forty employees participated in the survey (response rate of 68%). Participants held various roles in the EIA process, including workshop participant (44%), EIA Lead (27%), EIA Champion (15%) and ‘other’ roles including authorising manager and observer (14%). The majority had been involved in up to three EIAs (84%).

Seventeen staff members participated in one-on-one, semi-structured interviews (average duration 55 min). Participants held various positions within the City, including director/manager/co-ordinator (*n* = 5) and officer (*n* = 12) roles, representing all directorates, with the largest participation from the community health and wellbeing directorate (65%). All roles involved in the EIA process were represented in the sample (i.e., EIA Lead, EIA Champion, EIA workshop participant and authorising managers).

### Impact of EIA tool and process on policies

Document analysis of completed EIAs indicated that 17 of the 18 EIAs recommended that action be taken to make the policy, program or service more equitable. All were approved by the authorising manager, nine had been fully implemented, one partially implemented and seven were in planning. The EIA that recommended ‘no changes’ be made related to an internal operational policy, which would not typically require an EIA (as it did not meet the criteria of ‘direct and significant public impact’). The decision to conduct the EIA was made by a senior manager who wanted the EIA to explore potential gender impacts of the current policy. Table 1 provides a summary of EIAs reviewed as part of the document analysis.

**Table 1:** Appraisal of equity impact assessment recommendations against the un women’s intersectionality framework for action

Policy details			EIA details		Type of intersectional action			
					**Individual, family and community – level**		**Organisation, society, structural—level**	
**Name of policy**	**Type of directorate or department leading the EIA**	**New policy development or up for review**	**Date EIA was completed**	**Implementation status of recommendations** (Full, partial, in-planning, no implementation plan)	**Agency,** **commitment, knowledge, skills**	**Access to and control over resources and opportunities**	**Social norms, attitudes, exclusionary practices**	**Laws, policies, programmes, resource allocation accountability mechanisms**
Community Grants Program Policy	Health and community	For review	Aug 2021	Full	X	X		
Domestic Animal Management Plan	Health and community	For review	Oct 2021	Full	X			
Leases and Licences Policy	Assets and infrastructure	New	Oct 2021	Full				X
Capital Investment Framework	Assets and infrastructure	New	Mar 2022	Partial			X	
Fleet Policy	Assets and infrastructure	For review	Apr 2022	N/A	No actions recommended			
Public Art Policy	Strategy	New	Apr 2022	Full		X	X	X
Healthy Facilities Policy	Health and community	New	May 2022	Full	X			
Walking and Cycling Infrastructure Plan	Health and community	New	Jul 2022	Full	X			X
Heathcote Civic Precinct	Assets and infrastructure	New	Aug 2022	In-planning		X		
Maiden Gully Site and Infrastructure Plan	Health and community	New	Aug 2022	Full			X	
Graffiti Management Policy	Assets and infrastructure	New	Oct 2022	Full	X			
Kangaroo Flat Skate Park (GIA Pilot)	Health and community	New	Oct 2022	In-planning	X		X	
Positive Ageing Action Plan	Health and community	New	May 2023	Full	X		X	
Financial Hardship Guidelines and Revenue and Debt Collection Policy	Corporate	For review	Oct 2023	In-planning	X			
Managed Growth Strategy	Strategy	New	Jan 2024	In-planning	X			
Reducing Harm from Tobacco Action Plan	Health and community	New	Jan 2024	In-planning	X			
Fair Access policy	Health and community	New	Feb 2024	In-planning			X	
Quarry Hill Ken Wust Pavilion redesign	Health and community	New	Feb 2024	In-planning		X		

**Fig. 1:**
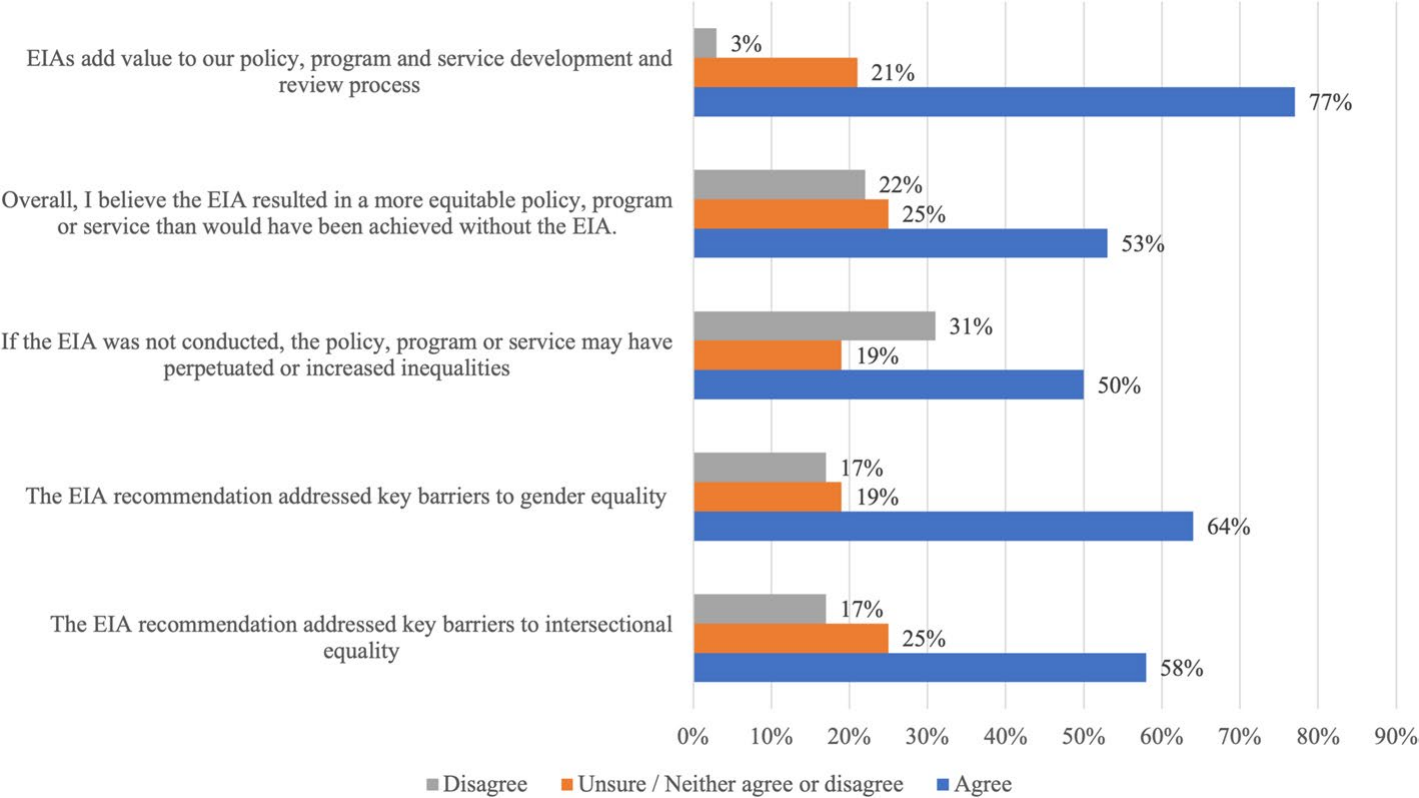
Impact of EIAs on policies, programs and services as perceived by local government stakeholders (*n* = 40)

The EIA tool and process commonly produced recommendations that acted on individual-level factors (Table 1). In particular, recommendations that aimed at improving an individual’s ‘agency, commitment, knowledge and skills’ were most frequently observed. These included making information and consultations more accessible by catering to different levels of English language literacy, digital literacy, cultural background, geographic location and internet access, as well as developing resources, training and other support for marginalised or under-represented groups. Recommendations that aimed to improve ‘access and control of resources and opportunities’ included updating the community grant application form and process to identify who was applying and who was benefiting from the grant, in order to prioritise grants from and for under-represented or marginalised groups. Several survey and interview participants also observed that often EIA recommendations targeted individual-level factors, despite a range of structural barriers to equity being identified in EIA Workshops. Some interviewees described this as “*picking the low-hanging fruit",* where recommendations are chosen because they are easier to implement, despite their potentially lower impact on inequities. This may relate to survey results that found only half of participants indicated that EIAs produced more equitable policies, despite the majority of participants (77%) indicating EIAs added value to policy development and review processes. (Fig. 1).

Where recommendations acted at the organisation/society/systems levels, some were aimed at addressing ‘social norms and exclusionary practices’. These included updating terms of reference for panels or committees to promote diversity in representation, and developing processes to better engage under-represented groups in community consultations. Recommendations related to ‘policies, laws, resource allocations and accountability mechanisms’ included the development and incorporation of equity criteria into the overall criteria for an infrastructure plan that helped prioritise resource allocation to lower-socioeconomic, remote areas. We found that both individual and structural-level recommendations had potential for broader impacts across other policies. For example, several participants described how an EIA that recommended gender neutral, culturally-appropriate, dementia-friendly toilets on a discrete building project led to discussion to develop a broader equity-centric toilet strategy that could be used to inform all future building projects. Likewise, policy changes to prioritise under-represented groups flowed down to decision-making in related programs and services.

“*I can say hand on heart, it (the EIA on a building refurbishment plan) has changed outcomes at a programmatic level, at a project level and at a service delivery level*”

– Interview participant

Several interview participants expressed frustration that while the EIA process identified a range of opportunities to address inequities, the final recommendations only included one or two actions. Given EIAs are only conducted when policies are developed or reviewed (often every 3–4 years), it was suggested that EIAs should endeavour to recommend multiple actions, which could be implemented over time if necessary.

Whilst the tool was underpinned by an intersectionality lens, participants discussed how EIAs were more likely to examine gender and other forms of discrimination in silos, rather than taking an intersectional approach. Participants suggested this siloed approach was partly due to lack of current equity-competency across the organisation, with many staff hearing about intersectionality for the first time in their first EIA workshop.

*“…it is tricky to keep all the different lenses front of mind during the workshop. It's a lot to cover and so we start to talk about experiences/needs/issues in a siloed way because there's so much to unpack in a short session”*

– Survey participant

Two notable exceptions related to public space infrastructure plans where projects initially looked at the needs of women and/or gender diverse people, then explicitly considered those who were also from low socioeconomic areas, culturally diverse and/or living with a disability.

### Impact of EIAs on organisational capacity

**Fig. 2:**
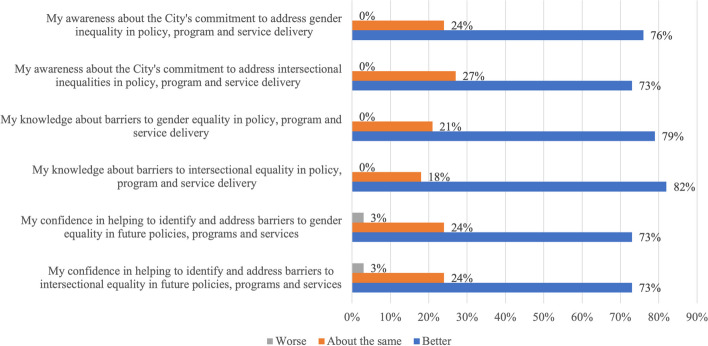
Impact of equity impact assessments on employee awareness, knowledge and confidence, as perceived by local government stakeholders (*n* = 40)

At an organisation-level, the majority of survey participants agreed that EIAs helped build an equity-focused culture (85%) and increased knowledge and skills about equity across the City (88%). Approximately three-quarters of participants reported that being involved in the EIA process improved their awareness, knowledge and confidence in addressing inequities in policies (Fig. 2). In contrast to training-only approaches to building equity capacity, interview participants described how EIAs provided the opportunity to ‘learn by doing’, noting that EIAs provided a unique opportunity for staff to learn about equity and intersectionality and challenge their own unconscious biases, in the context of their policy area.

*“I love seeing the 'a-ha' moments for participants who engage in an EIA. I think the most powerful learning for many people is through their involvement in the EIA process. I realise the perception that these are 'additional work' is present - but the benefits over time, outweigh this resistance exponentially!”*

*– Survey participant*

Notwithstanding, some staff (18–27% of survey participants) reported that the EIA process had no impact on their awareness, knowledge and confidence in addressing inequities. Interviews revealed potential reasons for this including some staff may have been resistant to the EIA process as they did not agree that an EIA was required, while others may have perceived that barriers and recommendations were “already on the radar”. Other staff believed that, even in the latter circumstance, EIAs still provided a benefit.

“*I was already aware (of the barriers) and was already planning to do it (the recommendation), but it is now better informed. We're better informed to be able to do that well… when we are implementing that action, I feel more confident we'll do a better job than we would have before the EIA*.”

– Interview participant.

Survey data revealed that, on average, participants rated the extent to which the EIA process added too much to their existing workloads as 3.21 (SD = 1.07, min = 1 (strongly disagree), max = 5 (strongly agree)). During interviews, participants indicated that leading an EIA was a lot of work, although subsequent EIAs required less time. Participants highlighted the value of the EIA process in fostering cross-departmental collaboration and engagement with those that represent equity groups, such as the multicultural officer, disability and inclusion committee and relevant external stakeholders.

### Factors that influenced the impact EIAs had on policies and organisational capacity

**Fig. 3:**
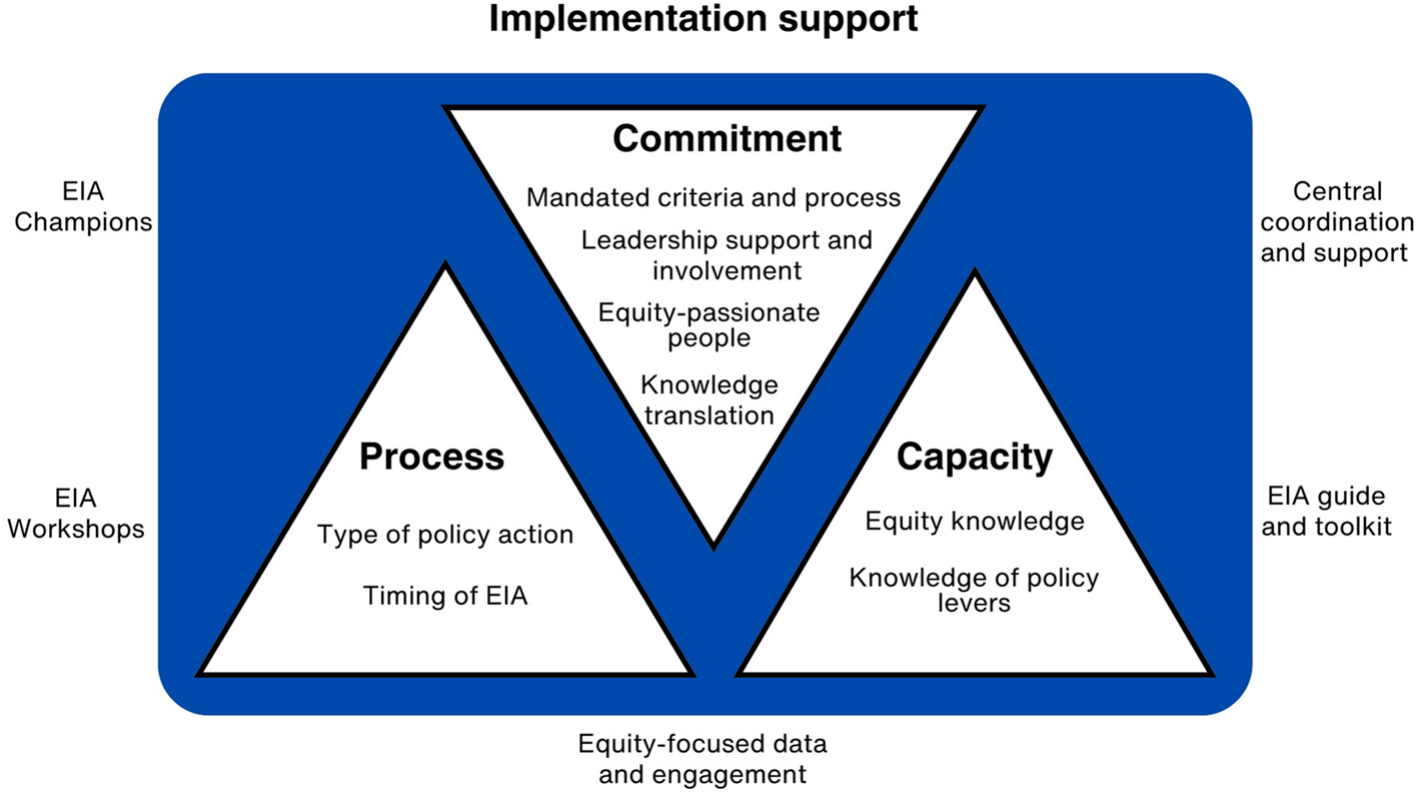
Factors perceived to influence the impact of Equity Impact Assessments (EIAs) on policies and organisational capacity

Four main themes described the key factors perceived to influence the impact of EIAs: (i) organisational commitment to conduct well-considered EIAs and implement meaningful equity-driven recommendations; (ii) organisational capacity for equity-driven policies; (iii) process-level factors related to the type and timing of EIAs and iv) implementation support, perceived to strengthen the first three themes (Fig. 3).

#### Organisational commitment to conduct well-considered EIAs and implement meaningful equity-driven recommendations

The first theme centred around the organisational commitment to conduct well-considered EIAs across the whole organisation and an explicit intent to identify and implement recommendations that will have a meaningful equity impact.

##### Mandated criteria and process

Both external and internal mandates were perceived to enable the uptake of the City’s EIA tool and process. As the City’s EIA tool is an adaptation of state-legislated Gender Impact Assessments (GIAs), there is clear criteria for determining when EIAs need to be conducted (i.e. on all policies, programs and services that have a significant and direct public impact). This external legislation was perceived as crucial for addressing the political vulnerabilities inherent in the organisation’s uptake of EIAs, particularly in policy areas not directly related to health. Participants also discussed the importance of two internal governance mechanisms. Firstly, an "Is an EIA required?" survey is completed and submitted to a centralised officer to ensure EIAs are conducted as required by legislation and the City’s internal policy. Additionally, the EIA process is integrated into approval systems for policies that require executive management endorsement, which meant that the City’s executive request evidence of an EIA (or justification for why it was not required) prior to approval.

##### Leadership support and involvement

Councillors and senior leaders at the City were perceived to possess strong, authentic equity values, and explicitly support EIAs and equity-driven policy-action. This was exemplified by actions such as the executive management team setting Key Performance Indicators (KPIs) for themselves to participate in EIAs, managers incorporating EIA updates in team meetings, and team leaders supporting EIA Leads with adequate time and resources to conduct well-considered EIAs. To further reinforce this leadership support for EIAs, some participants suggested introducing EIA participation KPIs for all levels of management. Furthermore, more explicit executive-level support and resources to implement recommendations that address both structural and individual-level barriers was considered important to generate EIAs that produce more equitable policy impacts.

*“What we do and don’t fund represents what we do and don't value, that's just the reality.”*

- Interview participant

##### Equity-passionate people

Participants highlighted the important role passionate staff, who embody strong equity and community values, played in supporting and advocating for EIAs to be done and done well. When these equity-passionate people were in an EIA Lead role, they were perceived to commit more time and effort to the process, especially in research and stakeholder engagement activities. They often volunteered to be EIA Champions or EIA Workshop participants, and if they were in a manager role, they were perceived to be more likely to support well-considered EIAs. Interview participants residing in the directorate responsible for community health and wellbeing reported that they generally felt well supported within their team, while some participants from other directorates discussed feeling isolated due to a lack of knowledge or support for equity and EIAs within their team.

##### Knowledge transfer and translation

Often interview participants (unless they were EIA Leads or Champions) reported being unaware of the final recommendations or potential outcomes of EIAs. It was suggested that centralised collation, reporting and celebration of learnings, outcomes and impacts produced by EIAs would mean those that had been involved in an EIA would be more likely to see value in the process, interest and commitment from those that had not yet been involved in an EIA may increase, and efficiencies could be gained by translating learnings between EIAs.

*“I think we should have a register that talks about all the epiphanies that we've had as a result of these conversations …I would love to see that being captured so we can look at getting buy-in from those late adopters, but also celebrate.”*

-Interview participant

#### Organisational capacity for equity-driven policies

The second theme centred around staff’s ability to identify relevant equity considerations and develop recommendations that are likely to strengthen equity in policies.

##### Equity knowledge

Participants discussed the benefit of staff possessing a foundational understanding of equity and intersectionality before engaging in their first EIA. Those with backgrounds in health, social work, or community development, those with a lived experience of inequities and those with prior EIA experience were perceived to add significant value to the EIA process. Conversely, for many participants, their initial exposure to equity-related concepts occurred during a brief introduction in the EIA workshops. Consequently, separate foundational equity training prior to an employee’s first EIA was commonly recommended.

*“In some ways I think we've run before we could walk... the process is more sophisticated than most people's knowledge and skill levels. We need to keep building people's understanding of key concepts, why this process is important and the benefits it can bring.”*

-Interview participant

##### Knowledge of policy levers

While participants generally agreed that addressing structural barriers was key for EIAs to have a meaningful impact, diverse perspectives on the extent of local government power to act on such barriers was observed. Some participants perceived that structural-level actions, such as social housing initiatives, were largely beyond the scope of local government. Others, however, perceived numerous avenues available to local governments, including advocating to higher government levels and non-government organizations, redistributing local government resources and opportunities, demonstrating leadership in inclusivity and equity-driven policies and practices. While a few participants perceived that existing regulations and codes (such as ‘universal design principles’, access and inclusion principles and building codes), already provided an equity lens for some local government policies, the majority discussed how EIAs complemented and advanced these regulatory and voluntary tools by providing an important person-centered, intersectional lens.

#### Fit of EIA with policy and process characteristics

The extent to which EIAs fit with the type of policies and timing of EIAs within the policy development or review process was perceived to have considerable influence on the impact of EIAs.

##### Type of policy

EIAs were sometimes perceived to be more effective for identifying barriers to health equity related to a specific program or projects, such as community programs and infrastructure projects, and less so for high-level strategies, such as a 30-year housing and infrastructure growth strategy. While acknowledging the advantages of a mandated equity tool, some participants advocated for greater autonomy and flexibility in how, and when, the EIA tool and process is applied and suggested that a different type of equity tool or approach may be more suitable for some policy types.

*“I really think it works really well when you've got like a master plan for a park or like a discrete project, you can really tease it out. But for such a high level document, it was really quite a struggle to come up with something that we might focus on… so I suppose in a way, because it was so high level, I'm not sure how much value it added.”*

*- Interview participant*

##### Timing of EIAs

Participants generally advocated for conducting EIAs as early as possible in the policy process, recognizing that some policies were too advanced for structural recommendations to be made, potentially resulting in individual-focused recommendations. Conversely, when policies and projects were not yet scoped, the absence of context and parameters were perceived to make it challenging to select a focus area or develop recommendations, potentially resulting in suboptimal outcomes. In these instances, flexibility in the timing of EIAs to optimize outcomes was suggested.

#### Implementation support

This cross-cutting theme relates to the critical role implementation support played in optimising the impact that the EIA process had on policy-action and on organisational capacity. Firstly, the EIA guide and toolkit that was developed specifically for the City was highly valued, and was perceived to provide clear steps and guiding questions to help staff through the process. Some participants recommended that the language could be further simplified to ensure it is accessible to everyone regardless of previous equity knowledge.

EIA Workshops were also valued for their ability to facilitate diverse input into the EIA process, especially when they could tap into the expertise of internal or external specialists (e.g. cultural diversity officer, disability and inclusion committee). Participants acknowledged the tension between EIA Workshops serving as a platform to gain valuable input from diverse, equity “specialists” and as a platform for building equity-related competency in staff. This was perceived to have important implications for who was invited to workshops, with participants generally indicating a balance between equity “specialists” and those with less knowledge and experience would be beneficial.

EIA Champions were perceived to be integral to supporting staff, especially early in their EIA journey. Some staff acknowledged further benefit when EIA Champions were from the same directorate or a similar team as EIA Lead and Workshop participants.

*“I think having those EIA Champions across the org from different directorates is really positive because it is that trickle thing. And people are always going to respond to people in their own particular area… If someone in finance can see that someone else in finance is asking equity type questions then I just think it just makes it more relevant.”*

-Interview participant

Participants suggested that for EIAs to have greater impact, EIA Leads could benefit from more assistance in accessing and utilising equity-centric data and information. Furthermore, ensuring a strong intersectional equity lens is applied to standard community engagement practices so under-represented voices are already present in community consultation data prior to the commencement of an EIA was suggested as one way to avoid EIAs producing lower-impact recommendations, such as a recommendation to conduct more equitable community consultation.

## Discussion

From our realist, mixed method evaluation, we found that conducting EIAs in a local government context led to tangible changes to policies, such as improving accessibility in communications and community engagements and promoting diverse representation in decision-making committees. EIAs also had positive impacts on building organisational capacity by increasing staff awareness, knowledge and confidence to address social inequities. Whilst the number and nature of recommendations from the EIA process varied for each policy, the majority were fully approved and either fully implemented or in planning stages. Although EIAs were generally perceived to add value to policy development and review processes, there was less consensus on whether they made policies more equitable. Factors influencing the impact of the EIA tool and process related to four key themes: (i) organisational commitment to conduct well-considered EIAs and implement meaningful equity-driven recommendations; (ii) organisational capacity for equity-driven policies; and (iii) process-level factors related to the type and timing of EIAs and iv) implementation support perceived to strengthen the first three themes.

### Impact of EIAs

Our findings that EIAs resulted in equity-driven changes in policies aligns with previous research. Two Australian studies examining the impact of EF-HIAs on state-level health sector plans revealed that these tools helped policy-makers develop equity-driven modifications to these plans [24, 25]. These case studies reported some disagreement regarding whether observed impacts could be attributed to the equity assessment, or if impacts would have occurred as a result of usual policy processes. Our study adds clarity to this, finding that even in instances where recommendations were already ‘on the radar’, EIAs could reinforce and/or improve these existing recommendations. A recent evaluation of HEAT in a health service in New Zealand similarly revealed that the equity assessment process encouraged participants to reinterpret the health issues from an equity perspective and improved equity-focused planning and decision-making [26]. Furthermore, both our study and the HEAT evaluation showed that recommendations that acted to address inequities in one area had potential to permeate through to programs or projects in other areas, thereby extending their impact. Our finding that EIAs were not considered suitable for all policies reinforces previous research that raised the importance of equity tools being fit for purpose [38]. Further research could explore different types of tools for different policy types to ensure equity considerations are effective in all policy development and review processes.

We observed positive perceived impacts of the EIA process on staff, including increased awareness and knowledge about equity, increased confidence in applying equity concepts to policy processes and improved collaborations across the organisation to build equity capacity. This is consistent with previous health equity assessment research from Australia, New Zealand and Canada [24–28]. Together, these findings suggest policy-makers should not only recognise the tangible changes to policies brought about by the EIA process, but also the value of equity tools in building equity-centric capacity and culture. Such efforts are likely to strengthen and sustain equitable policy action in the long term. While our study showed positive equity impacts of an equity impact assessment tool and process in a local government with strong existing leadership commitment to equity, further research is needed in local government contexts with different levels of equity-centric organisational leadership and culture.

### Enablers of effective EIAs

The international literature advocates for greater government leadership to promote the utilization of equity tools [26, 27, 38–40]. We observed a high value placed on The Act, which requires all Victorian public sector organisations to conduct gender impact assessments on policies, programs and services with a significant and direct community impact. This provided clear, mandated criteria for the City’s adapted EIA tool. Our findings suggest that other jurisdictions could consider similar legislation to promote the use of equity tools in local governments.

There is strong evidence to suggest legislation is only effective when coupled with robust leadership and implementation support [26, 41]. In our study, setting key performance indicators for leaders to participate in the EIA process, commitment to invest in implementation of EIA recommendations and celebrating equity impacts of EIAs were perceived to help institutionalize equity assessments into organisational processes and practices. Such leadership commitment also gave equity-passionate staff a platform to advocate for equitable policy action and help bring others on the journey of doing so. Prior studies have highlighted the power of individuals, showing that people who opt in to participate in equity assessments and/or are positive about the process, were more likely to contribute to high-quality assessments [24]. Therefore, establishing and nurturing networks of equity advocates across an organisation is critical.

Our study identified implementation support as a key facilitator for effective EIAs, including a clear and concise EIA toolkit, support to access and utilise relevant data and information, and case-studies and examples of strategies and policy levers to address individual-level and structural-level barriers to health equity. Previous literature has also emphasized the importance of high-quality equity assessment tools customized for their specific implementation context [38]. Similarly, increased internal and external collaborations have been identified as both an outcome and facilitator of the EIA process and its impact on policies and organizational capacity, both in our study and others [24, 26, 27]. Structural supports that promote collaboration with equity-specialist roles (e.g. multicultural officer), committees (e.g. diversity and inclusion committee) and external partners were highly valued. However, it is crucial to consider the influence of power dynamics within these structures, including how patriarchy, ableism, colonialism, homophobia, and racism may affect the quality of such engagements and safety of individuals involved [7]. Furthermore, the feasibility of implementation support required to conduct high-impact EIAs must be considered. For some local governments, particularly those with fewer resources, additional funding and resource support from state governments and/or non-government health promotion agencies may be required.

### Strengths and limitations

To our knowledge, this is the first study to evaluate a health equity impact assessment tool (used across both health policies and other policy areas) in a local government context. The study was informed by health equity-focused evaluation and intersectionality frameworks to guide a comprehensive understanding of the impact and enablers of effective EIAs. The mixed methods study design allowed for a rich and robust analysis of data from documents, survey and interviews. Notwithstanding, the evaluation was conducted in a large, regional local government in Australia with a strong equity culture, and further research could evaluate equity impact assessment in other jurisdictions and contexts. Future evaluations could also assess the impact of equity tools as perceived by community stakeholders. 

## Conclusion

Health equity impact assessments are one tool available to help governments apply an equity lens to policy development and review processes. The capacity of these equity tools to rebalance power and resources and address both individual and structural-level causes of health inequities depends on how often, how well, when and on what types of policies impact assessments are conducted. Strong leadership and implementation support is required to drive commitment and capacity for conducting high-impact assessments. There is a role for state and federal levels of government and non-government agencies to foster equity-enabling policy environments through leadership and resources to promote wider uptake of equity tools in local governments to promote greater equity in community health.

## Supplementary Information

Supplementary Material 1.

## Data Availability

The datasets used and/or analysed during the current study are available from the corresponding author on reasonable request.
