# Correction: A realist impact evaluation of a tool to strengthen equity in local government policy-making

**DOI:** 10.1186/s12939-024-02277-2

**Published:** 2024-09-26

**Authors:** Sally Schultz, Felicity Beissmann, Christina Zorbas, Serene Yoong, Anna Peeters, Kathryn Backholer

**Affiliations:** 1 Global Centre for Preventive Health and Nutrition, School of Health and Social Development, Institute for Health Transformation, Deakin University, 1 Gheringhap Street, Geelong, VIC Australia; 2 City of Greater Bendigo, Galkangu Bendigo, VIC 189‑229 Lyttleton Terrace, Bendigo, Australia

**Correction: Int J Equity Health 23, 179 (2024)**

10.1186/s12939-024-02266-5.

Following publication of the original article [1], the authors reported that the author names were in wrong display order. The author names have been updated from Schultz Sally, Beissmann Felicity, Zorbas Christina, Yoong Serene, Peeters Anna and Backholer Kathryn to Sally Schultz, Felicity Beissmann, Christina Zorbas, Serene Yoong, Anna Peeters and Kathryn Backholer.

The original article [1] has been updated.
