# Supplementary material for: A realist impact evaluation of a tool to strengthen equity in local government policy-making
Source: Int J Equity Health. 2024 Sep 10;23:179. doi: 10.1186/s12939-024-02266-5 (PMC11385146; doi:10.1186/s12939-024-02266-5)
Supplement: Supplementary file 1 — Supplementary Material 1. [file 12939_2024_2266_MOESM1_ESM.pdf]

City of Greater Bendigo

Equity Impact Assessments (EIAs)

The City's approach to conducting Gender Impact Assessments in order to reduce barriers to gender equality in policy, program and service delivery and satisfy the requirements of the Gender Equality Act 2020.

Version: November 2022

CITY OF GREATER BENDIGO

Purpose

The City of Greater Bendigo has developed an Equity Impact Assessment (EIA) tool, incorporating Gender Impact Assessments (GIAs), based on the Commission for Gender Equality in the Public Sector's GIA Toolkit and Template which was tested on a variety of policies, programs and services in an 18-month pilot.

EIAs are applied to policies, programs and services that have a direct and significant impact on the public in their development and review. EIAs enable the City to deliver more equitable policies, programs and services and fulfil its legislative obligations under the Gender Equality Act 2020 (the Act). The Act aims to ensure the Victorian public sector makes meaningful progress towards gender equality in the workplace and the community.

The purpose of this document is to share the City's approach to implementing GIAs with other local governments and defined entities covered by the Act. It is the City's hope and intention that organisations feel free to use and adapt elements that might work well within their settings and organisational contexts.

Special thanks

The City of Greater Bendigo thanks the Commission for the GIA Toolkit and Template which was a critical resource in the pilot and helped the City arrive at a final suite of EIA materials suited to its local government context.

This City is grateful for the collaborative and collegial spirit that the local government sector and other defined entities have demonstrated in seeking to implement the Act.

It is from engagement with the City of Darebin that the City adopted a similar approach of developing an organisational-wide Social Justice Framework and an EIA tool, incorporating GIAs, to put the framework into action and promote equity, inclusion and human rights in the community. The City also adapted Step 1 of the GIA Template to occur in a workshop environment due to the City of Darebin's observation that brainstorming the policy, program or service amongst a diverse cohort of staff helped to facilitate transformative change in the EIA process.

The City thanks the Kingston City Council for generously sharing their approach of utilising a smart survey that supports staff to determine whether a policy, program or service legislatively requires a GIA.

The City also thanks The Equality Institute for conducting an external evaluation of the draft EIA materials and for providing helpful recommendations which added further rigour and ensured gender equality remained core to the process as the tools were expanded to incorporate a place-based and experience lens. We also thank the Equality Institute for helping to develop organisational fluency by providing a list of frequently used terminology.

Contact

If any organisations would like to access any of the original files or discuss the EIA materials included in this document, please reach out to ge@bendigo.vic.gov.au

EIA materials

1 **Social Justice Framework 2022-2032: Supporting Equity, Inclusion and Human Rights**

Provides an organisational-wide framework that contextualises the EIA tool and process.

2 **'Is an EIA required?' smart survey**

Independently determines in real-time whether a policy, program or service requires an EIA.

3 **The Equity Impact Assessment (EIA) Guide**

Provides an overview of the EIA process for staff.

4 **EIA Template A – Policies**

Template that uses language suited to policies in which community members are impacted by the policy rather than direct users of it.

5 **EIA Template B – Programs and services**

Template that uses language suited to programs or services in which community members directly engage, access or use the program or service.

6 **EIA Workshop: Step 1 (Template A)**

A workshop facilitation tool to conduct Step 1 which is facilitated by an EIA Champion. A workshop environment allows for educational components to be incorporated and diverse perspectives to be considered as staff define the issue and challenge assumptions related to the policy, program or service.

7 **EIA Final Workshop**

A workshop template presented by the EIA lead (policy/program/service lead) to close the loop with the participants of the EIA Workshop: Step 1 at the conclusion of each EIA. This final workshop supports EIAs to be conducted at a high standard and within reasonable timeframes by embedding peer accountability and a clear finish date.

8 **EIA Champion Role Description**

EIA Champions are staff members who are trained to support other staff conduct EIAs. They ensure EIAs are conducted in a way that is legislatively compliant, facilitate workshops and providing mentoring support.

9 **EIA Case Studies – a selection**

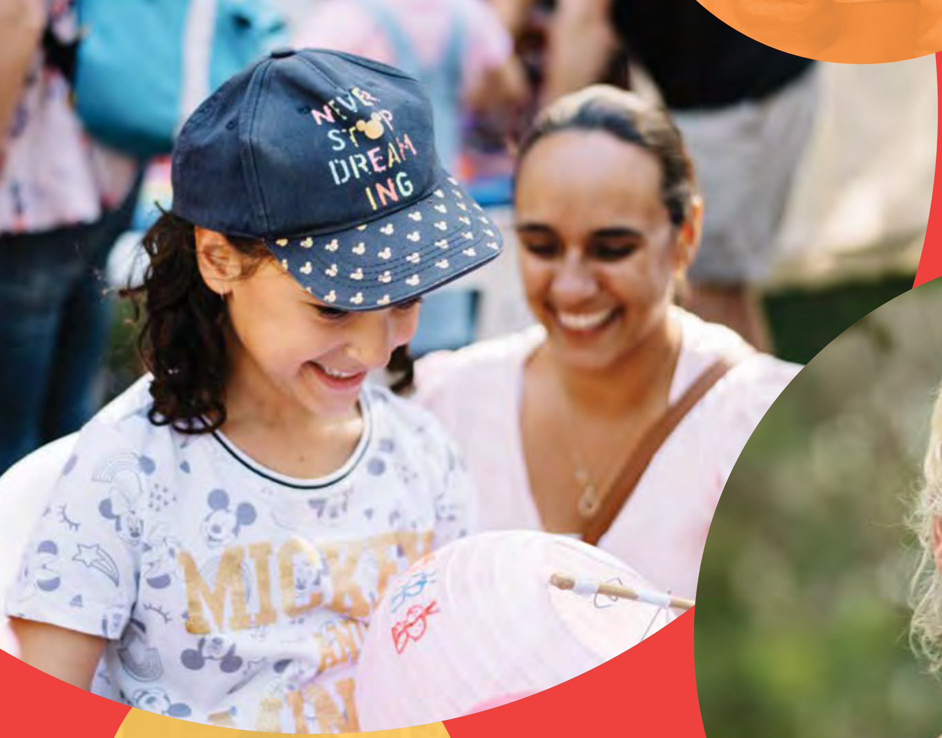

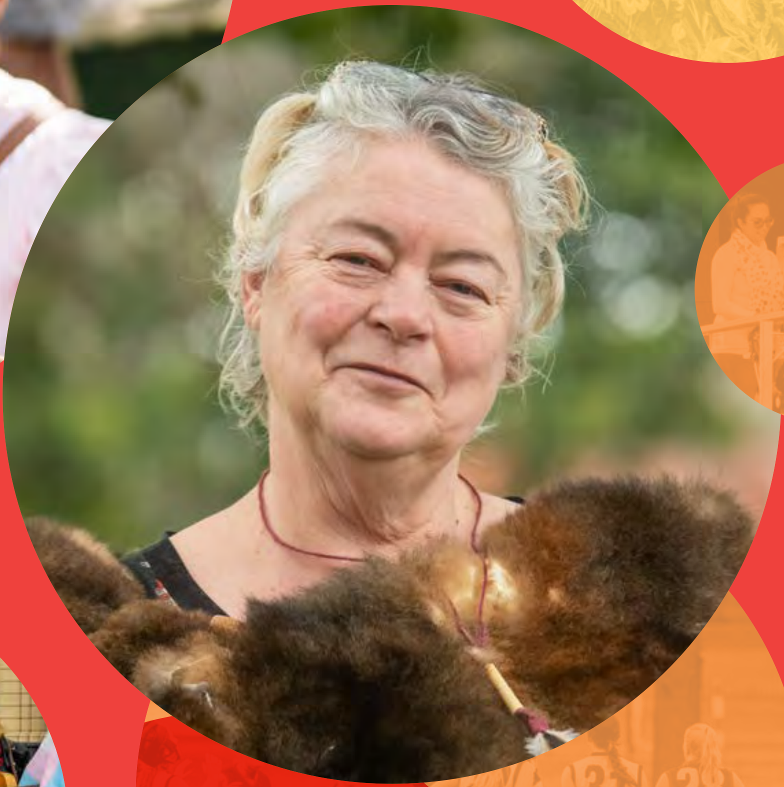

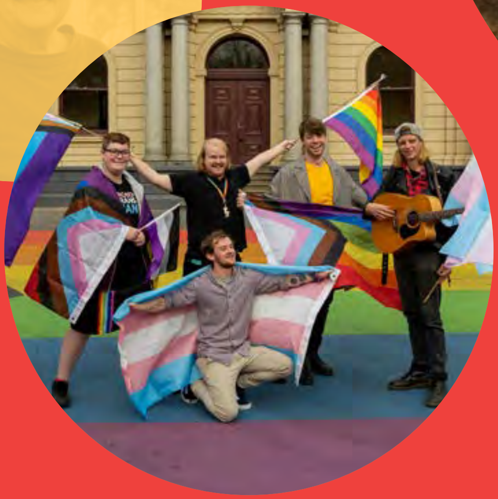

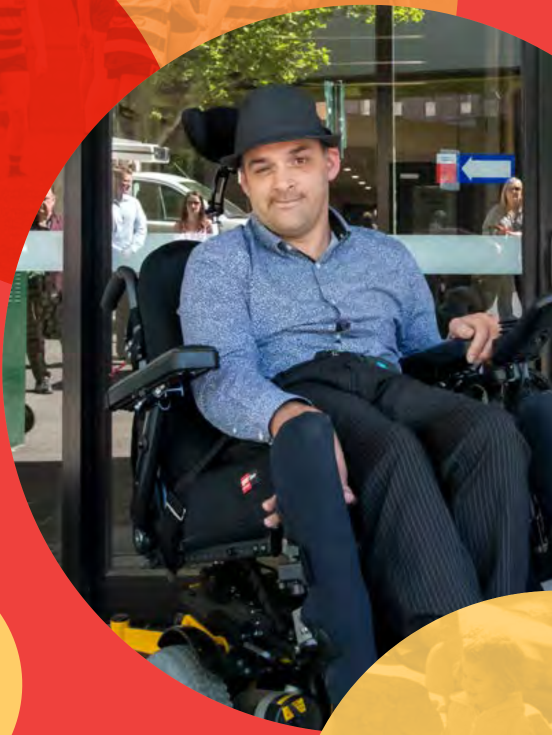

Social Justice Framework

2022
2032

Supporting Equity, Inclusion and Human Rights

CITY OF GREATER
BENDIGO

Contents

1 Message from the Mayor

2 Introduction

3 What is social justice?

4 Evaluation of the City's Human Rights Charter

5 Why is a Social Justice Framework important now?

6 Social Justice Framework principles

7 Why is social justice important for Greater Bendigo?

8 Role of the City of Greater Bendigo

9 Social justice commitments of the City

10 Social justice in action at the City

11 Social justice commitments

12 Social justice statistics for Greater Bendigo

14 Approach

18 Groups at risk of discrimination or disadvantage

18 Barriers to social justice

19 Outcomes

21 Implementation

22 Evaluation and monitoring

23 Appendix 1: Policy context

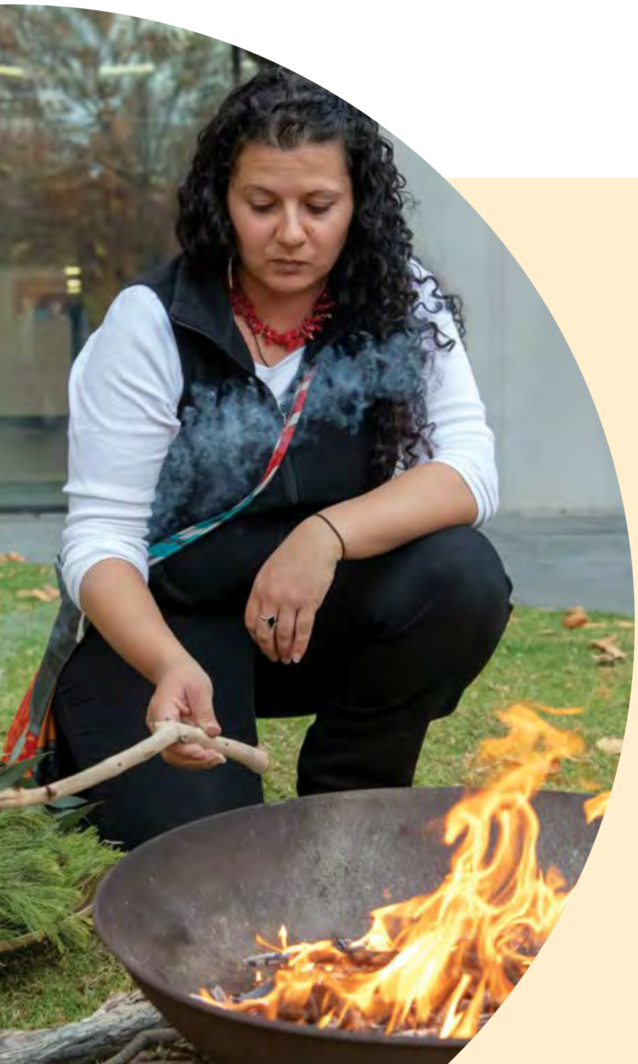

Acknowledgement of Country

The City of Greater Bendigo is on Dja Dja Wurrung and Taungurung Country. We would like to acknowledge and extend our appreciation to the Dja Dja Wurrung and Taungurung Peoples, the Traditional Owners of the land. We pay our respects to leaders and Elders past, present and emerging for they hold the memories, the traditions, the culture and the hopes of all Dja Dja Wurrung and Taungurung Peoples. We express our gratitude in the sharing of this land, our sorrow for the personal, spiritual and cultural costs of that sharing, and our hope that we may walk forward together in harmony and in the spirit of healing.

Acknowledgement of First Nations People

The City recognises that there are people from many Aboriginal and Torres Strait Islander communities living in Greater Bendigo. We acknowledge and extend our appreciation to all First Nations Peoples who live and reside in Greater Bendigo on Dja Dja Wurrung and Taungurung Country, and we thank them for their contribution to our community.

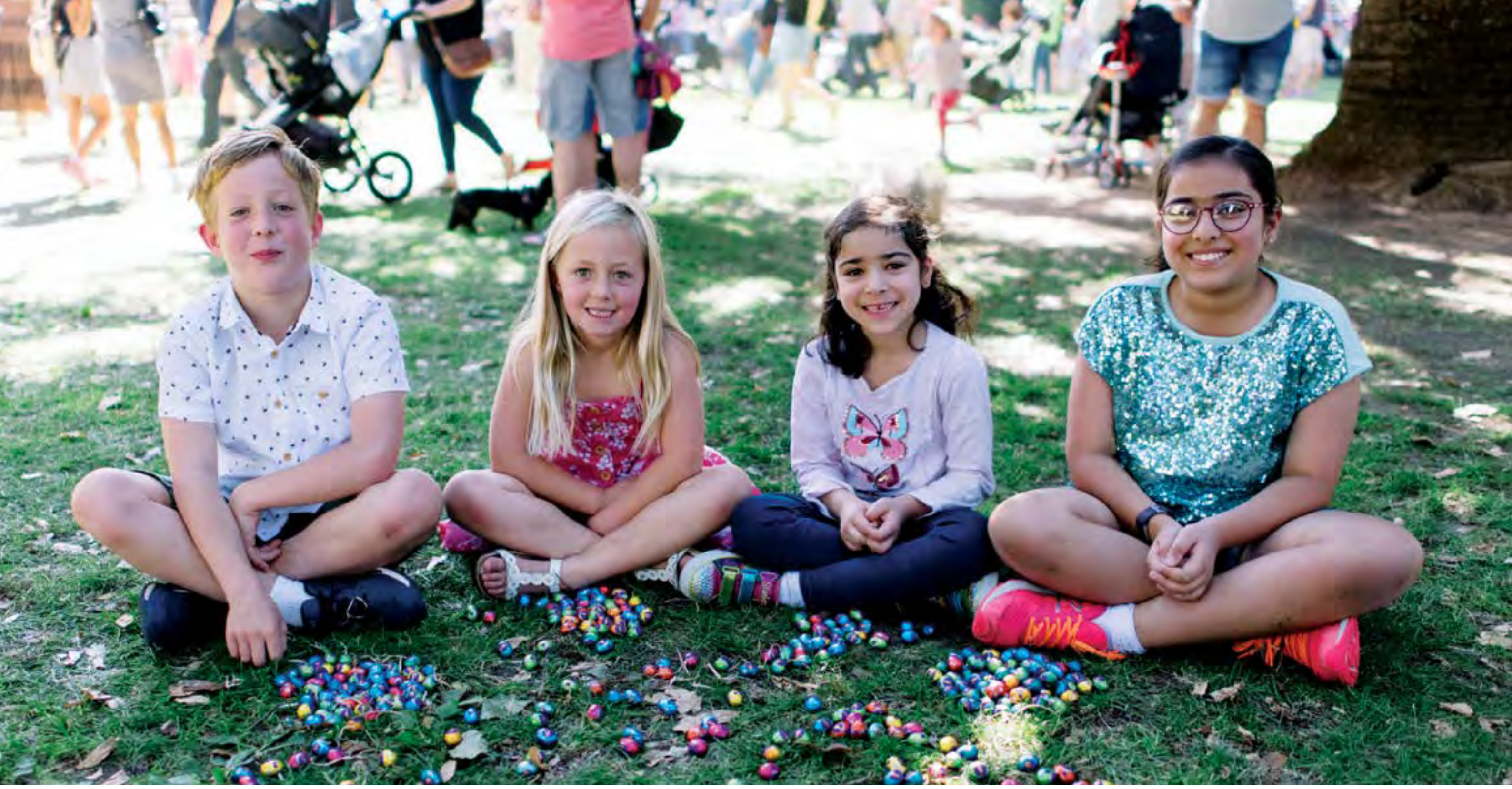

"Social justice is an underlying principle for peaceful and prosperous coexistence... We uphold the principles of social justice when we promote gender equality or the rights of indigenous peoples and migrants. We advance social justice when we remove barriers that people face because of gender, age, race, ethnicity, religion, culture or disability. – *United Nations, World Day of Social Justice*"

Message from the Mayor

The City of Greater Bendigo is pleased to present its Social Justice Framework - Supporting Equity, Inclusion and Human Rights (2022-2032).

The Social Justice Framework builds on the City's earlier Human Rights Charter (2014) and will assist our work over the next 10 years to achieve our community vision of: *Greater Bendigo celebrates our diverse community. We are welcoming, sustainable and prosperous. Walking hand-in-hand with the Traditional Custodians of this land. Building on our rich heritage for a bright and happy future.*

Fulfilling the community vision also requires a commitment to social justice. The Social Justice Framework seeks to ensure that as Greater Bendigo develops as a community it is equitable and fair, dedicated to inclusion and embraces diversity.

The City is committed to supporting all people to achieve their full potential and to ensure they have equitable access to services and opportunities and are treated fairly, equitably and with respect.

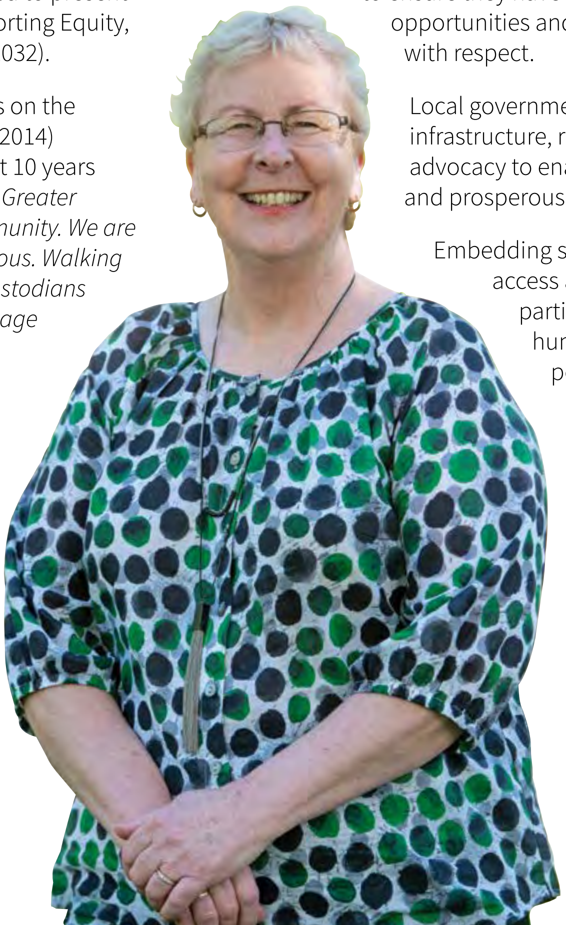

Local government is well placed to provide infrastructure, resources, services, support and advocacy to enable people to lead healthy, happy and prosperous lives.

Embedding social justice principles of equity; access and inclusion; community participation in decision making; and human rights in all of the City'ss policies, plans, strategies, programs and services is key to furthering inclusion and social justice.

I look forward to the implementation of the Social Justice Framework as we support equity, inclusion and human rights, and ensure Greater Bendigo is a fair and welcoming city for all.

Mayor Cr Andrea Metcalf

Introduction

The City of Greater Bendigo's Social Justice Framework (2022-2032) aims to provide Council and the City of Greater Bendigo (the City) with principles to guide decisions, planning and actions to improve equity and inclusion and support human rights in the community over the next ten years.

The Social Justice Framework was developed following an evaluation of the City of Greater Bendigo Human Rights Charter (2014); a review of relevant Local, State and Federal government legislation; analysis of local data; assessment of social justice and human rights best practice within other local government authorities; and engagement with the community and key stakeholders. Embedding effective processes alongside the Social Justice Framework, will assist in ensuring an integrated planning whole-of-Council approach to progress equity, inclusion and human rights. A social justice action plan and report card will provide an overview of actions that will be led or supported by the City (across multiple Council plans) to ensure an integrated whole-of-Council systems change approach to advance social justice.

The City of Greater Bendigo has a long-standing commitment to human rights. It developed and adopted the **City of Greater Bendigo Human Rights Charter** in 2014. In 2020, the City was the first local government in Australia to gain accreditation from Welcoming Cities. This recognised the City's commitment to embracing diversity and building on the communities' strengths to create a social, cultural, economic and civic cohesion within a sustainable natural environment.

In 2021, Greater Bendigo developed its community vision: *Greater Bendigo celebrates our diverse community. We are welcoming, sustainable and prosperous. Walking hand-in-hand with the Traditional Custodians of this land. Building on our rich heritage for a bright and happy future.*

The Greater Bendigo community vision is underpinned by values strongly connected to social justice, including:

- Equity – provide support and give opportunity to others to be part of this community
- Inclusion – capturing our cultural and community diversity

The **Greater Bendigo Community Vision and Values (2021-2030)** are the basis of the **Council Plan 2021-2025 *Mir wimbul***, which embeds the social justice principles of equity, fairness and inclusion as a priority in Council's planning, policies, programs, and services. One specific objective in *Mir wimbul* was to "Implement the recommendations of the Bendigo Human Rights Charter evaluation" (Objective 7.6.1, p.26) and one action was to "Develop a Social Justice Framework" (Action 7.6.1, p.26). The **Municipal Health and Wellbeing Plan (Healthy Greater Bendigo 2021-2025)** has a guiding principle to 'promote social justice'.

Alongside this local context is the City's responsibilities under State and Federal government legislation (See Appendix 1: Policy Context, p.25). For example, the Victorian *Local Government Act 2020* legislates that "services should be provided in an equitable manner and be responsive to the diverse needs of the municipal community" (section 106 (2) a and b).

This Social Justice Framework is the outcome of these four elements. It builds on the previous Greater Bendigo Human Rights Charter to support a continuing culture of human rights while drawing on both the Charter evaluation; Greater Bendigo's Community Vision and Values; and new legislative requirements, to create a broader social justice framework which will progress equity, fairness and inclusion in Greater Bendigo.

Addressing inequity through local policies, plans and programs is key to furthering inclusion and social justice. Local government is well placed to provide infrastructure, resources, services, support and advocacy to enable people to lead healthy, happy and prosperous lives.

The City is committed to supporting people to achieve their full potential and to ensure all the community has equitable access to services, opportunities and every person is treated fairly, equitably and with respect.

> "It has become increasingly evident that inequality manifests itself most clearly at the local level.[1]"

1 Benoy Jacob, *Governing for Equity: Implementing an Equity Lens in Local Government*, ICMA, 2020.

What is social justice?

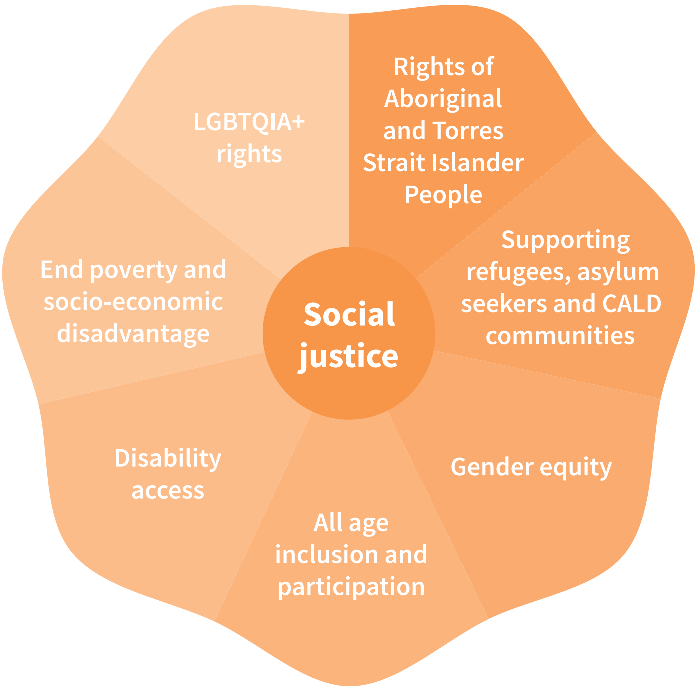

Social justice is the pursuit of equal rights, equal opportunity and human rights for all people in our community. Social justice embraces the concept of social inclusion and the principles of providing access, equity, participation and human rights to all people in our community. Social Justice acknowledges diversity, discrimination and disadvantaged groups in the community. Social justice principles address the impact that social and economic inequalities have on both the people experiencing them and on the wider community. Social justice incorporates community participation in decision making that impacts on their lives.

The shift from a narrower focus on protecting human rights to a broader emphasis on addressing social justice responsibilities is a significant change which has occurred across local government. This shift is reflected in the Victorian *Local Government Act's* requirement of equitable service provision responsive to community diversity, but also under the *Public Health and Wellbeing Act 2008*. Under this Act, Parliament recognises that 'public health interventions are one of the ways in which the public health and wellbeing can be improved and inequalities reduced'. One responsibility of councils under the Act, is 'to create an environment that supports the health of members of the local community and strengthens the capacity of the community and individuals to achieve better health'.

Fulfilling the Greater Bendigo community vision of celebrating our diverse community and being welcoming, sustainable and prosperous, requires a commitment to social justice.

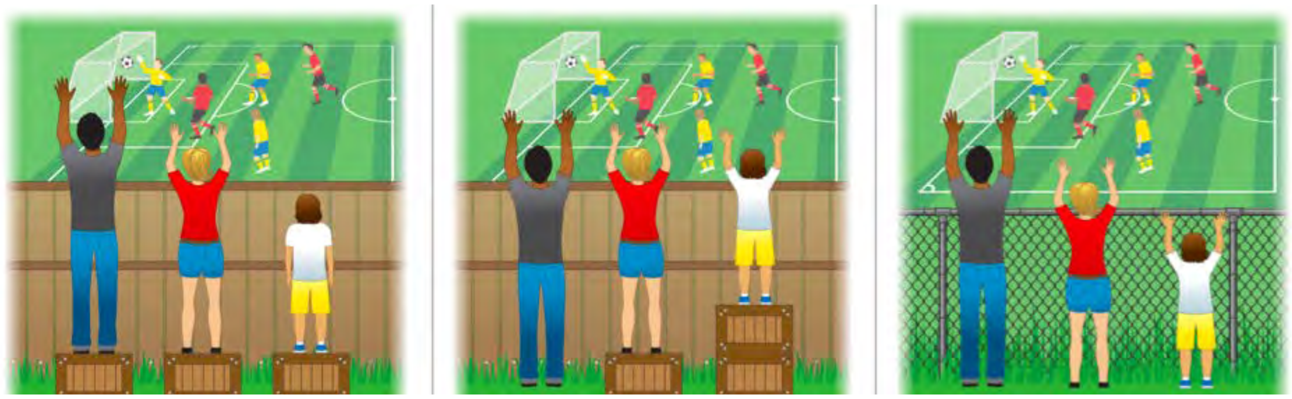

Equality

The assumption is that **everyone benefits from the same supports**. This is considered to be equal treatment.

Equity

Everyone gets the supports they need, which produces equity. This is the concept of affirmative action.

Justice

All three can see the game without supports or accommodation because **the cause(s) of the inequity was addressed**. The systemic barrier has been removed.

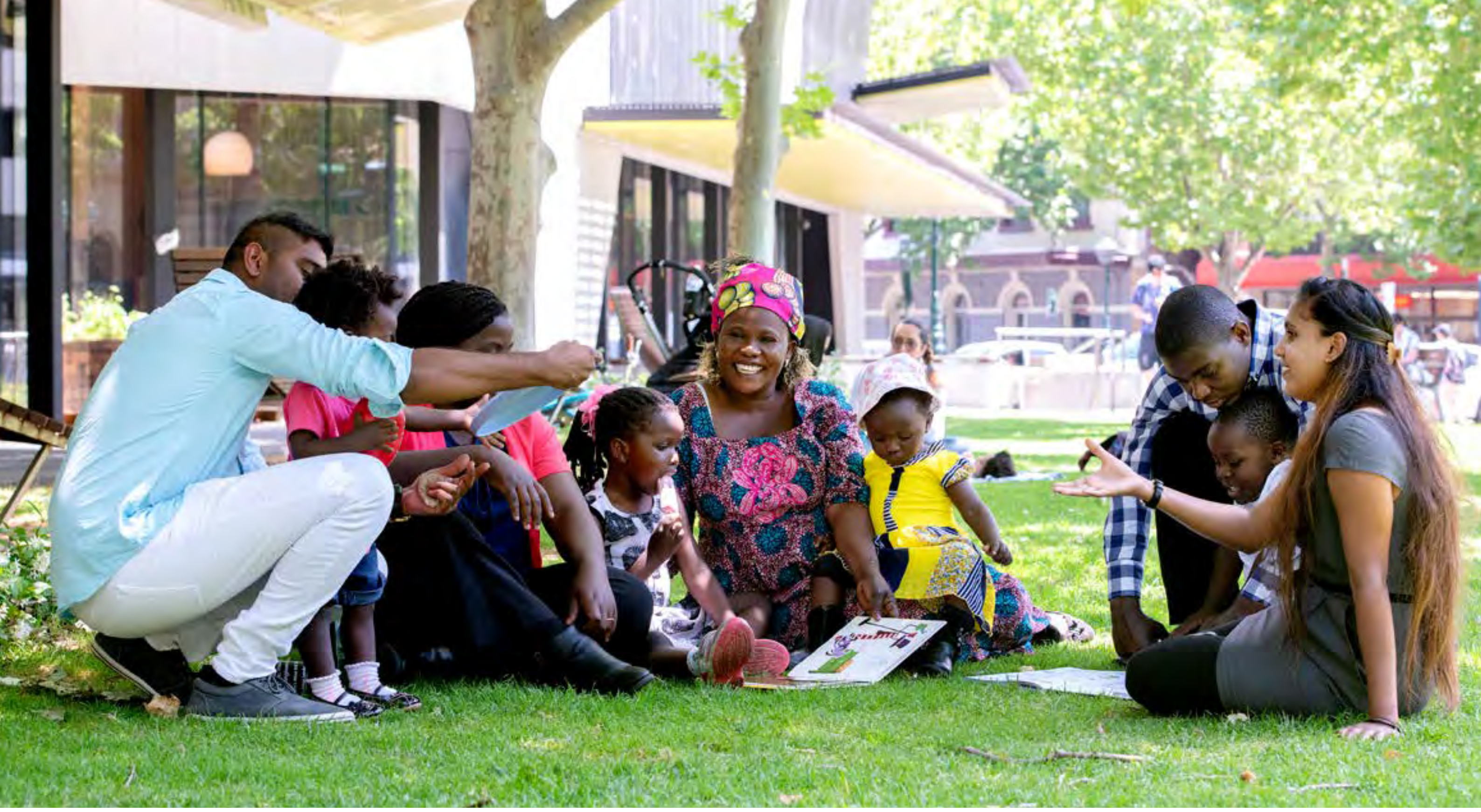

Evaluation of the City's Human Rights Charter

The **City of Greater Bendigo Human Rights Charter (2014)** reflected the *Victorian Charter of Human Rights and Responsibilities Act 2006*.

It was formulated during a time of protests for and against Council's decision to grant a planning permit to build the Bendigo Islamic Community Centre. The Charter developed a framework for the City and Council to meet its responsibilities under State and Federal government legislation and promote human rights via three areas: Compliance, Culture and Advocacy. The Charter focussed on four key areas: Freedom, Respect, Equality and Dignity.

In 2021, the evaluation of the Human Rights Charter noted the positive impact of the Greater Bendigo Charter in:

- Guiding Council when making decisions, adopting resolutions and making public statements
- Serving as a valuable guide to Council when promoting human rights to the Greater Bendigo community
- Aiding the City when advocating the region's interests to State and Federal authorities
- Setting standards for City staff to adhere to when performing their duties
- Contributing to a human rights culture in Greater Bendigo

The Evaluation also noted that since the Council adopted the Human Rights Charter *"The understanding of human rights has evolved, as has the community with respect to demographics and community attitudes. An updated Charter will ensure it maintains relevance to current community issues. Also, the new Local Government Act (2020) states that: 'Councillor Codes of Conduct must comply with the Victorian Human Rights Charter'. The Act has increased requirements for community engagement using the principles of deliberative engagement. These align with the values of equity and social justice."*

The evaluation concluded that *"Based on benchmarking the approach taken by other local governments, a focus on social justice provides clearer guidance to policy and strategy formulation, positioning the local government to directly redress vulnerabilities within their constituency. The social justice approach builds upon human rights with a focus on reducing vulnerabilities through addressing the barriers to equality in the economic, environmental, social and cultural domains."*

The two primary recommendations were:

1. To update the Human Rights Charter to be the City of Greater Bendigo Social Justice Framework
2. Develop an Action Plan to embed the Social Justice Framework throughout the City's operations

Why is a Social Justice Framework important now?

The value and purpose of a Social Justice Framework for Council is to:

- Ensure Council meets its obligations of progressing equity, inclusion and human rights, as required by State legislation
- Provide a mandate for Council to embed social justice principles of equity; access and inclusion; community participation in decision making; and human rights in all of the City's policies, plans, strategies, programs and services
- Enable Council to take a position of leadership by establishing clear positions on equity, access and inclusion, participation, human rights and social justice
- Support the City's social justice advocacy across Government, industry, business and community
- Facilitate the City bringing together key stakeholders to address discrimination and inequity
- Enable City partnerships with key stakeholders to leverage assets and resources to address social and structural barriers to achieving equity, access and inclusion and social justice

As well as the need to more effectively meet the requirements of recent State legislative change (*Victorian Local Government Act 2020* and *Gender Equality Act 2020*), five other contexts make a social justice framework in 2022 important:

- Incidents of racism against culturally diverse residents and communities in Greater Bendigo increased during COVID-19
- COVID-19 has contributed to increased inequity and disadvantage in Greater Bendigo
- Climate change (including issues of rising energy costs and heat health) will affect socio-economic disadvantaged families more severely
- The ongoing social change required to address violence against women and children; address gender power disparities; and advance gender equity
- A growing population who may bring different values and perspectives, and increasing diversity in Greater Bendigo, makes a statement outlining what Council stands for

A Social Justice Framework is crucial in providing a set of principles, which can guide Council, as the previous City of Greater Bendigo Human Rights Charter did.

In 2020, Mayor Cr Margaret O'Rourke spoke out publicly against COVID generated racism and abuse against Karen and other Asian-Australian residents in Greater Bendigo, declaring it to be "deeply disappointing and unacceptable."

"It is not who we are and it is not who we want to be. We are a welcoming, inclusive and safe community that celebrates our growing diversity. The commitment to equity and respect as outlined in our Humans Rights Charter, means we wholeheartedly reject racism and discrimination of any sort. There is no room for racism or discrimination here."

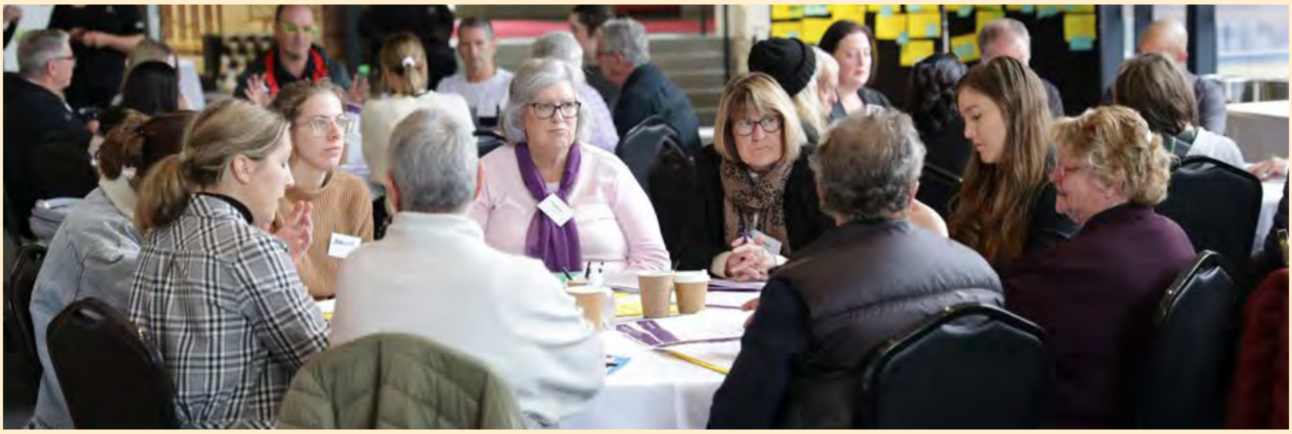

Social Justice Framework principles

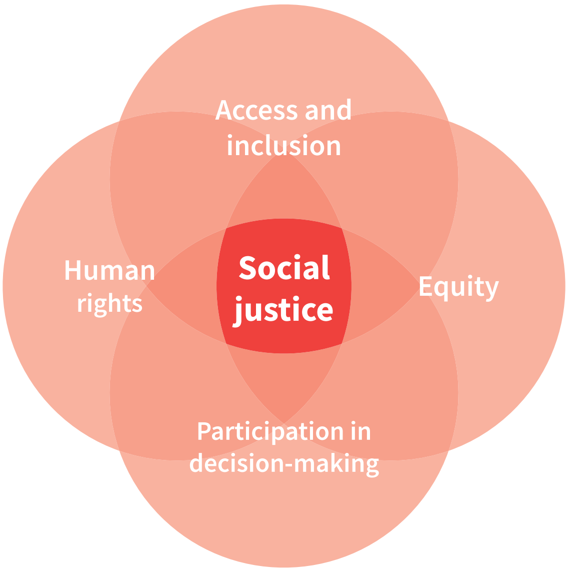

The Social Justice Framework is a set of principles, based on Greater Bendigo's community vision and values, that will guide the City's work enabling Greater Bendigo to fulfil its vision of celebrating our diverse community and being welcoming, sustainable and prosperous, while walking hand-in-hand with the Traditional Custodians.

The key principles are:

- **Human Rights**
- **Access and Inclusion**
- **Equity**
- **Participation in decision-making**

These core principles are supported by seven pillars of action:

1 Recognise Aboriginal and Torres Strait Islander Peoples/First Nation's peoples

The City of Greater Bendigo acknowledges the Dja Dja Wurrung and Taungurung Peoples as the Traditional Owners of the land and recognises other Aboriginal and Torres Strait Islander people as First Nations Peoples; and values their traditions, knowledge and culture.

2 Uphold Human Rights

The City of Greater Bendigo is bound by the provisions of the *Victorian Charter of Human Rights and Responsibilities Act 2006*. The City's actions ensure a welcoming community, where dignity, equality and mutual respect are shared across cultures, religions, and beliefs.

3 Champion Social Justice

The City of Greater Bendigo advocates for equity, access and inclusion for all people in our community, acknowledging diversity and discrimination and disadvantage due to factors such as race/ethnicity, gender, class, age, sexuality, gender identity, disability and religion, or a combination of these factors.

4 Advance Equity

The City of Greater Bendigo addresses inequity by dealing with the impact that social and economic inequalities have on the municipal community and prioritising the available economic, social and political resources to those who are most disadvantaged.

5 Support Access and Inclusion

The City of Greater Bendigo services, programs and facilities are designed and delivered in a way that is available to all and incorporates diverse perspectives and skills, with a focus on reducing the barriers which may prevent access.

6 Enable Engagement and Participation

The City of Greater Bendigo engages the community to enable meaningful, equitable and informed input into decisions which affect their lives.

7 Support Environmental Justice

The City of Greater Bendigo commits to tackling 'environmental and climate breakdown' (recognising the serious risks to the health and wellbeing of residents, especially those who are socially and economically disadvantaged) alongside enhancing the natural environment through minimising the ecological footprint and rehabilitating natural ecosystems.[2]

---

2 The climate motion was passed by Council in August 2019 (referenced in *Climate Change and Environment Strategy, 2021-2026* and also the *One Planet Report 2019-20*).

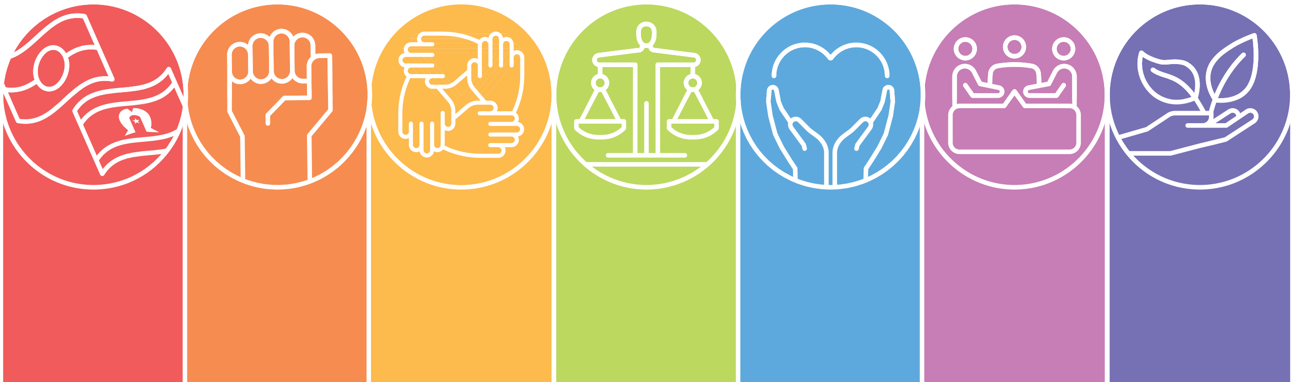

Why is social justice important for Greater Bendigo?

As the state's fourth largest city, Greater Bendigo's demographic profile reveals marginalised or discriminated groups who are affected by barriers to social justice. It also reveals areas of extremely high socio-economic disadvantage. **A Stronger Greater Bendigo** pointed to 'a growing geographical and social gradient across Greater Bendigo expressed in measures of wealth, access to facilities and services, health outcomes, and participation levels in education and employment'.[3] It emphasised that disadvantage had 'become intergenerational and entrenched' for many people.

Having an unequal community impacts on the whole community, as a steep social gradient has an overall impact on community health and wellbeing; on the liveability of a community; and on the local and regional economy.

---

3 A Stronger Greater Bendigo p. 58.

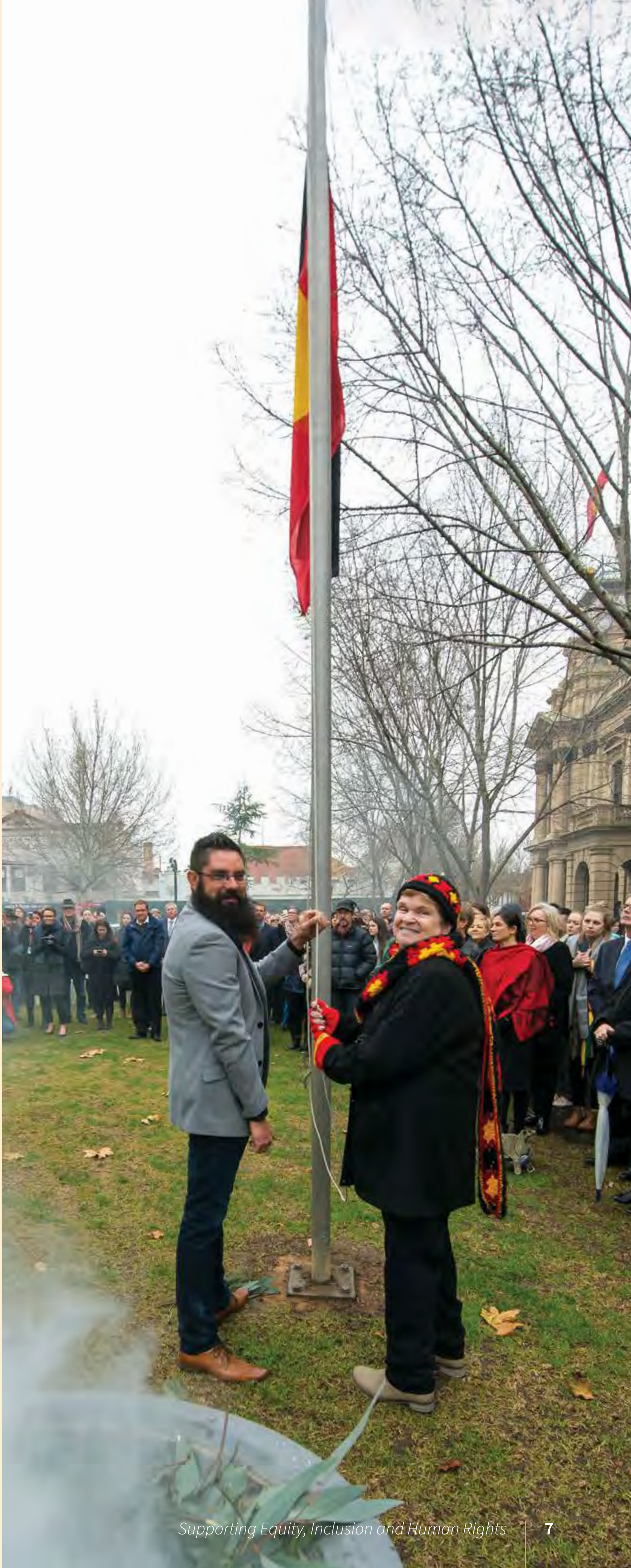

Role of the City of Greater Bendigo

The City has a strategic responsibility to plan, lead, advocate for and work with the local community for social justice, as it is the level of government closest to the community and has legislated requirements. It will do this through the following roles:

- **Planner** - Developing plans and strategies which proactively address barriers that entrench disadvantage and inequality and focus on improving equity; access and inclusion; community participation in decision making; and human rights
- **Leader** - Establishing a clear position on equity, access and inclusion, participation and human rights and building an increased understanding of social justice issues
- **Advocate** - Advocating across all levels of Government, industry, business and community to improve outcomes in all areas relating to social justice
- **Engager** - Engaging with the local community to ensure that those experiencing disadvantage, inequity and discrimination have a voice and input into proposed plans and priorities for programs and services which affect them
- **Facilitator** - Connecting groups and bringing them together to build tolerance and understanding of difference and celebrate diversity; as well as bringing together key stakeholders to address discrimination and inequity and facilitate improved outcomes in social justice
- **Partner** - Partnering with key stakeholders to leverage assets and resources to address social and structural barriers to achieving social justice

Key strategies, such as the **Council Plan 2021-25 *Mir wimbul*** and **Healthy Greater Bendigo 2021-2025**, reflect the principles of social justice. These are further supported by other strategies which have included a social justice approach and address some of the different barriers to experiencing equity, access, community participation in decision making and human rights. For example: ***Barpangu* Build Together: City of Greater Bendigo Reconciliation Plan, 2021-2025**; **Cultural Diversity and Inclusion Plan, 2021-2025**; **Equity for All (E4A) Gender Equity Action Plan, 2021-2025**; and **Affordable Housing Action Plan, 2021**. The Social Justice Framework can already be seen to have links to the above strategies and plans.

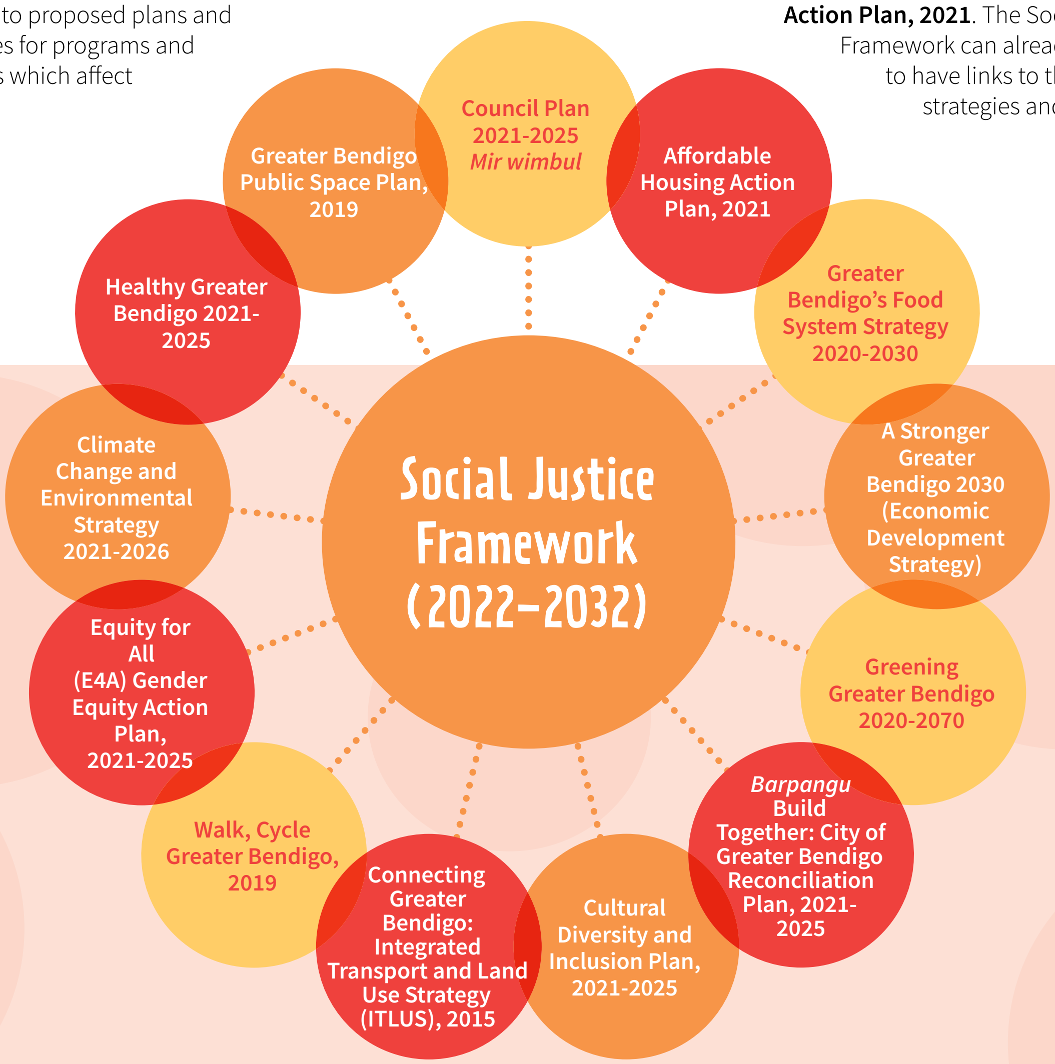

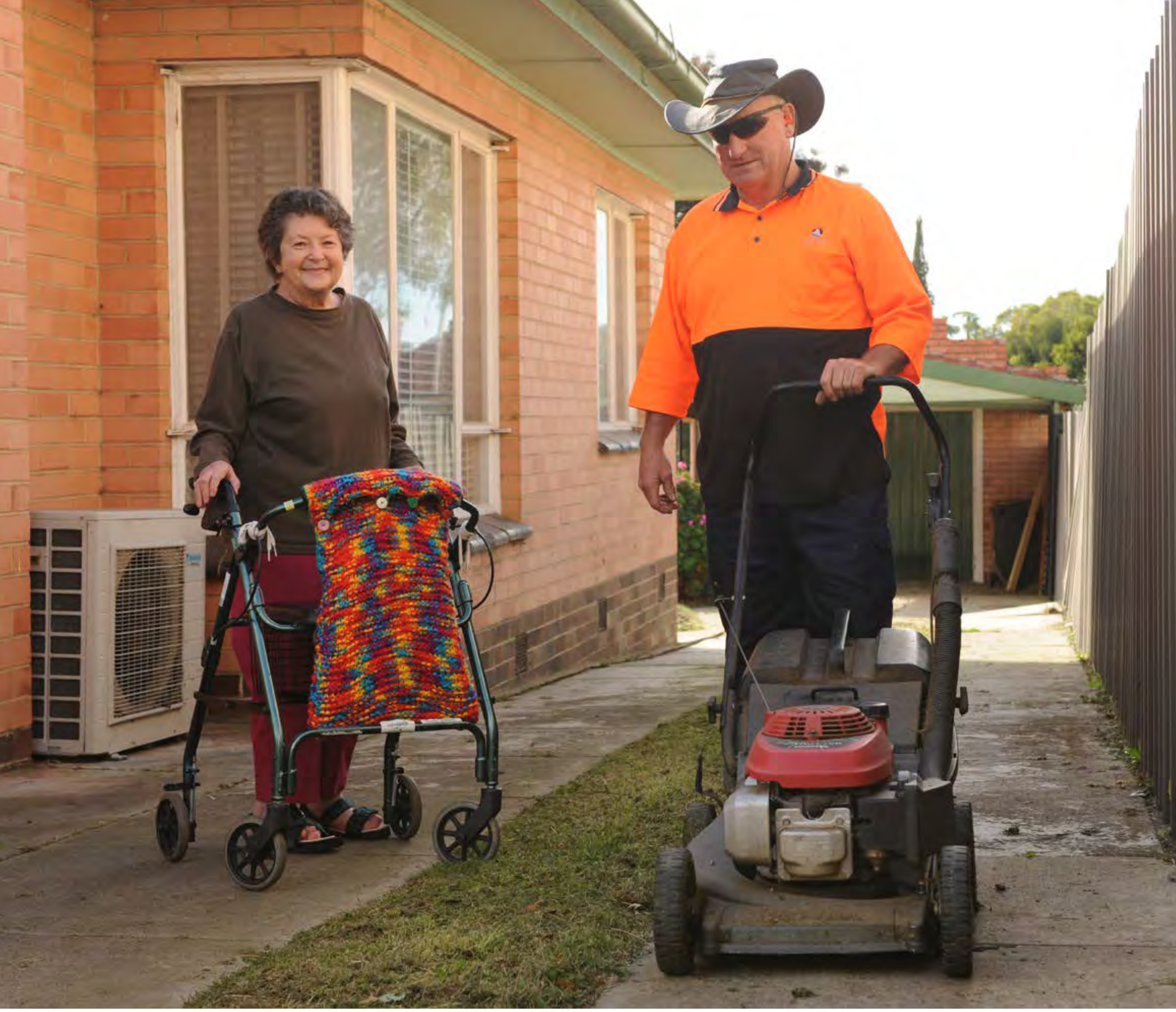

Social justice commitments of the City

Emerging from its community vision principles of equity and inclusion, the City can already be seen to have a commitment to social justice through multiple plans and strategies, ensuring a 'whole-of-Council' approach to social justice.

For example, one of the principles of **Healthy Greater Bendigo 2021-2025** is to "promote social justice" (p.4). "Everyone deserves equal rights and opportunities, including the right to good health. Many people in our community face barriers to good health that are avoidable and unjust. We need to adopt a social justice approach to address these barriers and focus on access, equity, inclusion and human rights" (p.21).

An internal Social Justice Framework Action Plan will be developed to accompany the Social Justice Framework (as recommended by the Human Rights Charter Evaluation). This Social Justice Action Plan will detail existing actions within different Council Plans, which address social justice goals and objectives, as well as include new actions focused on strengthening other policy, developing tools and building staff capability to apply the social justice principles.

The Action Plan will bring together all of Council's different social justice commitments and the measures of their successful implementation.

Social justice in action at the City

The City's commitment to social justice and human rights is evident in its actions across multiple areas of its operations, not just in its policies, plans and commitments. These are a few examples of social justice in action in Greater Bendigo, of 'walking the talk' (see annual Social Justice Report Card for more details).

Traditional Owners and Aboriginal and Torres Strait Islander Peoples

- ☑ Embedding Welcome to Country and Acknowledgement of Traditional Owners
- ☑ Recognising traditional owners and supporting their language and culture by Aboriginal place naming
- ☑ Providing Aboriginal Cultural Awareness Training and On-Country Training sessions to staff

Cultural diversity

- ☑ First local government in Australia to be accredited as a Welcoming City
- ☑ Implemented Intercultural Ambassador Pilot Program
- ☑ Facilitated Cultural Diversity and Inclusion Sports Action Group
- ☑ Supported emergency preparedness with refugee communities

Gender equity

- ☑ Introduced Gender Impact Assessments
- ☑ Introduced Gender Equality Workplace Action Plan

Families in poverty

- ☑ Developed social housing target to increase current supply of social housing

LGBTQIA+ community

- ☑ Celebrated LGBTQIA+ community through Progress Pride Flag mural; the raising of the Pride Flag on IDAHOBIT and the Transgender flag on International Trans Day of Visibility; and the support of Bendigo Pride Festival

Hardship and resource decisions

- ☑ Introduced a Budget principle of hardship
- ☑ Implemented Financial Hardship Guidelines

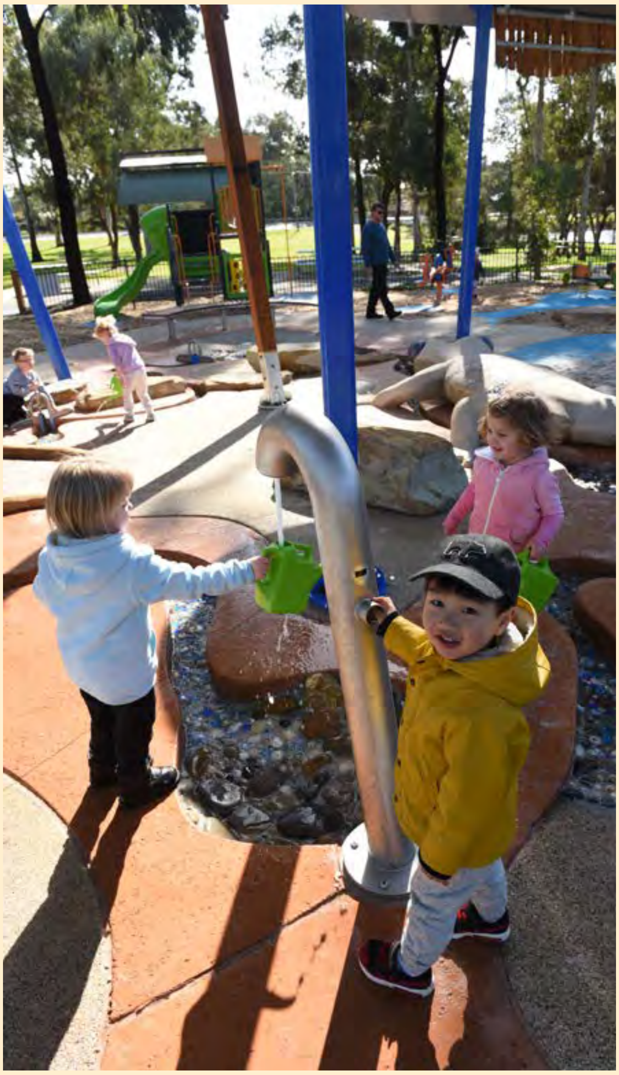

Disadvantaged places

- ☑ Healthy Heart of Victoria Kitchen Garden project established in two disadvantaged areas, Long Gully and Kangaroo Flat
- ☑ Prioritising socio-economic disadvantaged areas by investing resources in Heathcote Civic Precinct Project

People with disability

- ☑ Released Access Keys (accessibility guides with information about accessing venues, events and programs)
- ☑ Disability Inclusion Reference Committee ensured people with a disability could provide advice to Council on access and inclusion priorities

Young people

- ☑ Youth Council enabled young people to have a voice and provide advice to Council on plans, strategies, policies and projects so that they better aligned to the needs of young people

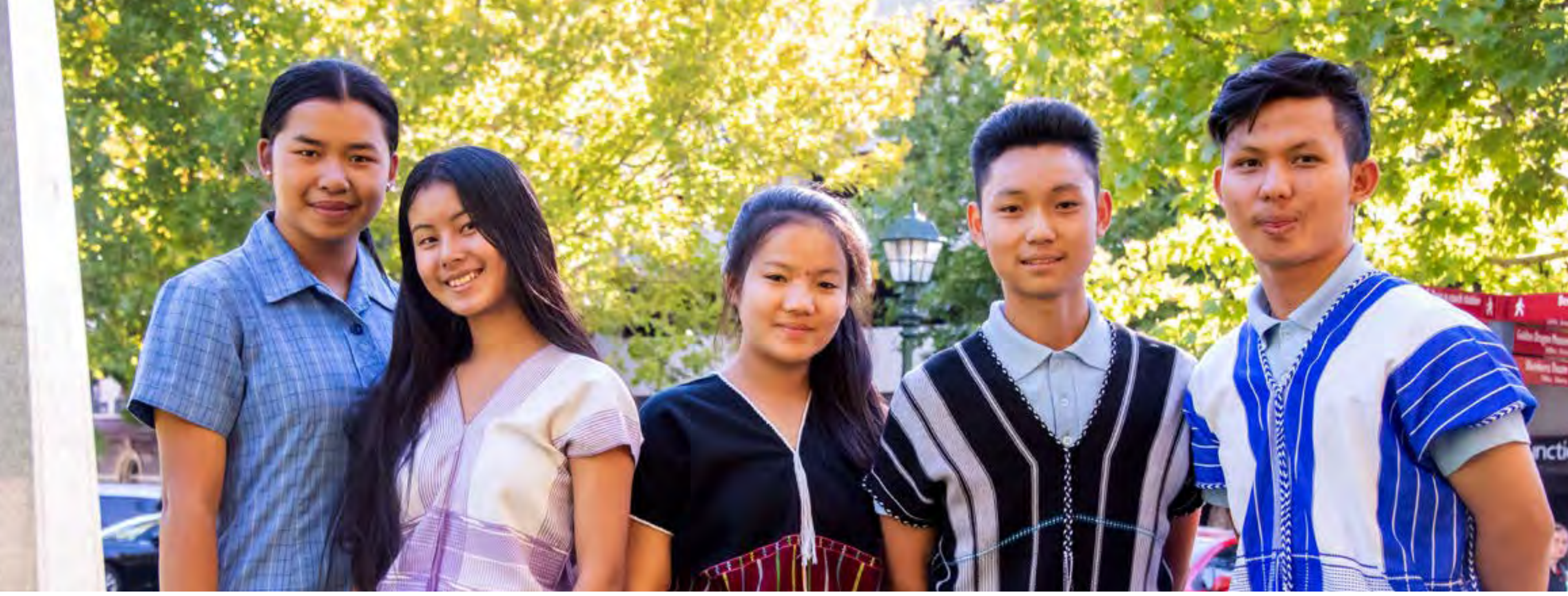

Social justice commitments

City of Greater Bendigo's Social Justice Framework focuses on people and their experiences and on place and is facilitated by systems change; integrated planning; and being strengths-based. This approach enables the framework to be responsive to the diverse needs of the community; engage effectively with different groups in the community; and provide services equitably (all required under Victorian *Local Government Act 2020*). Under the Social Justice Framework, the City makes a commitment to:

- The Dja Dja Wurrung and Taungurung Peoples as the Traditional Owners of the land and to other Aboriginal and Torres Strait Islander people in Greater Bendigo (underpinned by ***Barpangu* Build Together: City of Greater Bendigo Reconciliation Plan, 2021-2025**)
- Culturally and religiously diverse people and supporting refugees and asylum seekers by taking a stand against racism and being a welcoming community that respects cultural and religious differences and supports multiculturalism (underpinned by the **Cultural Diversity and Inclusion Plan, 2022-2026**).
- Gender equity and preventing violence against women (underpinned by **Gender Equity Action Plan, 2021-2025** and our legal obligations under the *Gender Equality Act 2020*).
- People with a disability and improving disability access across Greater Bendigo (underpinned by **Healthy Greater Bendigo 2021-2025**, and our legal obligations under the *Disability Discrimination Act 1992*).
- People experiencing mental ill health, supporting them to participate, engage and be empowered to live well (underpinned by **Healthy Greater Bendigo 2021-2025**).
- Children, supporting them as citizens with rights in a Child-Friendly City and being a 'Child Safe' organisation (underpinned by **Healthy Greater Bendigo 2021-2025**, and working to being compliant with the Victorian Child Safe Standards).
- Young people, supporting them to explore, engage and be empowered to shape the world they live in (underpinned by **Healthy Greater Bendigo 2021-2025** and the Youth Strategy).
- Older people and strengthening Greater Bendigo as an Age-Friendly City where older people are valued, supported to engage and empowered to live well (underpinned by **Healthy Greater Bendigo 2021-2025**).
- Lesbian, gay, bisexual, transgender, gender diverse, queer and intersex people and to celebrate our LGBTQIA+ community and stand up against homophobia, biphobia, transphobia and intersex discrimination (underpinned by **Council Plan 2021-2025 *Mir wimbul*** and **Healthy Greater Bendigo 2021-2025**).
- People in poverty, low-income families, or people experiencing economic hardship, homelessness or intergenerational social-economic disadvantage by addressing poverty and disadvantage (underpinned by **Affordable Housing Action Plan, 2021** and **Healthy Greater Bendigo 2021-2025**).
- Suburbs and regional areas where members of the community experience the most disadvantage by prioritising resources including infrastructure, services and programs to reduce disadvantage - and redress historical inequity in community infrastructure investment.

Social justice statistics for Greater Bendigo

2.4%
identify as Aboriginal or Torres Strait Islander[1]

51.5%
of the City's population identify as women[1]

7.6%
are living with disability[1]

1 in 10
households are food insecure, up to 1 in 5 households in some areas[4]

Rate of sexual offences against women is
double
the Victorian average[5]

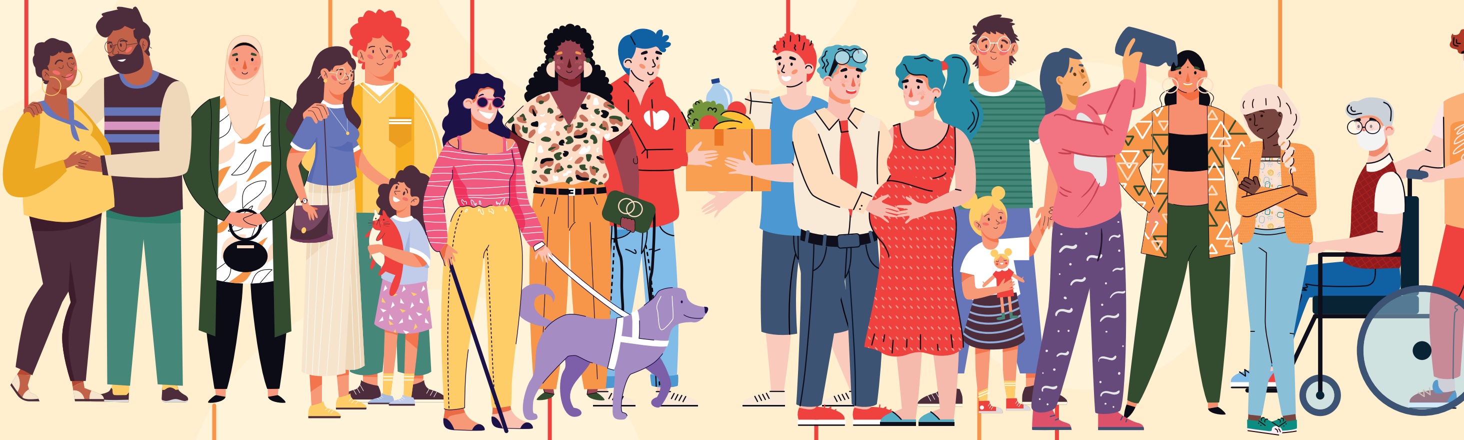

10.3%
were born overseas, 20% had a parent born overseas[1]

14%
of people are living in poverty[2]

51%
of overseas arrivals speak a language other than English at home[1]

19%
of low income households earn less than $650 per week, up to 25%-30% in some areas[3]

9 areas
in Greater Bendigo are in the top 15% of most disadvantaged Australian suburbs[2]

1 ABS, Census , 2021 - City of Greater Bendigo. **2** ABS, Census, 2016 - City of Greater Bendigo. **3** ABS, Census, 2021, 2016 - City of Greater Bendigo. **4** City of Greater Bendigo Active Living Census 2019. **5** Reported Incidents, Greater Bendigo Violence Against Women Fact Sheet, Women's Health Victoria, 2021.

36%

experience anxiety or depression[4]

14%

youth unemployment, up to 18% in some areas[2]

12%

of the City's population are young people (15-24 years)[1]

31%

of young people engaging with Headspace identified as LGBTIQ[8]

26%

of households are vulnerable to housing crisis or eviction due to rental or mortgage stress[6]

18%

are single parent families, 82% of these are women[1]

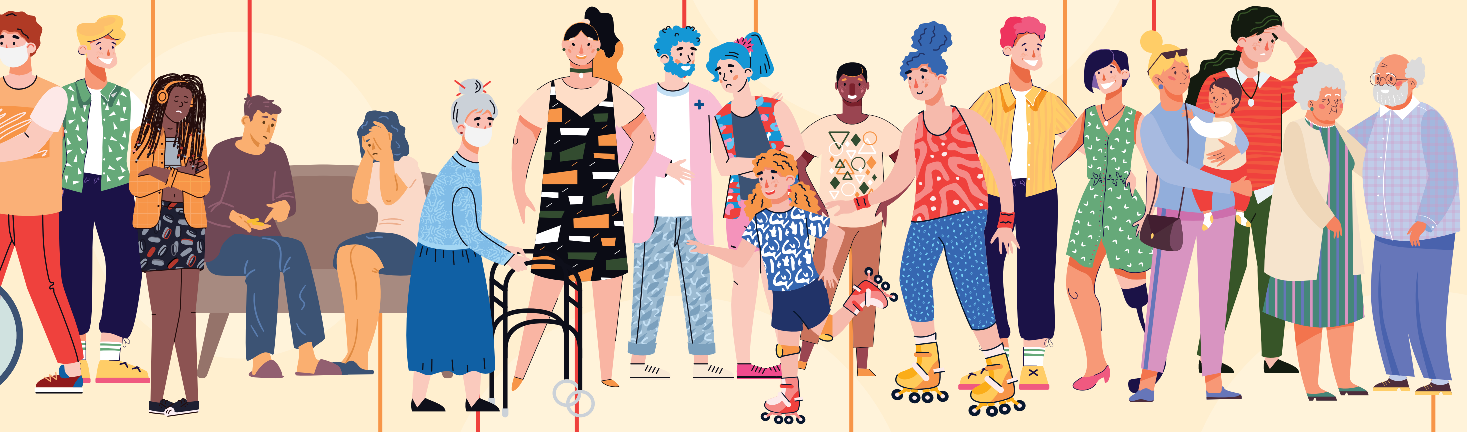

3,000

people have an acute need for social housing[6]

5.7%

identify as LGBTIQ[7]

36%

of low income families and 29% of children/young people have a cost barrier to increasing physical activity[4]

11%

are older lone person households, up to 15% in some areas[2]

16%

do not have a home internet connection, 26% in some areas[9]

6 City of Greater Bendigo Affordable Housing Action Plan, 2020. **7** Victorian Population Health Survey 2017 - City of Greater Bendigo. **8** City of Greater Bendigo Submission to Vic Gov LGBTIQ Strategy. **9** https://profile.id.com.au/bendigo/internet-connection

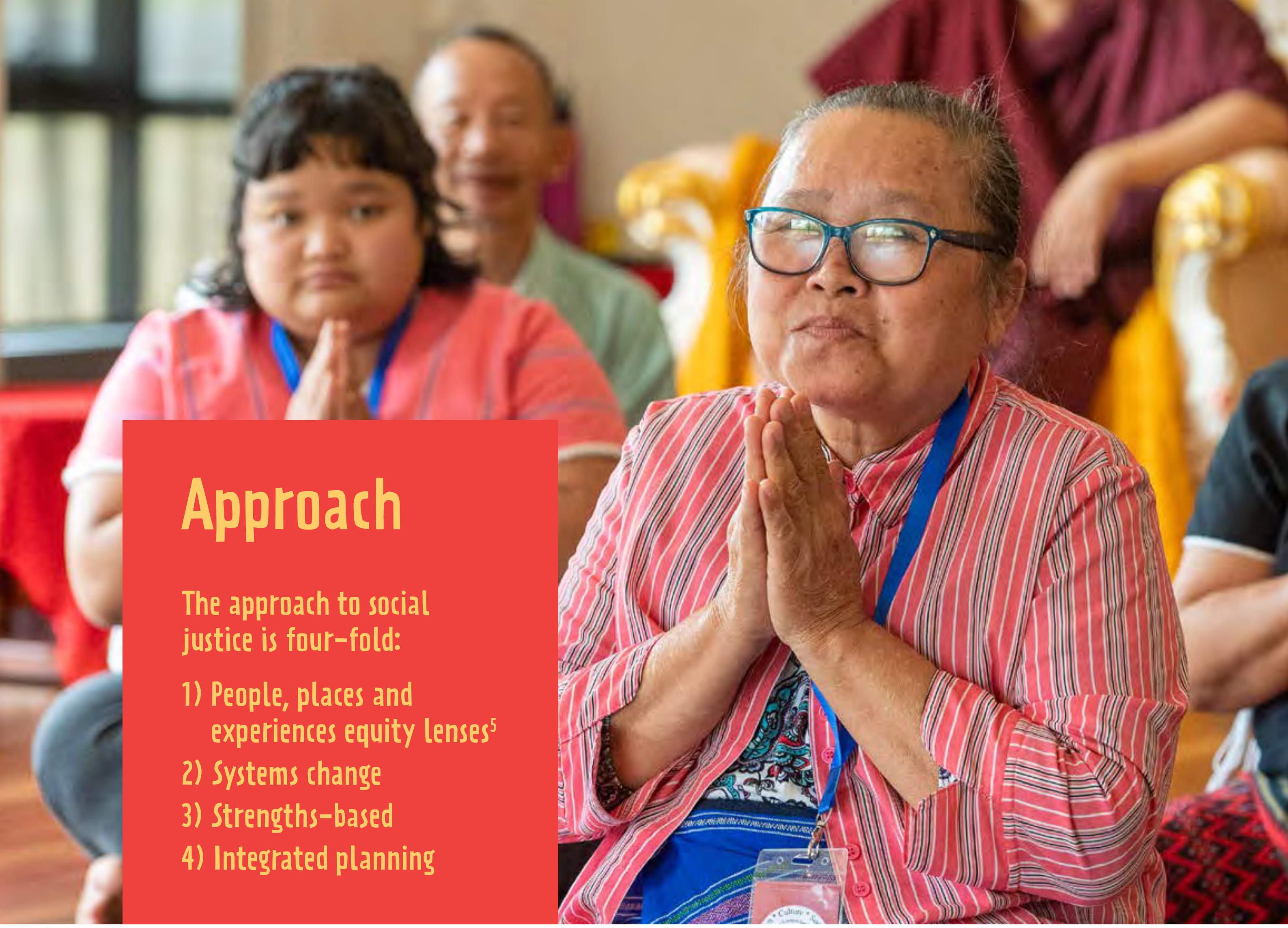

Approach

The approach to social justice is four-fold:

1) People, places and experiences equity lenses[5]
2) Systems change
3) Strengths-based
4) Integrated planning

1) People, places and experiences equity lenses[4]

The Social Justice Framework uses three evidence-based lenses to understand the risk of lack of access, exclusion, discrimination and disadvantage - people, places and experiences.

1A) People

To determine who in Greater Bendigo might be more likely to be at risk of lack of access, exclusion, discrimination and disadvantage, the framework applies:

- A First Nations lens, with attention to Dja Dja Wurrung and Taungurung Peoples as the Traditional Owners of the land and to other Aboriginal and Torres Strait Islander people in Greater Bendigo
- A gender lens, with attention to how people of different genders experience gender inequality differently
- An abilities/access and inclusion lens, with attention to the risk of lack of access, exclusion, and discrimination experienced by people with a disability
- A lifespan lens, with attention to children, young people and older people
- A culture lens, with attention to culturally and religiously diverse people, including recently arrived migrants from non-English speaking countries, asylum seekers and refugees, international students, people of diverse faiths and people who experience language barriers
- A class/socio-economic status lens, with attention to poverty and its impacts, people on low incomes and/or in housing stress and intergenerational disadvantage
- A sexual orientation, gender identity and intersex lens, with attention to the risk of discrimination experienced by lesbian, gay, bisexual, trans and gender diverse, queer or intersex people (LGBTQIA+)

NOTE: Not everyone who identifies as belonging to one of these groups will experience disadvantage, discrimination or injustice.

4 The people, place and experiences framework was adopted from the City of Darebin's *Towards equality: equity, inclusion and human rights framework 2019-2029*.

1B) Places

To assess where people might be most at risk of lack of access, exclusion, discrimination and disadvantage, the framework looks at place and location, applying a place-based lens. A place-based approach is important in identifying inequality across Greater Bendigo, identifying neighbourhoods, areas or regions that experience greater levels of socio-economic disadvantage. A place-based approach can:

- Provide an understanding of structural disadvantage within particular neighbourhoods, areas or regions
- Enable residents, groups and stakeholders to engage in a collaborative process, to address issues as they are experienced, within a neighbourhood, area or region, ensuring their strengths, connections and insights inform project, service and infrastructure development
- Assist Council to address inequities in the distribution of resources and infrastructure and other issues as they are experienced within particular neighbourhoods, areas or regions
- Increase social networks and community connections and reduce inequity between and within communities
- Improve residents' daily experiences, shaped by the locations where they live, work or play

A place-based approach is important in assisting Council to prioritise resources (for infrastructure, programs and services) in the context of limited financial resources and rate-capping.

1C) Experiences

The Framework also considers experiences or additional factors that might contribute to exclusion, disadvantage and inequality. Examples are included in the diagram below.

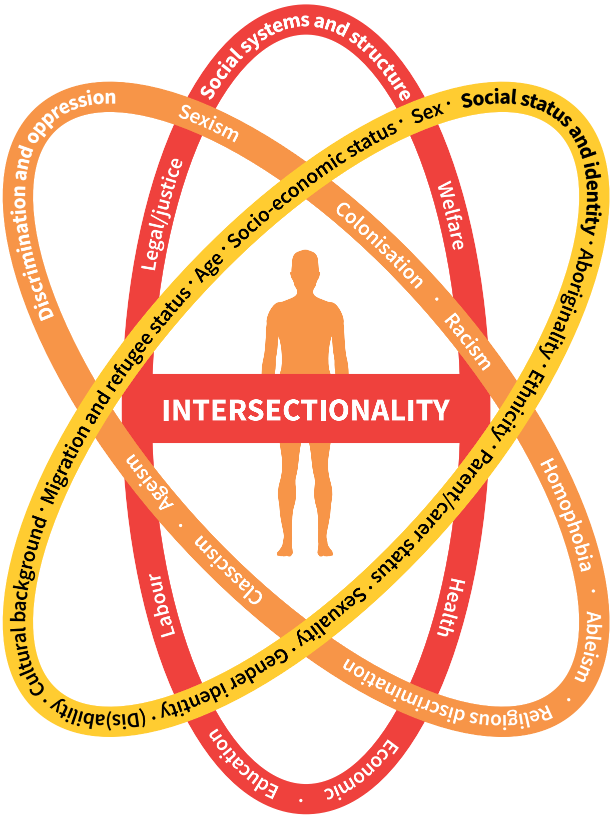

In considering both people and experiences, the idea of intersectionality is invaluable. Intersectionality understands how a person's identity is shaped by multiple realities (such as gender, race/ethnicity, class, age, sexuality, geographic location, age, disability, religion). These factors can help identify multiple contributors to disadvantage and advantage, which impact on people's experiences.

Employment status (job insecurity, unemployment or underemployment)	Housing tenure or housing stress	Homelessness	Poverty or low income	Food insecurity
Migration, including being refugee or seeking asylum	Low level of English proficiency	Low level of literacy	Education level	Access to internet or technology
Family violence and violence against women	Violence or discrimination against LGBTQIA+	Carer role	Pregnancy and breastfeeding	Household composition (sole person, sole-parent families, families with young children)
Trauma and intergenerational trauma	Community safety	Faith	Wellbeing and physical health	Mental health
Social isolation	Out of home care	Engagement with the justice system	Adoption, foster care or institutionalisation	Access to transport

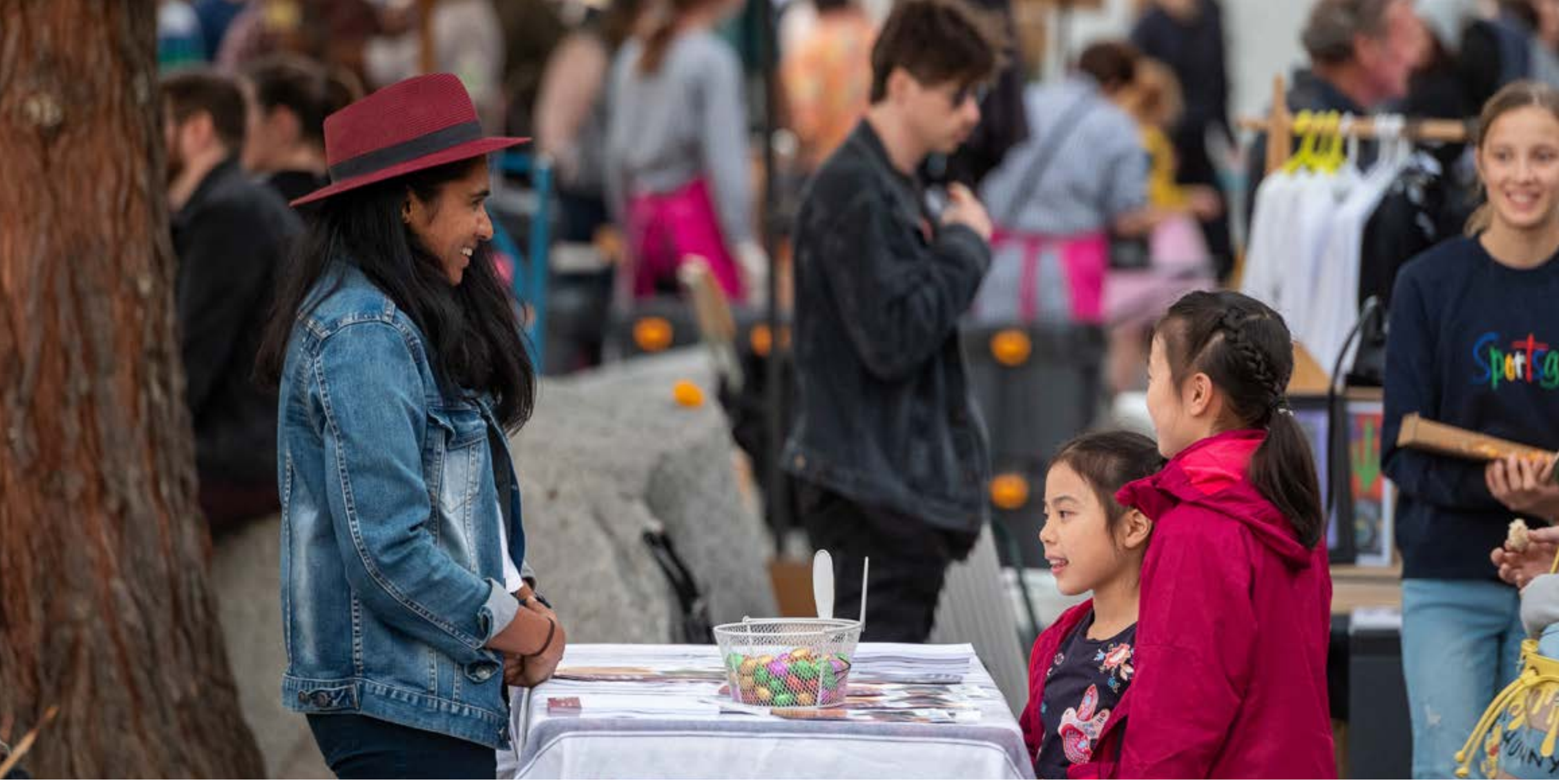

2) Systems change

Systems change is about addressing the barriers that keep the current conditions in place. They can be structural, relational and attitudinal. It identifies that for substantive and long-lasting change, change needs to occur at a variety of levels. This methodology is integral to the Social Justice Framework. It aims to address policies, practices and resource flows to achieve structural change; while simultaneously focussing on relationships, connections and power dynamics to obtain relational change; and also focusses on attitudes, beliefs and culture to achieve attitudinal change. The systems change approach is integral to both **Healthy Greater Bendigo 2021-2025** and the *Gender Equality Action Plan, 2022-2027*.

Six conditions of systems change

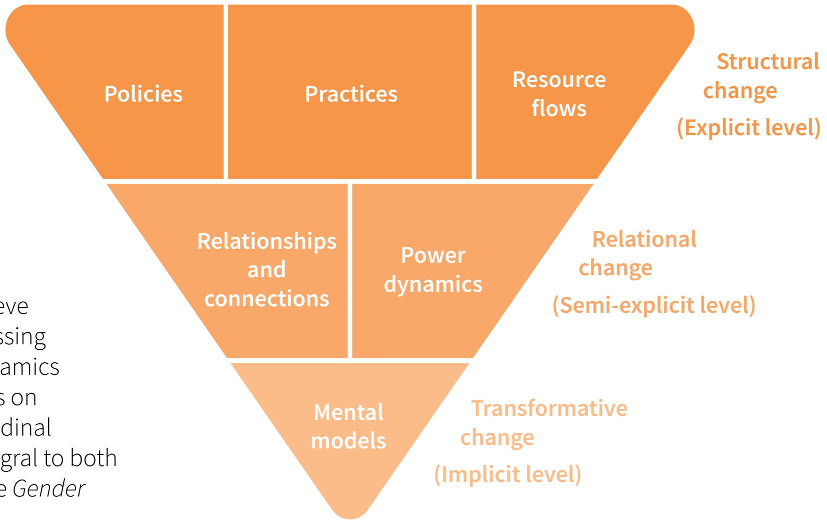

3) Strengths-based

Strengths-based is an approach that looks for opportunities to complement and support existing community and individual strengths and capacities as opposed to focusing on problems, vulnerabilities and deficits. Like asset-based community development, a strengths-based method identifies and mobilises community and individual assets including:

- Local knowledge
- Local culture
- Local resources
- Local skills
- Local processes

A strength-based approach will enable community aspirations, strengths, resources, connections and insights to inform policy, project, service and infrastructure development, while simultaneously supporting capacity building contributing to community and individual resilience and the ability to recover from adversity. A strength-based approach recognises the ways the City can learn a lot from different groups within the community (although they might initially be considered groups at risk) such as the holistic approach to health and wellbeing adopted by Aboriginal and Torres Strait Islander communities and the importance of extended family to both culturally and religiously diverse communities and First Nations people.

4) Integrated planning

Integrated planning is a whole-of-council approach which determines the most appropriate course of action to equitably work toward the Community's vision in alignment with their values. It guides use of a whole of community approach when preparing and delivering plans, policies and projects and incorporates monitoring and evaluation.

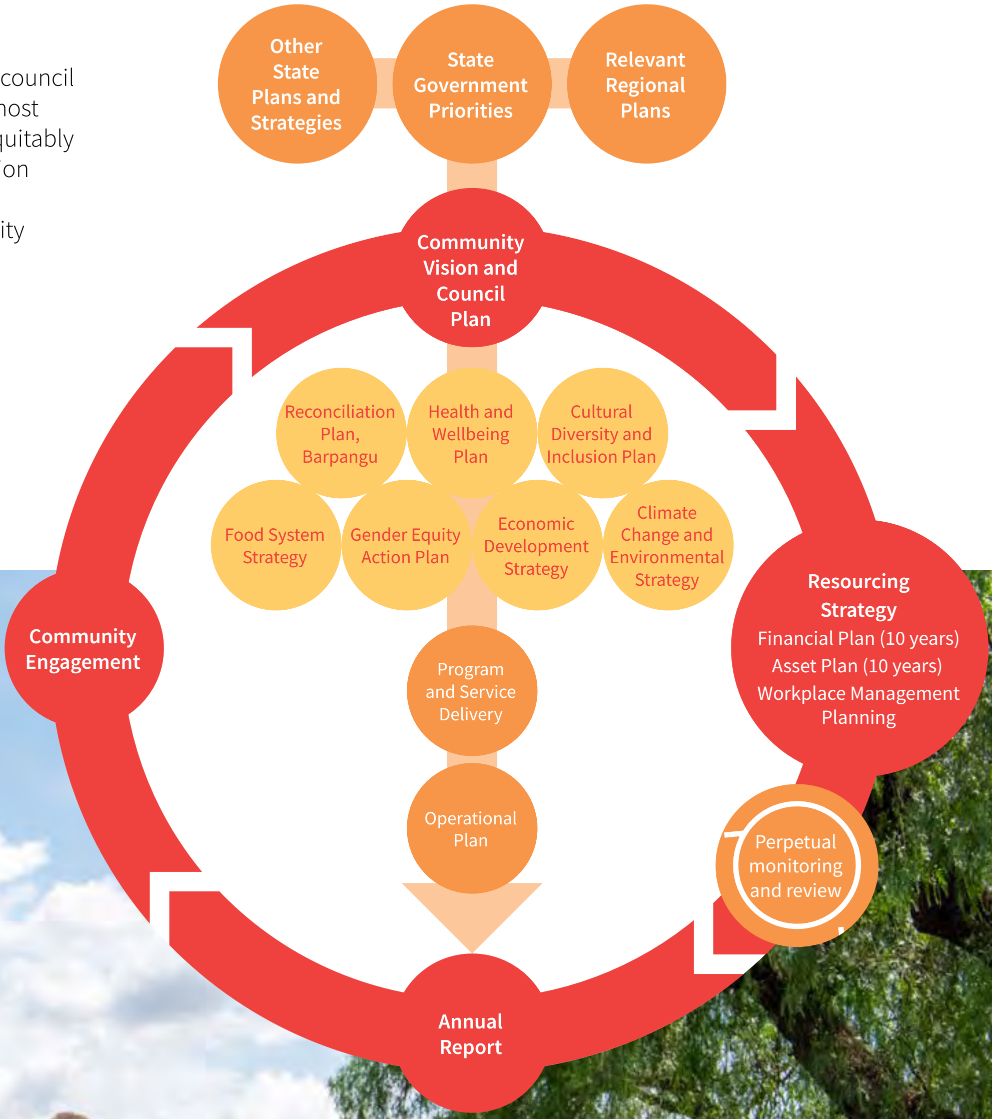

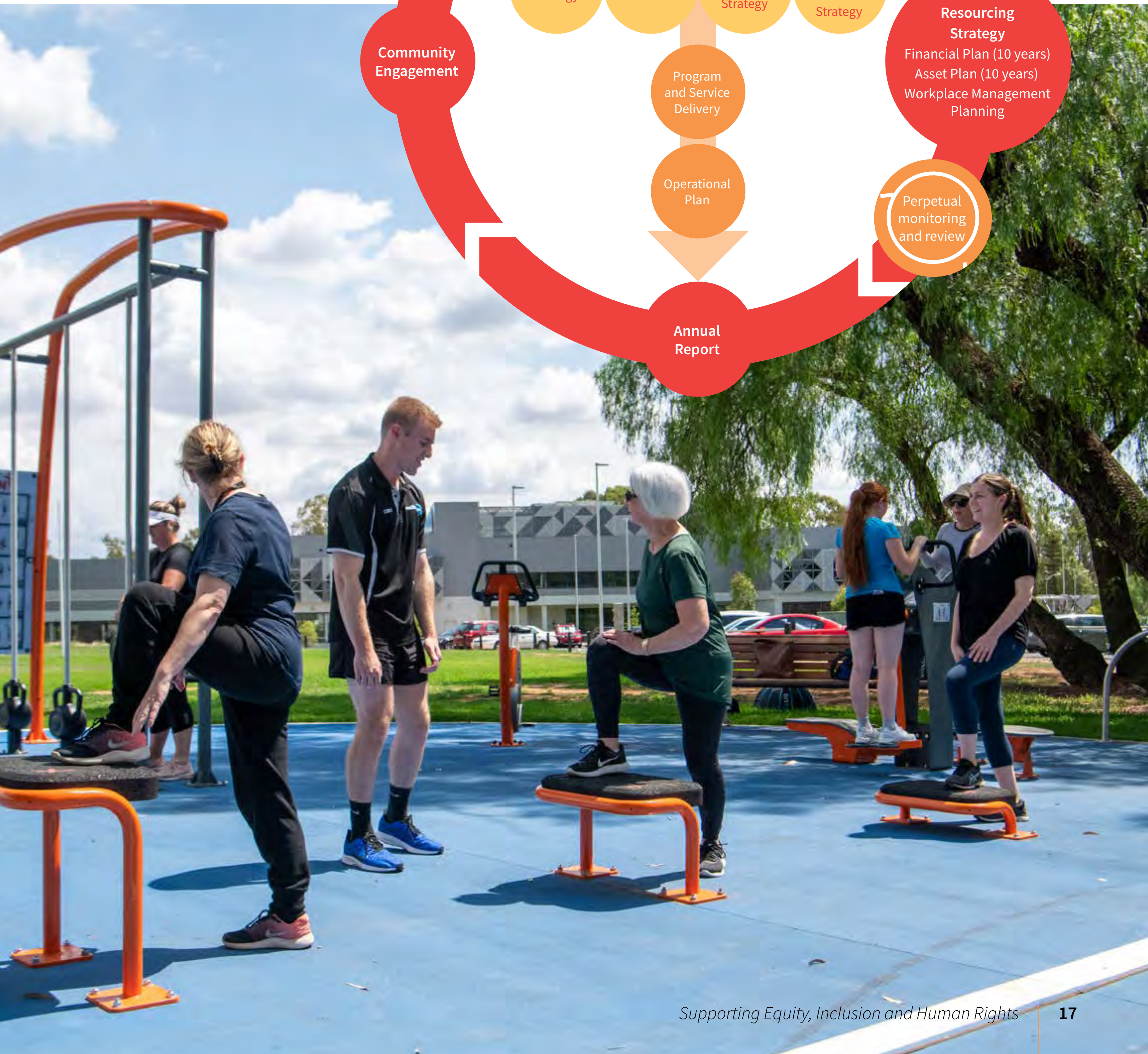

Groups at risk of discrimination or disadvantage

- Aboriginal and Torres Strait Islander
- People experiencing poverty
- Older adults
- Women
- People with disability
- Refugees and people seeking asylum
- Homeless
- Children
- Sole parent families
- People experiencing mental ill-health
- Culturally and religiously diverse
- Faith based communities
- Young people
- LGBTQIA+ community
- People experiencing chronic physical ill-health

Please note: Not everyone who identifies as belonging to one of these groups will experience disadvantage, discrimination or injustice. People who do not identify with one of the groups, may experience disadvantage, discrimination or injustice. People who identify with one or more of these groups, can experience a higher level of discrimination or disadvantage. These groups will change over time.

Barriers to social justice

- Racism
- Social marginalisation
- Food insecurity
- Mental ill-health
- Poor access to education
- Sexism
- Lack of access to services and facilities
- Limited transport options
- Poverty
- Intergenerational disadvantage
- Ageism
- Homophobia, biphobia, transphobia and intersex discrimination
- Limited access to communication technologies
- Low income and unemployment
- Climate change

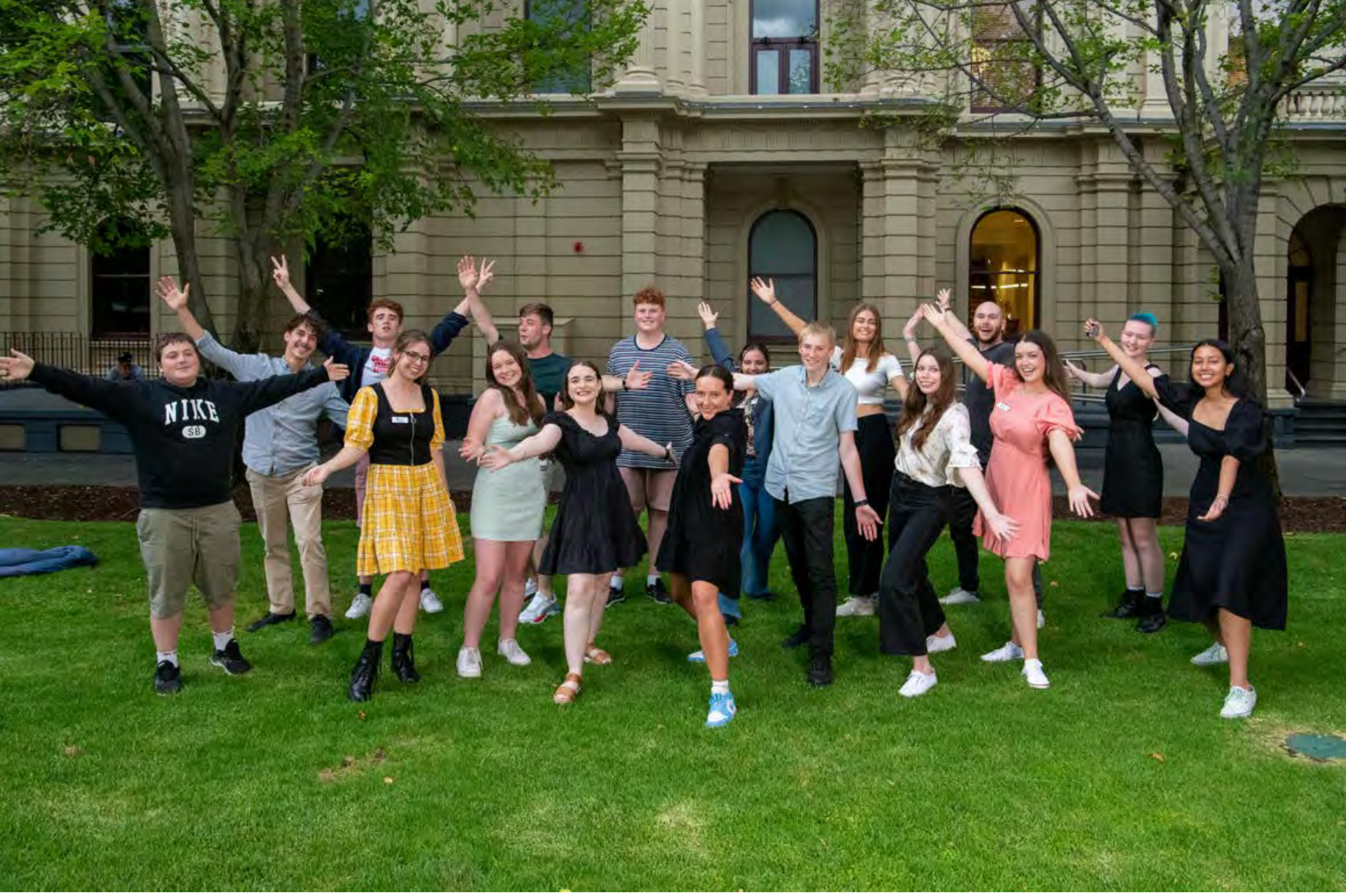

Outcomes

The Social Justice Framework will lead to achieving the following outcomes through all levels in the organisation.

Structural Change

Policies, Programs and Services -
City policies, strategies, plans, programs and services are inclusive and accessible, meeting the rights and requirements of our diverse community.

Practices - The City's governance, leadership, organisational culture and practices display the principles and ethos of equity, access and inclusion and social justice.

Resources - City resources are oriented towards reducing barriers to increase equity and access and inclusion.

Relational Change

Working together - The City supports engagement and partnerships with community members, community groups, agencies, business and tiers of government to create social justice for all.

Power and Participation - The City enables diverse community participation in decision-making.

Advocacy - The City advocates for people in the community who face discrimination, disadvantage and barriers to equity.

Transformative Change

Attitudinal Change - Shifts in City staff and culture mindsets by developing individual, team and organisation adaptive capacity for equity impact assessment, systems thinking and strengths-based approaches.

Training and HR practices - City staff are equipped to support human rights; enable access and inclusion; and enhance relations with diverse communities seeking equity.

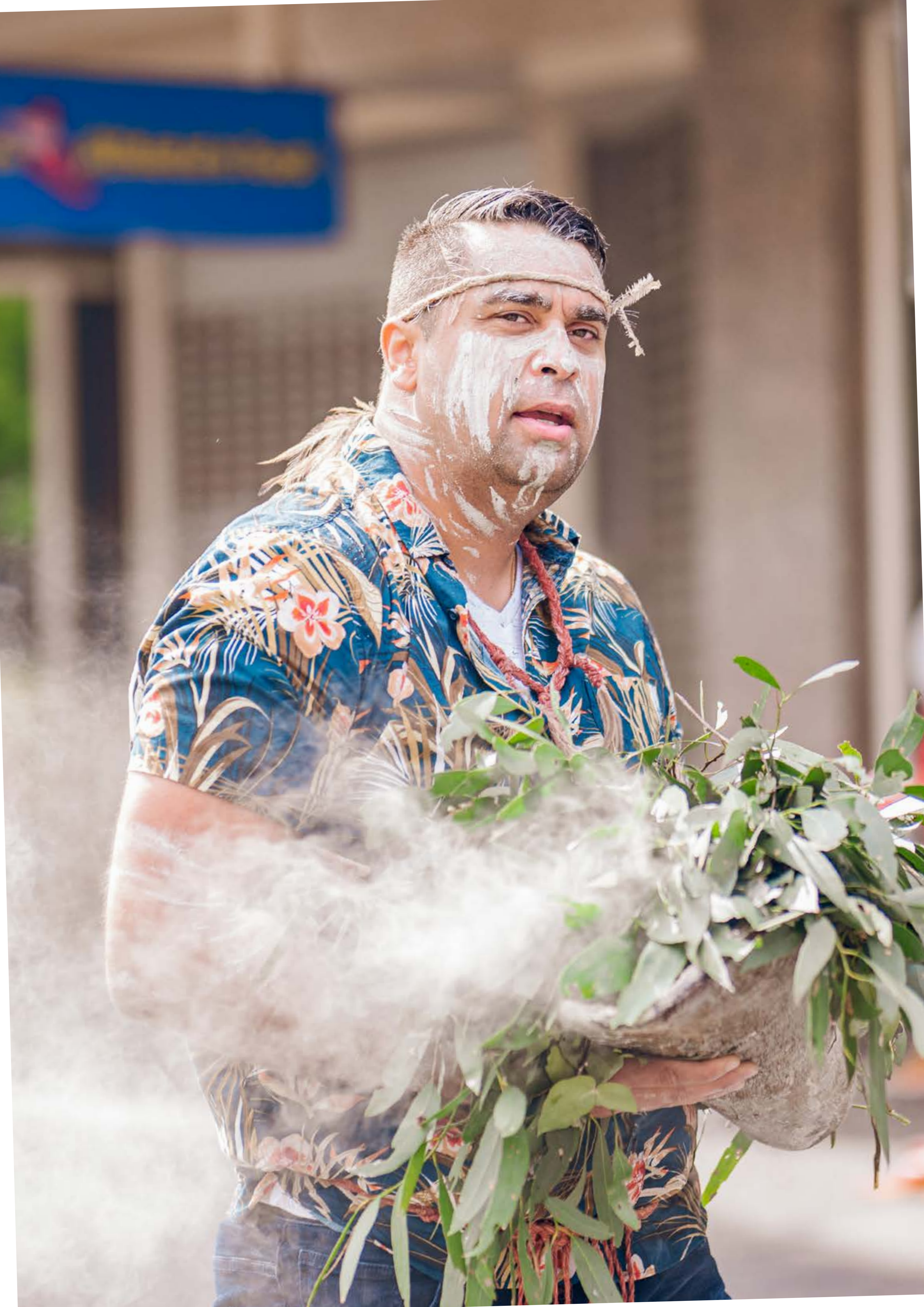

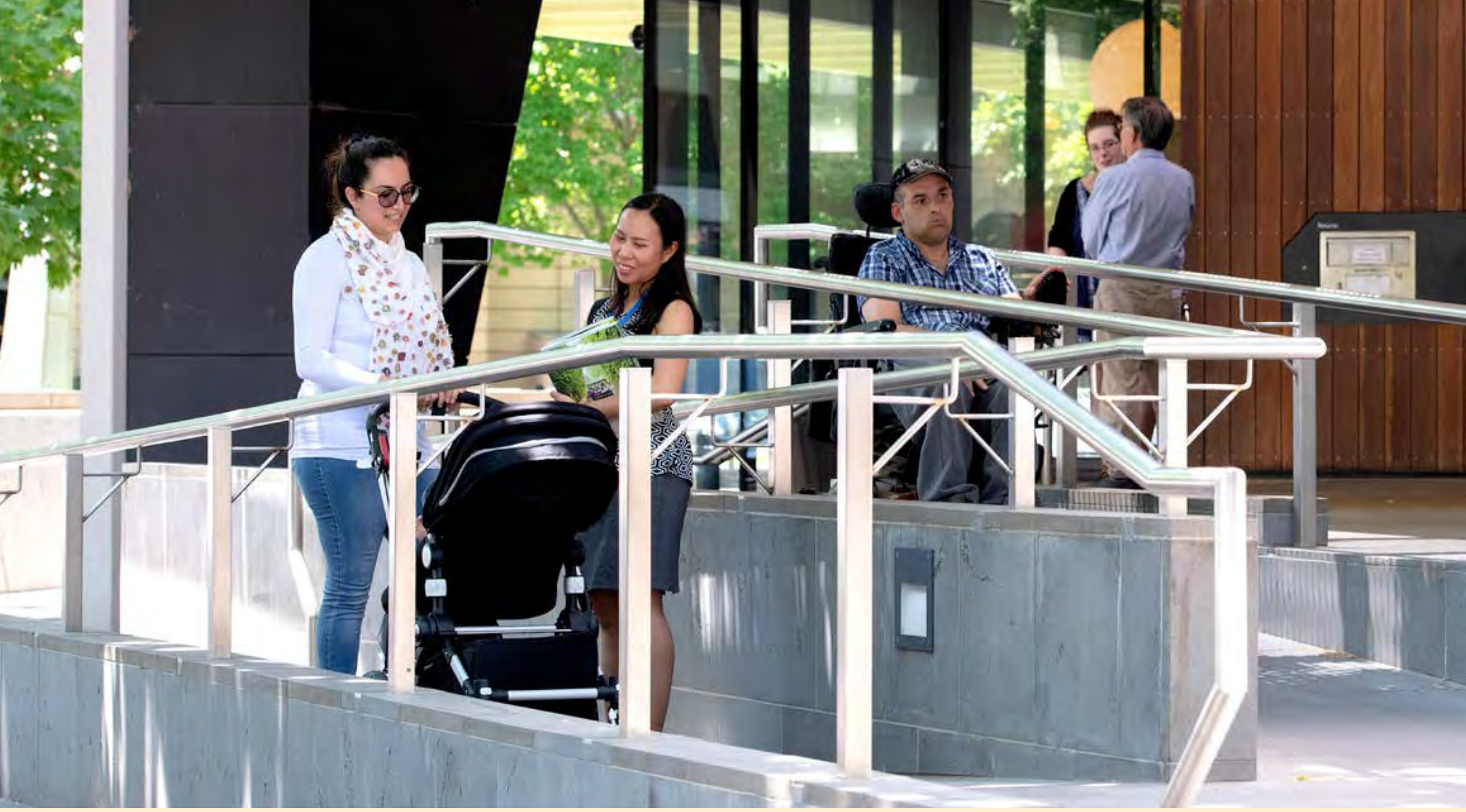

Implementation

The Social Justice Framework will be implemented through the City of Greater Bendigo in an integrated 'whole-of-Council' approach through:

- Policies, strategies and plans
- Services and programs
- Advocacy
- Partnerships
- Resources
- Governance and organisational culture practices
- Staff awareness and training
- Equity Impact Assessments (EIA)

A Social Justice Framework Action Plan (as per recommendation 2 of the Human Rights Charter Evaluation, 2021, p.3) with a Social Justice Report Card will ensure implementation occurs effectively. The Equity and Disadvantage Working Group, with representation across the organisation, will assume responsibility for developing and overseeing the Social Justice Framework Action Plan and monitoring the implementation of actions. In developing actions to develop tools and build staff capability to apply social justice principles, the Working Group will draw on the learnings from the Human Rights Charter Evaluation, 2021, which identified City's staff's commitment to social justice principles but the challenge of application. The Working Group will also develop strategies to bring the voices of people with lived experience to the City's social justice work over the life of the framework.

Equity Impact Assessments (EIA), which emerged from the City's pilot Gender Impact Assessments (GIA), required under the *Gender Equality Act 2020*, will ensure that all City policies, programs and services which have a direct and significant impact on the public consider equity (while also helping meet the different needs of different genders, eliminating barriers to gender equality; and actively promoting gender equality).

The Social Justice Framework will support Council to work towards equity, access and inclusion, and human rights being a priority in all of Council's business. The Social Justice Framework will be championed across the organisation by:

- The Executive Management Team (Chief Executive Officer and Directors)
- The Organisation Leadership Team
- Members of the Equity and Disadvantage Working Group
- Members of the Gender Equity Working Group
- Equity Champions (staff from all levels and different units)

These Equity Champions will enable the City of Greater Bendigo to promote the Social Justice Framework throughout the organisation and the community. The Equity Champions would align with, and build on, the work of Gender Equity Champions, which has a strong intersectional focus and also connect with other Champions (such as Reconciliation Champions).

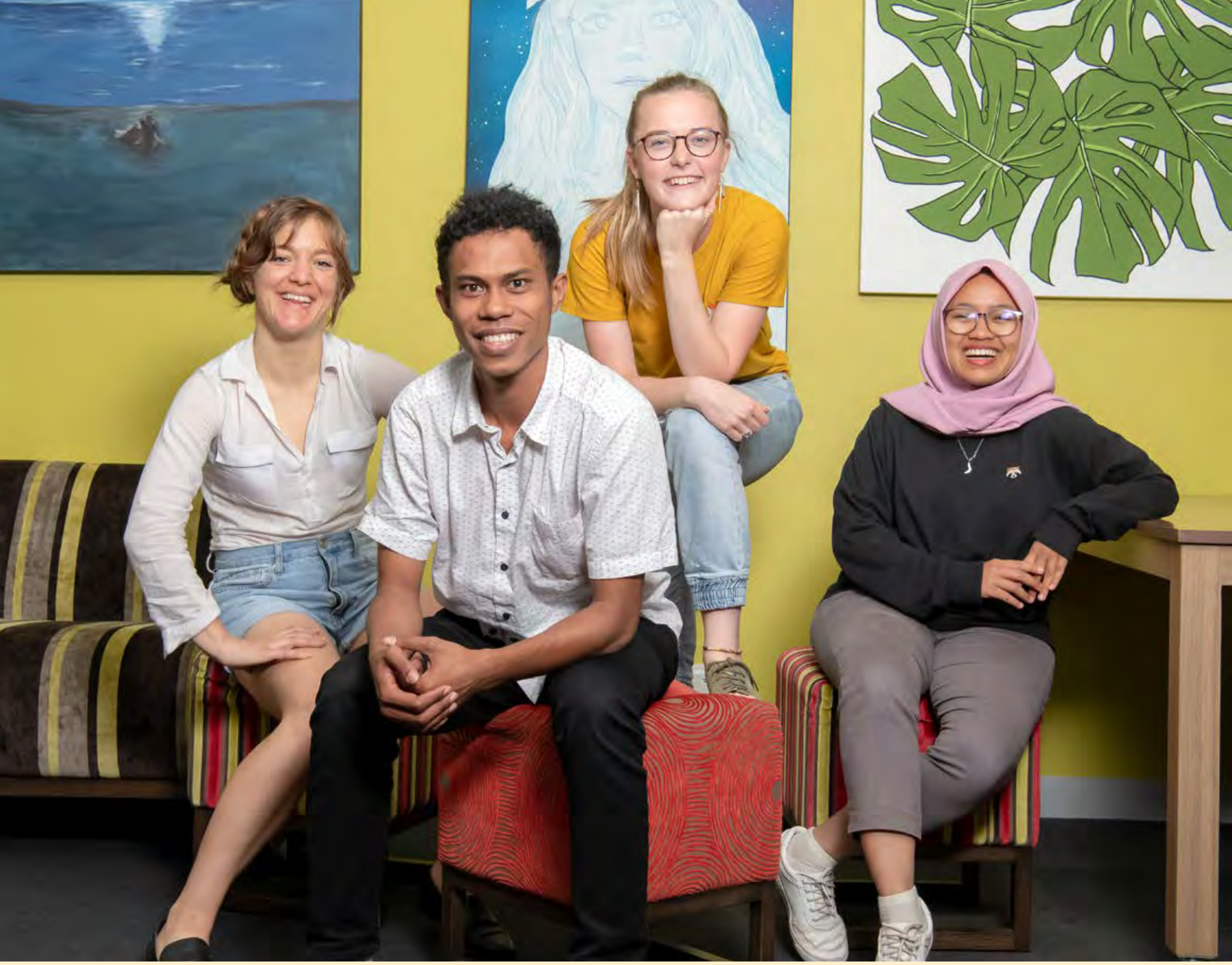

Evaluation and monitoring

The evaluation of the Social Justice Framework will adopt the measurement, evaluation and learning (MEL) approach as a way of providing detail of strengths and areas for improvement. This process will help identify the impact of the Social Justice Framework and Equity Impact Assessment Tool; develop best practice case studies; and identify areas for development. The monitoring and evaluation measurements will be used to determine whether the City is on track to achieving the Social Justice Framework Action Plan objectives and if actions need updating to respond to changes in Government policy or legislation and new issues or opportunities.

A Social Justice Framework Action Plan will provide a structure for monitoring progress and outcomes across the whole organisation. The Social Justice Action Plan will be monitored and reviewed by the Equity and Disadvantage Working Group.

Best practice community engagement will enable community/stakeholder input on existing and emerging issues related to social justice, equity, access and inclusion and human rights which need to be included in other Council plans or the Social Justice Framework Action Plan. This community/stakeholder feedback will enable ongoing engagement to ensure the Social Justice Framework and Action Plan remains relevant to the community.

Changes in social justice and equity issues may occur over a longer period of time. Although the impact of the Social Justice Action Plan will be measured regularly, change may be incremental in the short term, however when viewed over the long term it is expected to be substantial and lasting.

Appendix 1: Policy context

The Social Justice Framework has been developed in the context of the Bendigo Human Rights Charter evaluation (2021) and City of Greater Bendigo policies and plans but also from State and Federal government legislative requirements and from Australia's ¬– and Greater Bendigo's - commitment to United Nations declarations and agendas. State and Federal legislation recognises that some people are at increased risk of discrimination or disadvantage. The protections in the legislation require Council to have an understanding and knowledge of which people, or groups of people, in the Greater Bendigo community may be more vulnerable to discrimination, marginalisation and exclusion, including systemic and structural discrimination.

Local

The Social Justice Framework builds on earlier and existing City of Greater Bendigo policies and plans, particularly the principles, values, outcomes, goals and objectives in:

- **City of Greater Bendigo Human Rights Charter** (2014)
- **Greater Bendigo Community Vision and Values** (2021-2030)
- **Council Plan 2021-2025 *Mir wimbul***
- **Healthy Greater Bendigo: Municipal Public Health and Wellbeing Plan 2021-2025**
- ***Barpangu* Build Together: City of Greater Bendigo Reconciliation Plan, 2021-2025**
- **Cultural Diversity and Inclusion Plan, 2021-2025**
- **Equity for All (E4A) Gender Equity Action Plan, 2021-2025**
- **Affordable Housing Action Plan, 2021**
- **A Stronger Greater Bendigo 2030 (Economic Development Strategy)**
- **Connecting Greater Bendigo: Integrated Transport and Land Use Strategy (ITLUS), 2015**
- **Climate Change and Environmental Strategy 2021-2026**
- **Community Engagement Policy, 2020**

State

In Victoria, the principles of social justice and human rights are protected by law as described in the following legislation.

Victorian Local Government Act 2020 - Section 8 of Victorian *Local Government Act 2020* states that Council must act "for the benefit and wellbeing of the municipal community", by:

- ensuring that "services should be provided in an equitable manner and be responsive to the diverse needs of the municipal community" (section 106 (2) a and b)
- achieving the best outcomes for the community, including future generations (section 9 (2) b)
- engaging with the community (sections 9 (2) d and 56) and being transparent, and by providing understandable and accessible information (section 58 a and c), as well as collaborating with others (sections 9 (2) f)
- promoting economic, social and environmental sustainability, including planning for climate change (section 9 (2) c)

Victorian Charter of Human Rights and Responsibilities Act 2006 - Council is bound by the Victorian *Charter of Human Rights and Responsibilities Act 2006* (the Charter). The Charter sets out the basic rights, freedoms and responsibilities of all people in Victoria and requires:

- local government to take human rights into consideration when making laws, setting policies and providing services
- all public authorities to act in a way that is compatible with human rights

Under the Charter, local councils have an obligation to respect, and act compatibly with, human rights in the way they go about their work and deliver their services. They have a responsibility to demonstrate how all of their decisions align with the Charter and how they're not only upholding but also promoting human rights.

Victorian Gender Equality Act 2020 - In accordance with the *Gender Equality Act 2020*, Council must:

- improve and promote gender equality, both as a workplace and through the policies, programs and services developed for the Greater Bendigo community
- support better outcomes for people of all genders
- address gender inequality in Greater Bendigo and publicly report on our progress

The *Gender Equality Act* adopts an intersectional approach, recognising that for many Victorians, "gender inequality may be compounded by other forms of disadvantage or discrimination that a person may experience due to other characteristics, such as race, Aboriginality, religion, ethnicity, disability, age, sexual orientation, gender identity." The *Gender Equality Act* requires organisations to consider these when developing strategies and measures to promote gender equality.

Multicultural Victoria Act 2011 - Recognises and values the cultural, religious, racial and linguistic diversity of the people of Victoria.

Victorian Equal Opportunity Act 2010 - As an employer, a service provider and in all of its operations, Council is bound by the *Equal Opportunity Act 2010*, which prohibits discrimination. Under the Act, Council must:

- promote the elimination of discrimination, sexual harassment and victimisation and work towards the gradual realisation of equality.
- protect people from discrimination and harassment in areas of public life including local government, workplaces and places that provide services.

The *Equal Opportunity Act 2010* aims for greater equality and the prevention of discrimination by placing a "positive duty" on local government to take proactive, reasonable and proportionate measures to eliminate discrimination, sexual harassment and victimisation. This includes being an equal opportunity employer, and reviewing Council and City policies, practices, services, programs, places and spaces to prevent discrimination.

Victorian Family Violence Protection Act 2008

Victorian Public Health and Wellbeing Act 2008 - Local government has a role in supporting community members to achieve optimal health and wellbeing (section 24).

Victorian Disability Act 2006

Victorian Racial and Religious Tolerance Act 2001

- Promotes racial and religious tolerance in Victoria
- Emphasises "the democratic value of the equal participation of all citizens in society"

The Social Justice Framework was also informed by **Pride in our future: Victoria's LGBTIQ+ strategy 2022-32**.

Federal

Under federal law, people are protected from discrimination on the grounds of race, sex, disability, age, sexual orientation, gender identity and intersex status. Legislation includes:

- *Australian Human Rights Commission Act 1986* (Cth)
- *Age Discrimination Act 2004* (Cth)
- *Disability Discrimination Act 1992* (Cth)
- *Sex Discrimination Act 1984* (Cth)
- *Racial Discrimination Act 1975* (Cth)
- *Fair Work Act 2009* (Cth)

International

Australia is signatory to several United Nations Declarations and Agendas related to human rights and social justice, including:

- *The Universal Declaration of Human Rights* (1948)
- *The United Nations Convention on the Rights of the Child* (1990)
- *The United Nations Declaration on the Rights of Indigenous Peoples* (2007)
- *The United Nations Convention on the Rights of Persons with Disabilities* (2007)
- *The United Nations 2030 Agenda for Sustainable Development* (2015)

The *Agenda for Sustainable Development* pledges "No one will be left behind" and the goals were developed to provide a roadmap for all countries to work toward a better world for current and future generations. All stakeholders, including local government, are expected to contribute to the realisation of these goals.

Greater Bendigo committed to address the 17 goals in the **Council Plan 2021-2025 *Mir wimbul***. The goals "recognize that ending poverty and other deprivations must go hand-in-hand with strategies that improve health and education, reduce inequality, and spur economic growth – all while tackling climate change".

The goals most relevant to the Social Justice Framework are:

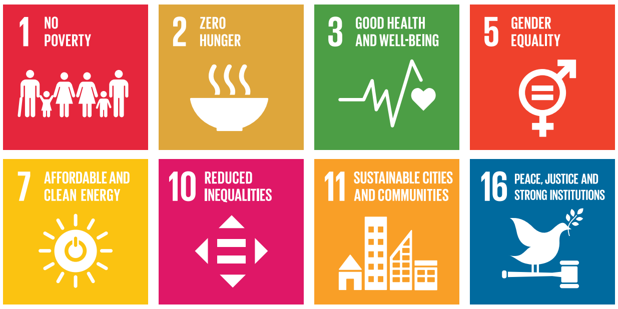

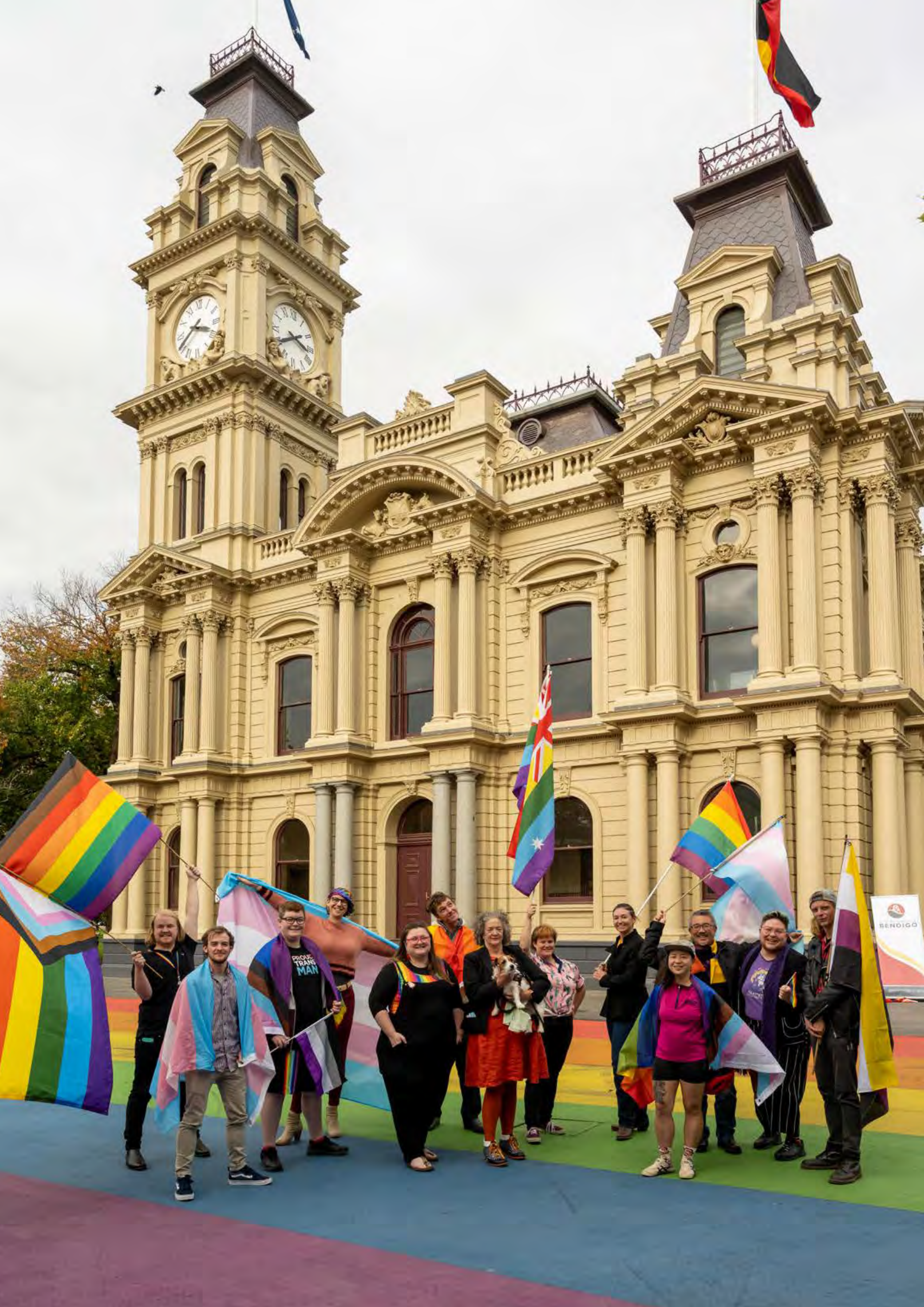
BENDIGO

CITY OF GREATER
BENDIGO

Address:
15 Hopetoun Street, Bendigo
125 High Street, Heathcote

Phone:
1300 002 642
National Relay Service: 133 677
then quote 03 5434 6000

Email:
requests@bendigo.vic.gov.au

Website:
www.bendigo.vic.gov.au

Is an Equity Impact Assessment Required?

This 5-10minute survey will help you determine if an Equity Impact Assessment (EIA) is required for your initiative. Equity Impact Assessments incorporate Gender Impact Assessments which are a legislative requirement of the Gender Equality Act 2020. The results are automatically generated, and a copy of the results will also be sent to you via email for your records.

1. Full Name *

First Name

Last Name

Email *

2. Directorate - Unit *

Office to the CEO

3. What is the name of the policy, program or service (or initiative) being assessed? *

4. According to the table below, is the initiative defined as a policy, program or service?

Policy

Policy	Program	Service
Formal or informal principle statements, standards or rules.	An initiative, project or set of projects with a defined duration and set of deliverables.	The supply of work or facility that meets a public need and is delivered by the City, its staff or an organisation on behalf of the City.
Documents containing policy in the title	Project Plan	Service Profiles
Local laws	Business Plan	Service Plan
Legal policy proposals	Business Case	
Strategies, Frameworks or Plans	Budget Bid	

5. Is the policy, program or service in development or review? (Is it being developed for the first time or is there a formal review process assigned to it which due date is upcoming?)

Development

6. Is the community the primary focus of the policy, program or service? (As opposed to the City, its employees or other organisations?) NB: For policies, a helpful litmus test is whether it is a Council Policy or Organisational Policy.

Yes

7. Does the policy, program or service (tick all that apply)

- [ ] a) reach a large proportion of the community
- [ ] b) have a large impact on a small proportion of the community
- [ ] c) target community members who experience higher levels of disadvantage or have specialised needs?

(Consider the following identity factors: Aboriginality, age, disability, ethnicity, gender identity, race, religion and sexual orientation).

8. How significantly does the policy, program or service impact the City of Greater Bendigo's ability to achieve the strategic outcomes in the Public Health and Wellbeing Plan?

#1 Healthy and Well

The Greater Bendigo community has good physical and mental health

Little to no impact on Council's ability to achieve this outcome

This outcome is linked to Council Plan Outcome #2 Healthy, liveable spaces and places.

#2: Safe and Secure

The Greater Bendigo community lives free from violence and abuse, is protected from environmental risks and is supported to live affordably

Little to no impact on Council's ability to achieve this outcome

This outcome is linked to Council Plan Outcome #7 A safe, welcoming and fair community.

#3: Able to Participate

The Greater Bendigo community has the capability to participate and contribute to the local economy and community life.

Little to no impact on Council's ability to achieve this outcome

This outcome is linked to Council Plan Outcome #3 Strong, inclusive and sustainable economy, Outcome #6 A vibrant, creative community and Outcome #7 A safe, welcoming and fair community.

#4: Connected to Culture and Community

The Greater Bendigo community is socially engaged and inclusive, and is a place where people can safely identify and connect with their culture and identity

Little to no impact on Council's ability to achieve this outcome

This outcome is linked to Council Plan Outcome #4 Aboriginal reconciliation and Outcome #7 A safe, welcoming and fair community.

#5: Liveable

The Greater Bendigo community is resilient to a changing climate, and has access to the natural environment and quality public space for enhanced health and wellbeing.

Little to no impact on Council's ability to achieve this outcome

This outcome is linked to Council Plan Outcome #5 A climate-resilient built and natural environment.

Total Score

0

Submit Form

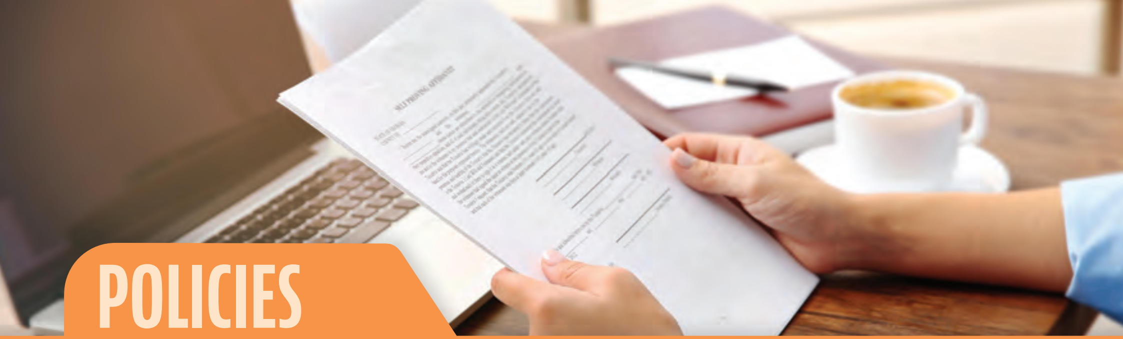

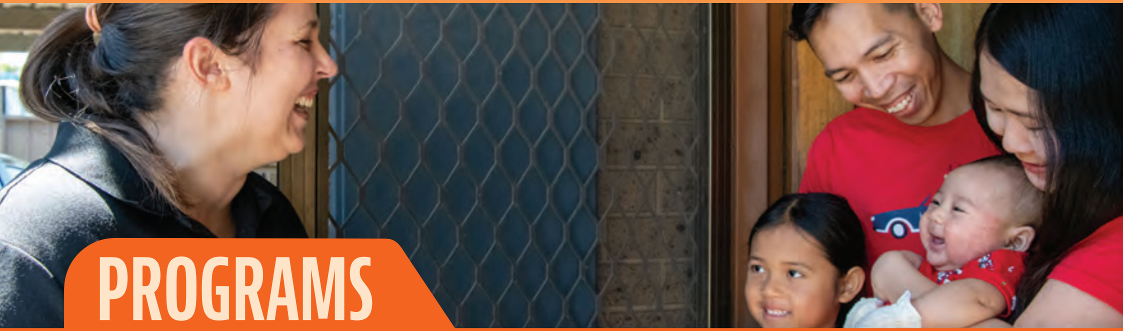

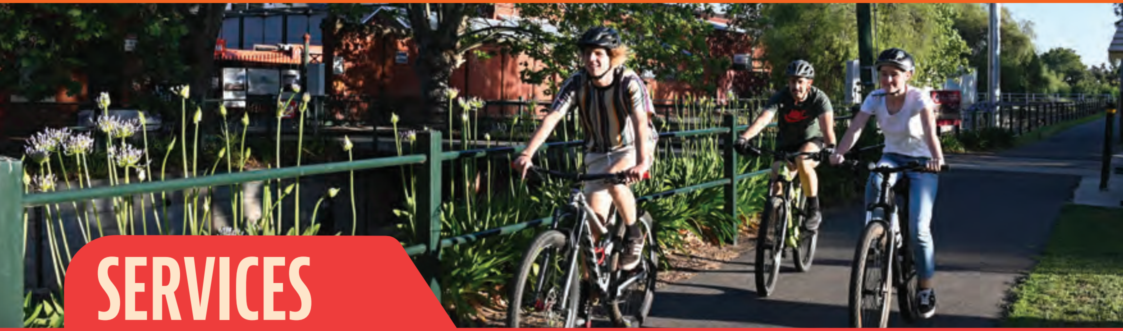

Equity Impact Assessment Guide

How to conduct an Equity Impact Assessment (EIA)

CITY OF GREATER
BENDIGO

Contents

The Gender Equality Act 2020 4

The Commissioner and Commission for Gender Equality in the Public Sector 4

The City of Greater Bendigo: A defined entity of the Act 4

What is a Gender Impact Assessment? 5

What is an Equity Impact Assessment? 6

How to determine if an EIA is legislatively required 7

The 'Is an EIA required?' survey 7

A) What is a policy, program or service? 8

B) What is a direct and significant impact? 9

C) What is a development or review process 10

How to conduct an Equity Impact Assessment 11

The four steps of an EIA 11

Selecting an EIA Template 12

At what stage of the development or review process should an EIA be conducted? 12

Undertaking a facilitator-led or self-led EIA 13

How long does the EIA process take? 14

Using DocAssembler to complete the EIA template 14

EIA Executive Summary 14

EIA Recommendations 15

Implementing an EIA Recommendation 15

EIA Champions 16

Who is an EIA Champion at the City? 16

How to become an EIA Champion 16

EIA Champion Guide 16

Monitoring, Learning and Evaluation Plan 18

EIA Case Studies 18

Frequently used terminology 19

The Gender Equality Act 2020

The *Gender Equality Act 2020* was introduced into Victorian legislation in March 2020 following recommendations by *Safe and Strong, Victoria's Gender Equality Strategy* and the *Royal Commission into Family Violence* that the promotion of gender equality was required in order to reduce levels of family violence and all forms of violence against women. The Act promotes gender equality by requiring the Victorian public sector to make meaningful progress towards gender equality in the workplace and in the community.

The Commissioner and Commission for Gender Equality in the Public Sector

The Commissioner for Gender Equality in the Public Sector (the Commissioner) oversees the implementation of the Act and plays a key leadership role in promoting gender equality in the Victorian community and workplaces – including Councils. The Commissioner is supported by the Commission for Gender Equality in the Public Sector (the Commission).

The City of Greater Bendigo: A defined entity under the Act

The Act applies to organisations within the Victorian public sector that have 50 or more employees, including local councils, which makes the City of Greater Bendigo a defined entity under the Act. Defined entities have legal obligations to promote gender equality in their own workplaces through a gender audit and action plan; and consider and promote gender equality in policy, program and service delivery through gender impact assessments.

What is a Gender Impact Assessment?

Gender impact assessments are designed to help organisations think critically about how policies, programs and services will meet the different needs of women, men and gender diverse people. The purpose of gender impact assessments is to create better and fairer outcomes and make sure all people have equitable access to opportunities and resources.

To be legislatively compliant with the Act, a gender impact assessment must:

- Assess the effects that the policy, program or service may have on people of different genders
- Explain how the policy, program or service will be changed to better support Victorians of all genders and promote gender equality
- Where practical, apply an intersectional approach to consider how gender inequality can be compounded by disadvantage or discrimination that a person may experience on the basis of other factors such as age, disability or ethnicity

What is an Equity Impact Assessment?

The City conducts Equity Impact Assessments, which incorporate Gender Impact Assessments, to consider how policies, programs and services affect different genders and diverse groups in different ways and seek to introduce changes that will help create a fair and inclusive community. The four-step process helps staff identify issues and challenge assumptions that are creating inequities in policy, program and service delivery, and through a process of research and targeted consultation, develop meaningful options for change that will increase equitable outcomes in policy, program or service delivery.

EIAs put the City's **Social Justice Framework 2022-2032** into action by encouraging staff to adopt three critical lenses when analysing the impact of a policy, program or service on different genders and diverse groups; a people lens, a place-based lens and an experience lens. The people lens helps staff determine who in Greater Bendigo is at risk of lack of access, exclusion, discrimination and disadvantage. The place-based lens helps staff explore the impact of location (neighbourhoods, areas or regions) on the needs of diverse people. The experience lens helps staff consider experiences or additional factors that might contribute to exclusion, disadvantage and inequality.

EIAs help staff explore the concept of intersectionality which seeks to identify who might be experiencing multiple forms of discrimination or disadvantage and unpack how these forms of discrimination compound and increase barriers to access, inclusion and equality in policy, program and service delivery.

How to determine if an EIA is legislatively required

To determine if an initiative at the City legislatively requires an Equity Impact Assessment (incorporating Gender Impact Assessments), staff will need to determine if it meets all three criteria:

A) is defined as a policy, program or service under the Act,

B) has a direct and significant impact on the public and

C) is in development or review.

The 'Is an EIA required?' survey

The City uses an 'Is an EIA required?' smart survey to support staff in determining whether a policy, program or service meets the legislative criteria for requiring an Equity Impact Assessment.

The smart survey promotes organisational consistency in applying the legislative criteria by prompting a series of questions that independently determine whether an EIA is legislatively required or not. A written confirmation of the survey outcome will be sent to the staff member's email address so it can be recorded and reported against.

A) What is a policy, program or service?

The Commission provides the following guidance on what constitutes a policy, program or service under the Act.

Policy	Program	Service
Formal or informal principle statements, standards or rules.	An initiative, project or set of projects with a defined duration and set of deliverables.	The supply of work or facility that meets a public need and is delivered by the City, its staff or an organisation on behalf of the City (view full list of **City services**).
Any document containing policy in the title	Project Plan	Service Profiles
Local laws	Business Plan	Service Plan
Legal policy proposals	Business Case	
Strategies, Frameworks or Plans	Budget Bid	

B) What is a direct and significant impact?

Staff must assess whether the policy, program or service as defined by the Act has a direct and significant impact on the public.

To support organisational consistency in applying this criterion, staff are requested to take the **'Is an EIA required?' smart survey**. The survey will determine whether a policy, program or service has a direct and significant impact based on the target audience, its scope and intended reach, and alignment with the Public Health and Wellbeing Plan.

DIRECT:

1. Is the community the primary focus of the policy, program or service? (As opposed to the City, its employees or other organisations?) *NB: For policies, a helpful litmus test is whether it is a Council Policy or Organisational Policy.*

SIGNIFICANT:

2. Does the policy, program or service (tick all that apply)

- ☐ reach a large proportion of the community
- ☐ have a large impact on a small proportion of the community
- ☐ target community members who experience higher levels of disadvantage or have specialised needs? *Consider the following identity factors: Aboriginality, age, disability, ethnicity, gender identity, race, religion and sexual orientation.*

3. How significantly does the policy, program or service impact the City of Greater Bendigo's ability to achieve the strategic outcomes in the Public Health and Wellbeing Plan? (Healthy and Well, Safe and Secure, Able to participate, Connected to Culture and Community, Liveable)

C) What is a development or review process?

A policy, program or service is considered in development if it is not yet existing and is being developed for the first time. It is considered in review if there is a formal review process to which the policy, program or service is required to undergo at a specific time, and the due date for review is upcoming.

If a policy, program or service as defined by the Act has a direct and significant impact on the public but is not in development or review, it does not legislatively require an Equity Impact Assessment.

How to conduct an Equity Impact Assessment

The four steps of an EIA

The Equity Impact Assessment is a four-step process which staff can undertake with the support of a facilitator (EIA Champion) or independently through a self-led approach, with approval from a Gender Equity Officer. The four steps are:

Step 1: Define the issue and challenge assumptions*

This step helps staff consider how the policy, program or service might affect different genders and diverse groups. This step will encourage staff to adopt a people, place and experience lens and analyse the policy, program or service using an intersectional approach. Staff will decide on a specific issue which will become the EIA focus, to which further research and analysis will be applied.

* *If the EIA is facilitator-led by an EIA Champion, Step 1 will be conducted in a workshop setting.*

Step 2: Understand the context

Staff undertake basic research on their EIA focus, looking at internal data the City already has, conducting desktop research and targeted consultation, including benchmarking with similar organisations if relevant. This research will be used to inform option/s development that will occur in Step 3.

Step 3: Propose options for change

Staff will develop 1-2 options of recommended changes that they believe will remove or reduce barriers to equality in policy, program or service delivery. Staff will assess the costs and benefits of each option prior to making a recommendation for implementation.

Step 4: Make a recommendation

Staff will use the information and analysis conducted in Steps 1-3 to select an option for recommendation that will be reviewed by an EIA Champion and assigned to the policy, program or service lead for implementation. This EIA recommendation will also be included in the biennial progress report produced for the Commissioner for Gender Equity.

Selecting an EIA Template

Staff utilise an EIA template to guide them through the Equity Impact Assessment process. There are three variations of the EIA template to suit whether the EIA is being conducted on a policy, program, service, plan, framework or strategy:

Template A: Policy

Template B: Program or Service

Template C: Plan, Framework or Strategy

At what stage of the development or review process should an EIA be conducted?

Equity Impact Assessments should be conducted at the outset of a policy, program or service development or review process. The EIA recommendation may impact how consultation is done or who is consulted so it is important an EIA is conducted before these processes begin. Doing an EIA right at the start also allows enough time for the EIA recommendation to be implemented before the development or review process is complete and due for approval/endorsement by the Executive Management Team (EMT) or Council.

Undertaking a facilitator-led or self-led EIA

Facilitator-led

EIA Champions facilitate workshops for Step 1 and schedule a midway check-in with the policy, program or service lead. They will also conduct a review of the EIA template once the draft is complete to advise any necessary amendments. Following this, EIA Champions will provide final authorisation that the EIA has been completed to a satisfactory standard and that the EIA recommendation is supported by relevant research, option development and analysis.

Staff are required to take a facilitator-led approach if:

- They have never participated in an EIA before
- The EIA is for a parent policy, plan, strategy or framework that informs other policies, programs or services

Self-led

Staff members independently utilise the EIA Guide and EIA template to guide them through the EIA process from start to finish. Approval must be sought from a Gender Equity (GE) Officer before taking this approach. An EIA Champion will conduct a review of the EIA template to advise any necessary amendments. Following this, the EIA Champion will provide final authorisation that the EIA has been completed to a satisfactory standard and that the EIA recommendation is supported by relevant research, option development and analysis.

Staff are permitted to undertake the self-led option if:

- They have participated in a previous EIA as the policy, program or service lead
- The EIA is not a parent policy, plan, strategy or framework that informs other policies, programs or services

How long does the EIA process take?

Staff are advised to assign up to half a working day per step (4 steps in total) and allow additional time for making recommended amendments once it is reviewed by an EIA Champion.

Using DocAssembler to complete the EIA template

EIA Templates are to be completed on **DocAssembler** which can be accessed on the Sharepoint homepage.

An EIA workflow approval process will include:

- Policy, program or service lead (owner)
- EIA Champion (1st approver)
- People Manager (2nd approver)
- Gender Equity Officer (final approver)
- EIA templates will be published on ECM through DocAssembler by the final approver

EIA Executive Summary

The EIA Executive Summary provides a succinct overview of the Equity Impact Assessment and is intended to be attached to relevant EMT reports or Council briefings/meetings in lieu of the full EIA template. This Executive Summary also supports closing the loop with EIA workshop participants or community members with whom staff wish to communicate the outcomes of the EIA. Executive Summaries will also be used to inform 1-pager case studies hosted on the EIA Sharepoint page.

EIA Recommendations

The EIA Recommendation is the result of the EIA process and recommends real and tangible steps to improve policy, program or service delivery to achieve more equitable outcomes for the community. In order to be compliant with the Act, EIA recommendations should include how this recommendation meets the different needs of different genders, addresses gender inequality and promotes gender equality.

An important thing for staff to remember when developing an EIA recommendation is that little wins make big impacts. Staff do not need to overhaul the policy, program or service or introduce sweeping changes in order to create meaningful change. Staff can identify a gap as simple as not collecting data on gender and commence gender data collection that can later be analysed for trends.

EIA recommendations are reportable to the Commissioner through a biennial progress and evidence the City's compliance with the Act.

Implementing an EIA recommendation

Once an EIA has been completed and an EIA recommendation has been made, it will be reviewed by the appropriate people manager and upon approval, will be assigned to the policy, program or service lead for implementation in the *Equity For All (E4A) Gender Equity Action Plan* and visible in PULSE for quarterly reporting.

Policy, program and service leads can refer to the complete EIA template on ECM to support them as they seek to implement the EIA recommendation according to its intent and achieve more equitable outcomes in the community.

EIA Champions

EIA Champions are staff members who are trained to support other staff conduct Equity Impact Assessments. They ensure EIAs are conducted in a way that is legislatively compliant with the Act, facilitate workshops to support staff explore ideas of equity and inclusion and mentor staff conducting EIAs.

Who is an EIA Champion at the City?

EIA Champions are appointed in each Directorate with the aim to have an EIA Champion appointed in each unit (some exemptions apply) by the end of 2023. EIA Champions are listed on the **EIA Sharepoint page**.

How to become an EIA Champion

EIA Champions promote equitable policy, program and service delivery at the City and engage with a diverse cohort of staff across varying units and directorates on a regular basis. They gain unique insights into how different genders and diverse groups engage with the City's policies, programs and services and what barriers and enablers are at play in achieving a fair and inclusive community.

Staff are encouraged to consider the EIA Champion role if they possess/aspire to possess the following attributes:

- Open-minded and tolerant of a broad spectrum of beliefs, ideas and perspectives
- Foster environments in which people feel safe and welcomed to share honestly
- Are confident to facilitate discussion with groups of diverse staff and ensure all voices are heard
- Able to keep others on track, complete projects and meet deadlines

EIA Champions receive specialised training to learn the fundamentals of gender equity and social justice, workshop facilitation and responding to backlash and resistance.

EIA Champions are requested to facilitate 1 x EIA per quarter which will amount to the equivalent of 2 x working days per quarter. EIA Champions will also be asked to be a point of reference for staff in their unit who may have queries or seek advice about the EIA process.

If you would like to become an EIA Champion, please express your interest to **ge@bendigo.vic.gov.au** and cc' your People Manager following a discussion with your People Manager.

EIA Champion Guide

EIA Champions are supported by the City's Gender Equity Officers and an EIA Champion Guide which provides tailored tools to support facilitating the EIA process, including email templates and FAQs. The EIA Champion Guide can be accessed via the **EIA Sharepoint page**.

Monitoring, learning and evaluation plan

The EIA process includes a monitoring, learning and evaluation plan that will support ongoing improvement for the EIA process itself and ensure EIA recommendations are producing equitable outcomes for the community.

EIA Case Studies

The City has a growing library of **EIA case studies** which outline the improvements that have been recommended to individual policies, programs and services following an assessment of how they affect different genders and diverse groups in different ways.

EIA case studies celebrate little wins towards equality in policy, program and service delivery and demonstrate that even the smallest of changes can make meaningful progress towards achieving a fair and inclusive community.

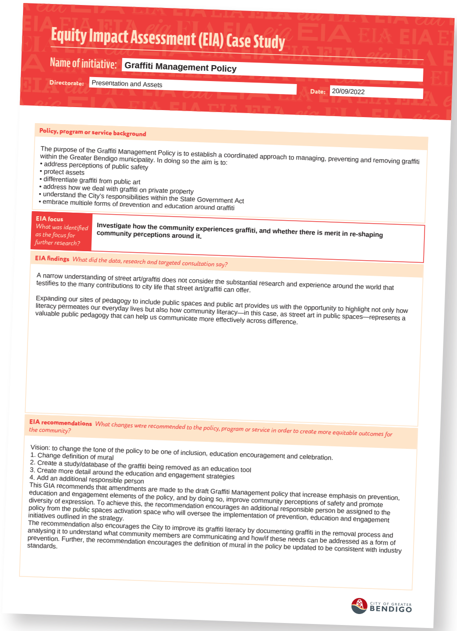

Equity Impact Assessment (EIA) Case Study

Name of initiative: Graffiti Management Policy

Directorate: Presentation and Assets

Date: 20/09/2022

Policy, program or service background

The purpose of the Graffiti Management Policy is to establish a coordinated approach to managing, preventing and removing graffiti within the Greater Bendigo municipality. In doing so the aim is to:
• address perceptions of public safety
• protect assets
• differentiate graffiti from public art
• address how we deal with graffiti on private property
• understand the City's responsibilities within the State Government Act
• embrace multiple forms of prevention and education around graffiti

EIA focus *What was identified as the focus for further research?*

Investigate how the community experiences graffiti, and whether there is merit in re-shaping community perceptions around it.

EIA findings *What did the data, research and targeted consultation say?*

A narrow understanding of street art/graffiti does not consider the substantial research and experience around the world that testifies to the many contributions to city life that street art/graffiti can offer.

Expanding our sites of pedagogy to include public spaces and public art provides us with the opportunity to highlight not only how literacy permeates our everyday lives but also how community literacy—in this case, as street art in public spaces—represents a valuable public pedagogy that can help us communicate more effectively across difference.

EIA recommendations *What changes were recommended to the policy, program or service in order to create more equitable outcomes for the community?*

Vision: to change the tone of the policy to be one of inclusion, education encouragement and celebration.
1. Change definition of mural
2. Create a study/database of the graffiti being removed as an education tool
3. Create more detail around the education and engagement strategies
4. Add an additional responsible person

This GIA recommends that amendments are made to the draft Graffiti Management policy that increase emphasis on prevention, education and engagement elements of the policy, and by doing so, improve community perceptions of safety and promote diversity of expression. To achieve this, the recommendation encourages an additional responsible person be assigned to the policy from the public spaces activation space who will oversee the implementation of prevention, education and engagement initiatives outlined in the strategy.

The recommendation also encourages the City to improve its graffiti literacy by documenting graffiti in the removal process and analysing it to understand what community members are communicating and how/if these needs can be addressed as a form of prevention. Further, the recommendation encourages the definition of mural in the policy be updated to be consistent with industry standards.

CITY OF GREATER BENDIGO

Frequently used terminology

Gender – The socially learnt roles, behaviours, activities and attributes that any given society considers appropriate for men and women, such as definitions of masculinity and femininity. Gender expectations vary between cultures and can change over time.

Gender diverse and non-binary – People who do not identify as woman or man. In the same way that sexual orientation and gender expression are not binaries, gender identity is not a binary either. Some people may identify as agender (having no gender), bigender (both a woman and a man) or non-binary (neither woman nor man). There is a diverse range of gender identities such as genderqueer, gender neutral, genderfluid and third gendered. It is important to be aware that language in this space is still evolving and people may have their own preferred gender identities that are not listed here (see Government of Victoria, Inclusive Language Guide).

Gender equality – Involves equality of opportunity and equality of results. It includes the redistribution of resources and responsibilities between men and women and the transformation of the underlying causes and structures of gender inequality to achieve substantive equality. It is about recognising diversity and disadvantage to ensure equal outcomes for all and therefore often requires women-specific programs and policies to end existing inequalities.

Gender Equality Action Plan (GEAP) – Certain organisations (defined entities) must develop a Gender Equality Action Plan to meet their obligations under the Gender Equality Act. A GEAP will help an organisation plan, implement and measure change in order to achieve workplace gender equality.

Gender impact assessment – Gender impact assessments are a way of critically thinking about how policies, programs and services will meet the different needs of women, men and gender diverse people.

Gender inequality – The unequal distribution of power, resources, opportunity, and value afforded to men and women in a society due to prevailing gendered norms and structures.

Gender roles – The functions and responsibilities expected to be fulfilled by women and men, girls and boys in a given society.

Inclusive language – The Gender Equality Act 2020 and the Gender Impact Assessment Toolkit uses inclusive language when referring to genders. When referring to 'woman/women' or 'man/men' it refers to female or male identifying people; this includes transgender people, cisgender people, and others who identify themselves within the spectrum of the gender identity of woman or man. When referring to the term 'gender diverse people', this includes people who may identify as non-binary, trans, agender, genderqueer, genderfluid or with any other term.

Intersectionality – Refers to the ways in which different aspects of a person's identity can expose them to overlapping forms of discrimination and marginalisation. These aspects can include (but are not limited to) gender, ethnicity and cultural background, language, socioeconomic status, disability, sexual orientation, religion, age, geographic location or visa status.

Intersectional discrimination – Discrimination that takes place on the basis of several multiple grounds or characteristics/identities which operate and interact with each other at the same time in such a way as to make them inseparable. The concept of intersectional discrimination is sometimes referred to as 'intersectionality.'

Sex – The biological and physical characteristics used to define humans as male, female or intersex.

Social norms - The rules of conduct and models of behaviour expected by a society or social group. They are grounded in the customs, traditions and value systems that develop over time in a society or social group.

Violence Against Women (VAW) - Any act of gender-based violence that causes or could cause physical, sexual or psychological harm or suffering to women, including threats of harm or coercion, in public or in private life.

Equity Impact Assessment

Policy name:

Template A:
Policies

Date completed:

Policy lead:

EIA Recommendation approved by:

Equity Impact Assessment (EIA) Executive Summary

Policy name:

Directorate: **Date:**

Policy, program or service background

EIA focus
What was identified as the focus for further research?

EIA findings *What did the data, research and targeted consultation say?*

EIA recommendations *What changes were recommended to the policy, program or service in order to create more equitable outcomes for the community?*

Completing your EIA

Read this template in full before you start. An estimated time for each segment is located in brackets next to each heading.

NB: Questions marked with an 'x' are mandatory and will be used for the executive summary.

Quick facts (15 minutes)

1. Name of policy*:

2. Directorate*:

3. Full name of policy lead:

4. Is this EIA self-led or facilitator-led?:

5. Brief overview of policy*: *(max 1 paragraph)*

Step 1, week 1

Define the issues and challenge assumptions (2 hours)

This step can be either self-led or facilitator-led. Please note approval from a Gender Equity Officer is required before commencing a self-led EIA.

Self-led: Complete the questions in the form below. If you think you'd benefit from other perspectives, schedule a 90 minute meeting with 1-2 colleagues or external contractors (if relevant) who are directly involved in the policy, or who will offer a diverse lens. Use the **EIA Workshop PPT A** to help you brainstorm how people of different genders and diverse groups may be impacted by the policy and arrive at a focus for further investigation. Following the workshop, populate the template below using discussion notes from the meeting.

Facilitator-led: An EIA Champion will host a 90 minute workshop with you and 3-4 colleagues or external contractors (if relevant) who are directly involved in the policy, or who will offer a diverse lens. Following the workshop, populate the template below using discussion notes from MS Teams Chat.

GENERAL

Q1	**What is the issue the policy is trying to address? Why is it needed?** *Be as specific as you can.*

Q2	**Who is impacted by the policy?** Are some more impacted than others?

PEOPLE LENS – GENDER

Q3	**Do the different social roles and responsibilities people take on influence how they are impacted by the policy?** Consider gender norms, and how these might differ across multi-cultural or religious communities.

Q4	**What are the different needs of women, men and gender diverse people who are impacted by the policy? Are there barriers that could be addressed to better meet these needs?**

PEOPLE LENS – INTERSECTIONALITY

Q5	**What needs might there be for people who experience gender inequality alongside other forms of discrimination?** Consider identity factors such as Aboriginality, age, disability, ethnicity, sexuality and religion. **What action can be taken to address these needs?**

PLACE LENS

Q6	**What impact does location (neighbourhood/area/region) have on the needs of diverse people impacted by the policy? How can this be addressed?**

EXPERIENCE LENS

Q7 **What experiences may prevent people from benefiting from the policy? How can this be addressed?**

COMMUNITY ENGAGEMENT

Q8 **What barriers and enablers might influence diverse participation in community engagement?** Consider literacy (English, digital and language of origin), time or financial barriers, venue accessibility, level of trust in government bodies, extent and diversity of reach.

EIA FOCUS

Q9 **What will you focus on in your assessment to support a more equitable outcome for the community?** Ensure your focus addresses gender inequality to be compliant with the *Gender Equality Act 2020*.

Note: This response forms the focus of this Equity Impact Assessment and will guide the remainder of the template. Summarise in 1-2 sentences the EIA focus the group agreed to and ensure this is the focus of your research (step 2) and options development and analysis (step 3). It often helps to phrase this in the form of a question you will endeavour to answer.

END OF WEEK 1 CHECKLIST

- ☐ Workshop discussion complete
- ☐ Step 1 questions populated using responses from all participants
- ☐ EIA focus is a clear and concise summary of agreed focus of group

Understand the context (4 hours)

This step is self-led

- Undertake further research on your EIA focus
- Use internal data, desktop research or stakeholder engagement to investigate further

INTERNAL DATA

Q1	**What internal data is already available that you could use to support your research?** List data sources used below and explain findings in Q4.

DESKTOP RESEARCH

Q2	**Review existing research online for information to support the development or review of your policy.** You may consider doing some benchmarking to see what other organisations are doing. Refer to pages 16-17 of the **Gender Equality Commission templates and resources** for a list of Victorian and National data sources. List research sources below and explain findings in Q4.

CONSULTATION AND MEANINGFUL STAKEHOLDER ENGAGEMENT

Q3 **Do you need to conduct additional consultation and/or meaningful stakeholder engagement for your EIA focus?** If so, list stakeholders specifically targeted or engaged below and explain findings in Q4.

Things to consider if you need to undertake further engagement:

- Stakeholders such as internal council officers or reference groups, existing stakeholder lists/ databases, people with lived experience, local women's organisations or peak bodies that represent diverse groups
- If there are already existing mechanisms in place to seek stakeholder views that could be used for this engagement. i.e. Let's Talk

If engaging a group of community members, consider:

- Time of day and accessible venue
- If interpretation services are required
- If a payment/honorarium/stipend is required to address financial barriers to participation
- How you will close the loop with participating stakeholders in your EIA

Q4 **What did the internal data, desktop research and targeted consultation tell you?** *(1 paragraph high level summary)*

Q5 **Did your research identify any barriers to equity at a state or federal level in relation to the policy?** If YES, please provide some detail on the barrier/s identified to support the City in its role as Advocate to other levels of government.

◉ Yes ○ No ○ Unknown

END OF WEEK 2 CHECKLIST

- ☐ Internal data reviewed
- ☐ Desktop research conducted
- ☐ Targeted consultation and engagement conducted (if required)
- ☐ A summary of findings provided
- ☐ Advocacy opportunities considered

Options analysis and development (2 hours)

This step is self-led

- Use the information you have gathered from steps 1 and 2 to develop 1-2 option/s to improve the policy to meet the needs of different genders and diverse groups, and promote a more equitable community.
- Consider the benefits, costs and overall equity impact of the option/s

For each option that is proposed, complete parts A, B and C (one option might be to leave the policy as is and to assess the cost/benefit of this).

PART A: Describe your options

Using the data and evidence you have collected, develop an option or options to address your policy issue.

(NB: one option might be to leave the policy as is and to assess the cost/benefit and equity impact of this).

[For each option, describe the proposed solution to the policy you are working on. Include a description of the overall aim and objective that you are trying to achieve, and a description of who you have identified as the target audience. Provide a brief description of the proposed changes and how they will meet the needs and create benefits for your targeted audience.]

Option 1	Option 2

PART B: Describe the equity benefits and costs of each option

Use the table below to identify the benefits and costs of proceeding with the proposed option/s to the policy.

The *Gender Equality Act 2020* requires you to show how the policy will meet the needs of persons of different genders, address gender inequality and promote gender equality.

See if you can address these in addition to broader equity considerations when you are identifying the costs and benefits of each option. Below are some guiding questions to assist you however, remember that your responses should be based on the research, data, consultations and analysis relevant to your context. Be as specific as you can in terms of the changes you want to see.

	Option 1 - Benefits	Option 2 - Benefits
**Example guiding questions**<br>• Will some people benefit more because they have greater access, or does this policy do everything it can to ensure resources are distributed and used equally?<br>• Will it contribute to transforming gender norms in a positive way? For example, will it contribute to a more balanced distribution of unpaid care, labour and family responsibilities between men and women?<br>• Will it make women and girls safer in public or private spaces?		
	**Option 1 - Costs and risks**	**Option 2 - Costs and risks**
**Example guiding questions**<br>• Who is likely to be negatively impacted by this? How are the most vulnerable groups likely to be impacted?<br>• Will this reduce a certain group's access to economic resources or opportunities? If so, are they already disadvantaged?<br>• Does it reinforce harmful gender stereotypes, for example, by further promoting men in an already male dominated industry?		

PART C: Overall equity impact

Consider your cost and benefit analysis. Do the benefits outweigh the costs or does the policy potentially have unintended negative consequences for certain groups of people?

Score your option on the continuum below using the guide to determine if it has a positive, neutral or negative overall equity impact, then briefly outline your reasons for this.

-5	-4	-3	-2	-1	0	1	2	3	4	5

NEGATIVE EQUITY IMPACT	NEUTRAL EQUITY IMPACT	POSITIVE EQUITY IMPACT
• Reinforces traditional gender norms/ stereotypes<br>• Reinforces privilege to those already privileged in this area<br>• Doesn't level the playing field<br>• Doesn't create any new opportunities for access and inclusion	• Does not appear to encourage or discourage access, inclusion or equity	• Considers the different needs of different genders<br>• Takes action to remove barriers or create new opportunities for access and inclusion for under-represented user groups<br>• Takes into account the interplay of multiple forms of discrimination.<br>• Levels the playing field

How did Option 1 and 2 score? Explain in 1-2 sentences the rationale for the score.

Option 1 - Score	Option 2 - Score

END OF WEEK 3 CHECKLIST

- ☐ Developed 1-2 options which propose meaningful changes to the policy
- ☐ Conducted an equity cost/benefit analysis for each option proposed
- ☐ Scored the overall equity impact of each option and provided reasoning

Make a final recommendation (1.5 hours)

This step is self-led or facilitator-led

- Decide on which option to adopt for recommendation
- Make recommendation with supporting rationale

The EIA Recommendation* Select an option to make as an EIA recommendation and outline the proposed change/s to the policy in a way that can be easily understood and implemented by a colleague. Remember that little wins achieve big impacts. The improvement/s you recommend do not need to be big or complex in order to create meaningful progress towards equitable outcomes.

Provide a supporting rationale of how this recommendation meets the needs of different genders and diverse people, addresses gender inequality; and promotes gender equality in order to be compliant with the *Gender Equality Act 2020*.

Approval of EIA recommendation. The EIA recommendation above needs approval to be actioned. As people manager of the policy, program or service lead, please review the EIA recommendation and provide it with full, partial or no approval to be actioned with a supporting rationale if necessary. If approval needs to be escalated, please inform ge@bendigo.vic.gov.au who it needs to be assigned to for approval. The EIA recommendation outlined above is:

○ **Fully approved** ○ **Partially approved*** ◉ **Not approved***

*If partially approved, please specify which components are/aren't approved with a supporting rationale below. If not approved, please provide a supporting rationale below.

Full name, job title (Approver): **Date:**

END OF WEEK 4 CHECKLIST

- ☐ Selected an option to be an EIA recommendation
- ☐ Outlined the proposed changes in a way that can be understood and implemented by a colleague
- ☐ Provided supporting rationale of how the EIA recommendation meets the needs of different genders and diverse people, addresses gender inequality and promotes gender equality to be compliant with the *Gender Equality Act 2020*

Equity Impact Assessment

Program or service name:

Template B:
Programs and services

Date completed:

Program or service lead:

EIA Recommendation approved by:

CITY OF GREATER BENDIGO

Equity Impact Assessment (EIA) Executive Summary

Program or service name:

Directorate: **Date:**

**Program or service background**

**EIA focus** *What was identified as the focus for further research?*	

**EIA findings** *What did the data, research and targeted consultation say?*

**EIA recommendations** *What changes were recommended to the program or service in order to create more equitable outcomes for the community?*

Completing your EIA

Read this template in full before you start. An estimated time for each segment is located in brackets next to each heading.

NB: Questions marked with an 'x' are mandatory and will be used for the executive summary.

Quick facts (15 minutes)

1. Name of program or service*:

2. Directorate*:

3. Full name of program or service lead:

4. Is this EIA self-led or facilitator-led?:

5. Brief overview of program or service*: *(max 1 paragraph)*

Define the issues and challenge assumptions (2 hours)

This step can be either self-led or facilitator-led. Please note approval from a Gender Equity Officer is required before commencing a self-led EIA.

Self-led: Complete the questions in the form below. If you think you'd benefit from other perspectives, schedule a 90 minute meeting with 1-2 colleagues or external contractors (if relevant) who are directly involved in the program or service, or who will offer a diverse lens. Use the **EIA Workshop PPT B** to help you brainstorm how people of different genders and diverse groups may be impacted by the program or service and arrive at a focus for further investigation. Following the workshop, populate the template below using discussion notes from the meeting.

Facilitator-led: An EIA Champion will host a 90 minute workshop with you and 3-4 colleagues or external contractors (if relevant) who are directly involved in the program or service, or who will offer a diverse lens. Following the workshop, the nominated EIA Lead will populate the template below using discussion notes from MS Teams Chat.

GENERAL

Q1	**What is the issue the program or service is trying to address? Why is it needed?** *Be as specific as you can.*

Q2	**Who is impacted by the program or service?** Are any groups under-represented or missing?

PEOPLE LENS – GENDER

Q3	**Do the different social roles and responsibilities that people take on affect the way people access and use this program or service?** Consider gender norms, and how these might differ across multi-cultural or religious communities.

Q4	**What are the different needs of women, men and gender diverse people who use this program or service? Are there barriers that could be addressed to better meet these needs?**

PEOPLE LENS – INTERSECTIONALITY

Q5	**What needs might there be for people who experience gender inequality alongside other forms of discrimination?** Consider identity factors such as Aboriginality, age, disability, ethnicity, sexuality and religion.

PLACE LENS

Q6	**What impact does location (neighbourhood/area/region) have on the needs of diverse people engaging with the program or service? How can this be addressed?**

EXPERIENCE LENS

Q7	What experiences may prevent people from accessing the program or service? How can this be addressed?

COMMUNITY ENGAGEMENT

Q8	**What barriers and enablers might influence diverse participation in community engagement?** Consider literacy (English, digital and language of origin), time or financial barriers, venue accessibility, level of trust in government bodies, extent and diversity of reach.

EIA FOCUS

Q9	**What will you focus on in your assessment to support a more equitable outcome for the community?** Ensure your focus addresses gender inequality to be compliant with the *Gender Equality Act 2020*.<br>*Note: This response forms the focus of this Equity Impact Assessment and will guide the remainder of the template. Summarise in 1-2 sentences the EIA focus the group agreed to and ensure this is the focus of your research (step 2) and options development and analysis (step 3). It often helps to phrase this in the form of a question you will endeavour to answer. E.g. 'Are gender norms impacting who is using skate parks in Bendigo?'*

END OF WEEK 1 CHECKLIST

- ☐ Workshop discussion complete
- ☐ Step 1 questions populated using responses from all participants
- ☐ EIA focus is a clear and concise summary of agreed focus of group

Understand the context (4 hours)

This step is self-led

- Undertake further research on your EIA focus
- Use internal data, desktop research or stakeholder engagement to investigate further

INTERNAL DATA

Q1	**What internal data is already available that you could use to support your research?** List data sources used below and explain findings in Q4.

DESKTOP RESEARCH

Q2	**Review existing research online for information to support the development or review of your program or service.** You may consider doing some benchmarking to see what other organisations are doing. Refer to pages 16-17 of the **Gender Equality Commission templates and resources** for a list of Victorian and National data sources. List research sources below and explain findings in Q4.

CONSULTATION AND MEANINGFUL STAKEHOLDER ENGAGEMENT

Q3 **Do you need to conduct additional consultation and/or meaningful stakeholder engagement for your EIA focus?** If so, list stakeholders specifically targeted or engaged below and explain findings in Q4.

Things to consider if you need to undertake further engagement:

- Stakeholders such as internal council officers or reference groups, existing stakeholder lists/ databases, people with lived experience, local women's organisations or peak bodies that represent diverse groups
- If there are already existing mechanisms in place to seek stakeholder views that could be used for this engagement. i.e. Let's Talk

If engaging a group of community members, consider:

- Time of day and accessible venue
- If interpretation services are required
- If a payment/honorarium/stipend is required to address financial barriers to participation
- How you will close the loop with participating stakeholders in your EIA

Q4 **What did the internal data, desktop research and targeted consultation tell you?*** *(1 paragraph high level summary)*

Q5 **Did your research identify any barriers to equity at a state or federal level in relation to the program or service which can inform advocacy work?** If YES, please provide some detail on the barrier/s identified to support the City in its role as Advocate to other levels of government.

○ Yes ○ No ○ Unknown

END OF WEEK 2 CHECKLIST

- ☐ Internal data reviewed
- ☐ Desktop research conducted
- ☐ Targeted consultation and engagement conducted (if required)
- ☐ A summary of findings provided
- ☐ Advocacy opportunities considered

Options analysis and development (2 hours)

This step is self-led

- Use the information you have gathered from steps 1 and 2 to develop 1-2 option/s to improve the program or service to meet the needs of different genders and diverse groups, and promote a more equitable community.
- Consider the benefits, costs and overall equity impact of the option/s

For each option that is proposed, complete parts A, B and C (one option might be to leave the program or service as is and to assess the cost/benefit of this).

PART A: Describe your options

Using the data and evidence you have collected, develop an option or options to address your program or service issue.

(NB: one option might be to leave the program or service as is and to assess the cost/benefit and equity impact of this).

[For each option, describe the proposed solution to the program or service you are working on. Include a description of the overall aim and objective that you are trying to achieve, and a description of who you have identified as the target audience. Provide a brief description of the proposed changes and how they will meet the needs and create benefits for your targeted audience.]

Option 1	Option 2

PART B: Describe the equity benefits and costs of each option

Use the table below to identify the benefits and costs of proceeding with the proposed option/s to the program or service.

The *Gender Equality Act 2020* requires you to show how the program or service will meet the needs of persons of different genders, address gender inequality and promote gender equality.

See if you can address these in addition to broader equity considerations when you are identifying the costs and benefits of each option. Below are some guiding questions to assist you however, remember that your responses should be based on the research, data, consultations and analysis relevant to your context. Be as specific as you can in terms of the changes you want to see.

	Option 1 - Benefits	Option 2 - Benefits
**Example guiding questions**<br>• Will some people benefit more because they have greater access, or does this program or service do everything it can to ensure resources are distributed and used equally?<br>• Will it contribute to transforming gender norms in a positive way? For example, will it contribute to a more balanced distribution of unpaid care, labour and family responsibilities between men and women?<br>• Will it make women and girls safer in public or private spaces?		
	**Option 1 - Costs and risks**	**Option 2 - Costs and risks**
**Example guiding questions**<br>• Who is likely to be negatively impacted by this? How are the most vulnerable groups likely to be impacted?<br>• Will this reduce a certain group's access to economic resources or opportunities? If so, are they already disadvantaged?<br>• Does it reinforce harmful gender stereotypes, for example, by further promoting men in an already male dominated industry?		

PART C: Overall equity impact

Consider your cost and benefit analysis. Do the benefits outweigh the costs or does the program or service potentially have unintended negative consequences for certain groups of people?

Score your option on the continuum below using the guide to determine if it has a positive, neutral or negative overall equity impact, then briefly outline your reasons for this.

-5 -4 -3 -2 -1 0 1 2 3 4 5

NEGATIVE EQUITY IMPACT	NEUTRAL EQUITY IMPACT	POSITIVE EQUITY IMPACT
• Reinforces traditional gender norms/ stereotypes<br>• Reinforces privilege to those already privileged in this area<br>• Doesn't level the playing field<br>• Doesn't create any new opportunities for access and inclusion	• Does not appear to encourage or discourage access, inclusion or equity	• Considers the different needs of different genders<br>• Takes action to remove barriers or create new opportunities for access and inclusion for under-represented user groups<br>• Takes into account the interplay of multiple forms of discrimination<br>• Levels the playing field

How did Option 1 and 2 score? Explain in 1-2 sentences the rationale for the score.

Option 1 - Score	Option 2 - Score

END OF WEEK 3 CHECKLIST

- ☐ Developed 1-2 options which propose meaningful changes to the program or service
- ☐ Conducted an equity cost/benefit analysis for each option proposed
- ☐ Scored the overall equity impact of each option and provided reasoning

Step 4, week 4

Make a final recommendation (1.5 hours)

This step is self-led or facilitator-led

- Decide on which option to adopt for recommendation
- Make recommendation with supporting rationale

The EIA Recommendation* Select an option to make as an EIA recommendation and outline the proposed change/s to the program or service in a way that can be easily understood and implemented by a colleague. Remember that little wins achieve big impacts. The improvement/s you recommend do not need to be big or complex in order to create meaningful progress towards equitable outcomes.

Provide a supporting rationale of how this recommendation meets the needs of different genders and diverse people, addresses gender inequality; and promotes gender equality in order to be compliant with the *Gender Equality Act 2020*.

Approval of EIA recommendation. The EIA recommendation above needs approval to be actioned. As people manager of the policy, program or service lead, please review the EIA recommendation and provide it with full, partial or no approval to be actioned with a supporting rationale if necessary. If approval needs to be escalated, please inform ge@bendigo.vic.gov.au who it needs to be assigned to for approval. The EIA recommendation outlined above is:

◯ Fully approved ◯ Partially approved* ◯ Not approved*

*If partially approved, please specify which components are/aren't approved with a supporting rationale below. If not approved, please provide a supporting rationale below.

Full name, job title (Approver): **Date:**

END OF WEEK 4 CHECKLIST

- ☐ Selected an option to be an EIA recommendation
- ☐ Outlined the proposed changes in a way that can be understood and implemented by a colleague
- ☐ Provided supporting rationale of how the EIA recommendation meets the needs of different genders and diverse people, addresses gender inequality and promotes gender equality to be compliant with the *Gender Equality Act 2020*

Equity Impact Assessment

Plan, framework or strategy name:

Template C:

Plans, frameworks and strategies

Date completed:

Plan, framework or strategy lead:

EIA Recommendation approved by:

CITY OF GREATER BENDIGO

Equity Impact Assessment (EIA) Executive Summary

Name of plan, framework or strategy:

Directorate:

Date:

Plan, framework or strategy background

EIA focus
What was identified as the focus for further research?

EIA findings *What did the data, research and targeted consultation say?*

EIA recommendations *What changes were recommended to the plan, framework or strategy in order to create more equitable outcomes for the community?*

Completing your EIA

Read this template in full before you start. An estimated time for each segment is located in brackets next to each heading.

NB: Questions marked with an 'x' are mandatory and will be used for the executive summary.

Quick facts (15 minutes)

1. Name of plan, framework or strategy*:

2. Directorate*:

3. Full name of plan, framework or strategy lead:

4. Is this EIA self-led or facilitator-led?:

5. Brief overview of plan, framework or strategy*: *(max 1 paragraph)*

Define the issues and challenge assumptions (2 hours)

This step can be either self-led or facilitator-led. Please note approval from a Gender Equity Officer is required before commencing a self-led EIA.

Self-led: Complete the questions in the form below. If you think you'd benefit from other perspectives, schedule a 90 minute meeting with 1-2 colleagues or external contractors (if relevant) who are directly involved in the plan, framework or strategy, or who will offer a diverse lens. Use the **EIA Workshop PPT C** to help you brainstorm how people of different genders and diverse groups may be impacted by the plan, framework or strategy and arrive at a focus for further investigation. Following the workshop, populate the template below using discussion notes from the meeting.

Facilitator-led: An EIA Champion will host a 90 minute workshop with you and 3-4 colleagues or external contractors (if relevant) who are directly involved in the plan, framework or strategy, or who will offer a diverse lens. Following the workshop, populate the template below using discussion notes from MS Teams Chat.

GENERAL

Q1	**What is the issue the plan, framework or strategy is trying to address? Why is it needed?** *Be as specific as you can.*

Q2	**a) Who is impacted by the plan, framework or strategy?** Are some more impacted than others?

**b) What are the major elements of the plan, framework or strategy?** Which element might be a focus today?

PEOPLE LENS – GENDER

Q3	**Do the different social roles and responsibilities people take on influence how they are impacted by the plan, framework or strategy?** Consider gender norms, and how these might differ across multi-cultural or religious communities.

Q4	**What are the different needs of women, men and gender diverse people who are impacted by the plan, framework or strategy? Are there barriers that could be addressed to better meet these needs?**

PEOPLE LENS – INTERSECTIONALITY

Q5	**What needs might there be for people who experience gender inequality alongside other forms of discrimination?** Consider identity factors such as Aboriginality, age, disability, ethnicity, sexuality and religion. **What action can be taken to address these needs?**

PLACE LENS

Q6	**What impact does location (neighbourhood/area/region) have on the needs of diverse people impacted by the plan, framework or strategy? How can this be addressed?**

EXPERIENCE LENS

Q7	**What experiences may prevent people from benefiting from the plan, framework or strategy? How can this be addressed?**

COMMUNITY ENGAGEMENT

Q8	**What barriers and enablers might influence diverse participation in community engagement?** Consider literacy (English, digital and language of origin), time or financial barriers, venue accessibility, level of trust in government bodies, extent and diversity of reach.

EIA FOCUS

Q9	**What will you focus on in your assessment to support a more equitable outcome for the community?** Ensure your focus addresses gender inequality to be compliant with the *Gender Equality Act 2020*.<br>*Note: This response forms the focus of this Equity Impact Assessment and will guide the remainder of the template. Summarise in 1-2 sentences the EIA focus the group agreed to and ensure this is the focus of your research (step 2) and options development and analysis (step 3). It often helps to phrase this in the form of a question you will endeavour to answer.*

END OF WEEK 1 CHECKLIST

- ☐ Workshop discussion complete
- ☐ Step 1 questions populated using responses from all participants
- ☐ EIA focus is a clear and concise summary of agreed focus of group

Understand the context (4 hours)

This step is self-led

- Undertake further research on your EIA focus
- Use internal data, desktop research or stakeholder engagement to investigate further

INTERNAL DATA

Q1	**What internal data is already available that you could use to support your research?** List data sources used below and explain findings in Q4.

DESKTOP RESEARCH

Q2	**Review existing research online for information to support the development or review of your plan, framework or strategy.** You may consider doing some benchmarking to see what other organisations are doing. Refer to pages 16-17 of the **Gender Equality Commission templates and resources** for a list of Victorian and National data sources. List research sources below and explain findings in Q4.

CONSULTATION AND MEANINGFUL STAKEHOLDER ENGAGEMENT

Q3 **Do you need to conduct additional consultation and/or meaningful stakeholder engagement for your EIA focus?** If so, list stakeholders specifically targeted or engaged below and explain findings in Q4.

Things to consider if you need to undertake further engagement:

- Stakeholders such as internal council officers or reference groups, existing stakeholder lists/ databases, people with lived experience, local women's organisations or peak bodies that represent diverse groups
- If there are already existing mechanisms in place to seek stakeholder views that could be used for this engagement. i.e. Let's Talk

If engaging a group of community members, consider:

- Time of day and accessible venue
- If interpretation services are required
- If a payment/honorarium/stipend is required to address financial barriers to participation
- How you will close the loop with participating stakeholders in your EIA

Q4 **What did the internal data, desktop research and targeted consultation tell you?**✲ *(1 paragraph high level summary)*

Q5 **Did your research identify any barriers to equity at a state or federal level in relation to the plan, framework or strategy?** If YES, please provide some detail on the barrier/s identified to support the City in its role as Advocate to other levels of government.

○ Yes ○ No ○ Unknown

END OF WEEK 2 CHECKLIST

- ☐ Internal data reviewed
- ☐ Desktop research conducted
- ☐ Targeted consultation and engagement conducted (if required)
- ☐ A summary of findings provided
- ☐ Advocacy opportunities considered

Options analysis and development (2 hours)

This step is self-led

- Use the information you have gathered from steps 1 and 2 to develop 1-2 option/s to improve the plan, framework or strategy to meet the needs of different genders and diverse groups, and promote a more equitable community.
- Consider the benefits, costs and overall equity impact of the option/s

For each option that is proposed, complete parts A, B and C (one option might be to leave the plan, framework or strategy as is and to assess the cost/benefit of this).

PART A: Describe your options

Using the data and evidence you have collected, develop an option or options to address your plan, framework or strategy issue. (NB: one option might be to leave the plan, framework or strategy as is and to assess the cost/benefit and equity impact of this).

[For each option, describe the proposed solution to the plan, framework or strategy you are working on. Include a description of the overall aim and objective that you are trying to achieve, and a description of who you have identified as the target audience. Provide a brief description of the proposed changes and how they will meet the needs and create benefits for your targeted audience.]

Option 1	Option 2

PART B: Describe the equity benefits and costs of each option

Use the table below to identify the benefits and costs of proceeding with the proposed option/s to the plan, framework or strategy.

The *Gender Equality Act 2020* requires you to show how the plan, framework or strategy will meet the needs of persons of different genders, address gender inequality and promote gender equality.

See if you can address these in addition to broader equity considerations when you are identifying the costs and benefits of each option. Below are some guiding questions to assist you however, remember that your responses should be based on the research, data, consultations and analysis relevant to your context. Be as specific as you can in terms of the changes you want to see.

	Option 1 - Benefits	Option 2 - Benefits
**Example guiding questions**<br>• Will some people benefit more because they have greater access, or does this plan, framework or strategy do everything it can to ensure resources are distributed and used equally?<br>• Will it contribute to transforming gender norms in a positive way? For example, will it contribute to a more balanced distribution of unpaid care, labour and family responsibilities between men and women?<br>• Will it make women and girls safer in public or private spaces?		

	Option 1 - Costs and risks	Option 2 - Costs and risks
**Example guiding questions**<br>• Who is likely to be negatively impacted by this? How are the most vulnerable groups likely to be impacted?<br>• Will this reduce a certain group's access to economic resources or opportunities? If so, are they already disadvantaged?<br>• Does it reinforce harmful gender stereotypes, for example, by further promoting men in an already male dominated industry?		

PART C: Overall equity impact

Consider your cost and benefit analysis. Do the benefits outweigh the costs or does the plan, framework or strategy potentially have unintended negative consequences for certain groups of people?

Score your option on the continuum below using the guide to determine if it has a positive, neutral or negative overall equity impact, then briefly outline your reasons for this.

-5	-4	-3	-2	-1	0	1	2	3	4	5

NEGATIVE EQUITY IMPACT	NEUTRAL EQUITY IMPACT	POSITIVE EQUITY IMPACT
• Reinforces traditional gender norms/ stereotypes<br>• Reinforces privilege to those already privileged in this area<br>• Doesn't level the playing field<br>• Doesn't create any new opportunities for access and inclusion	• Does not appear to encourage or discourage access, inclusion or equity	• Considers the different needs of different genders<br>• Takes action to remove barriers or create new opportunities for access and inclusion for under-represented user groups<br>• Takes into account the interplay of multiple forms of discrimination.<br>• Levels the playing field

How did Option 1 and 2 score? Explain in 1-2 sentences the rationale for the score.

Option 1 - Score	Option 2 - Score

END OF WEEK 3 CHECKLIST

- [ ] Developed 1-2 options which propose meaningful changes to the plan, framework or strategy
- [ ] Conducted an equity cost/benefit analysis for each option proposed
- [ ] Scored the overall equity impact of each option and provided reasoning

Make a final recommendation (1.5 hours)

This step is self-led or facilitator-led

- Decide on which option to adopt for recommendation
- Make recommendation with supporting rationale

The EIA Recommendation* Select an option to make as an EIA recommendation and outline the proposed change/s to the plan, framework or strategy in a way that can be easily understood and implemented by a colleague. Remember that little wins achieve big impacts. The improvement/s you recommend do not need to be big or complex in order to create meaningful progress towards equitable outcomes.

Provide a supporting rationale of how this recommendation meets the needs of different genders and diverse people, addresses gender inequality; and promotes gender equality in order to be compliant with the *Gender Equality Act 2020*.

Approval of EIA recommendation. The EIA recommendation above needs approval to be actioned. As people manager of the policy, program or service lead, please review the EIA recommendation and provide it with full, partial or no approval to be actioned with a supporting rationale if necessary. If approval needs to be escalated, please inform ge@bendigo.vic.gov.au who it needs to be assigned to for approval. The EIA recommendation outlined above is:

○ Fully approved ○ Partially approved* ○ Not approved*

*If partially approved, please specify which components are/aren't approved with a supporting rationale below. If not approved, please provide a supporting rationale below.

Full name, job title (Approver): **Date:**

END OF WEEK 4 CHECKLIST

- ☐ Selected an option to be an EIA recommendation
- ☐ Outlined the proposed changes in a way that can be understood and implemented by a colleague
- ☐ Provided supporting rationale of how the EIA recommendation meets the needs of different genders and diverse people, addresses gender inequality and promotes gender equality to be compliant with the *Gender Equality Act 2020*

Equity Impact Assessment (EIA)

Incorporating Gender Impact Assessments as per Gender Equality Act 2020

CITY OF GREATER BENDIGO

Acknowledgment of Country

I acknowledge that the City of Greater Bendigo is on Dja Dja Wurrung and Taungurung Country.

I would like to acknowledge and extend my appreciation for the Dja Dja Wurrung* People, the Traditional Owners of the land that we are standing on today.

Today, we pay our respects to leaders and Elders past, present and future for they hold the memories, the traditions, the culture and the hopes of all Dja Dja Wurrung* Peoples.

We express our gratitude in the sharing of this land, our sorrow for the personal, spiritual and cultural costs of that sharing and our hope that we may walk forward together in harmony and in the spirit of healing.

Introductions:

Co-facilitator

Co-facilitator

EIA Champions/
Observers

Introductions:

What is your connection to the policy?

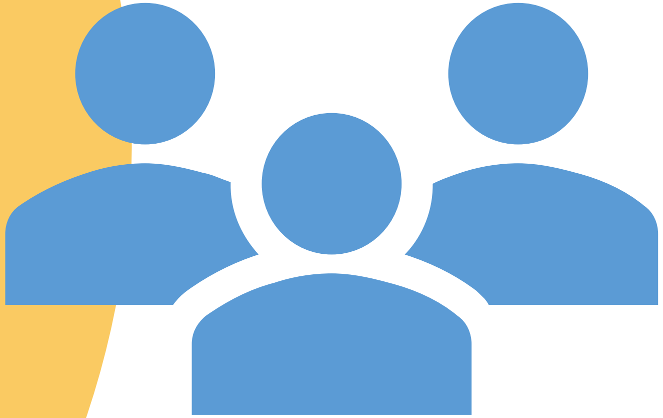

EIA Participants

Introductions:

A 2min overview of the policy

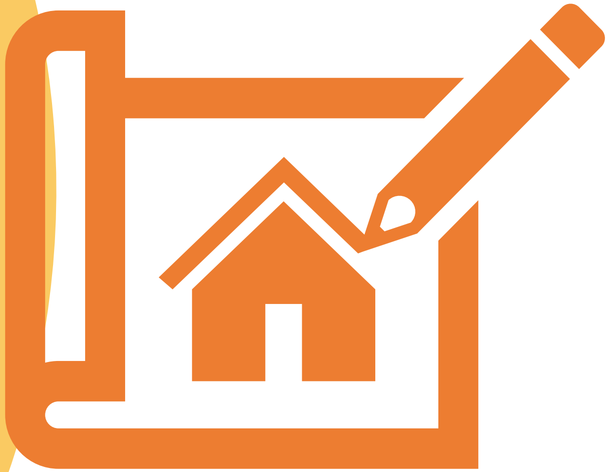

Policy Lead

Gender Impact Assessments:

Gender Equality Act 2020

- Policies
- Programs
- Services
- Direct and significant impact on public
- In development or in review

GIAs help policies, programs and services to:

- meet the different needs of different genders.
- eliminate barriers to gender equality in policy, program and service delivery
- actively promote gender equality

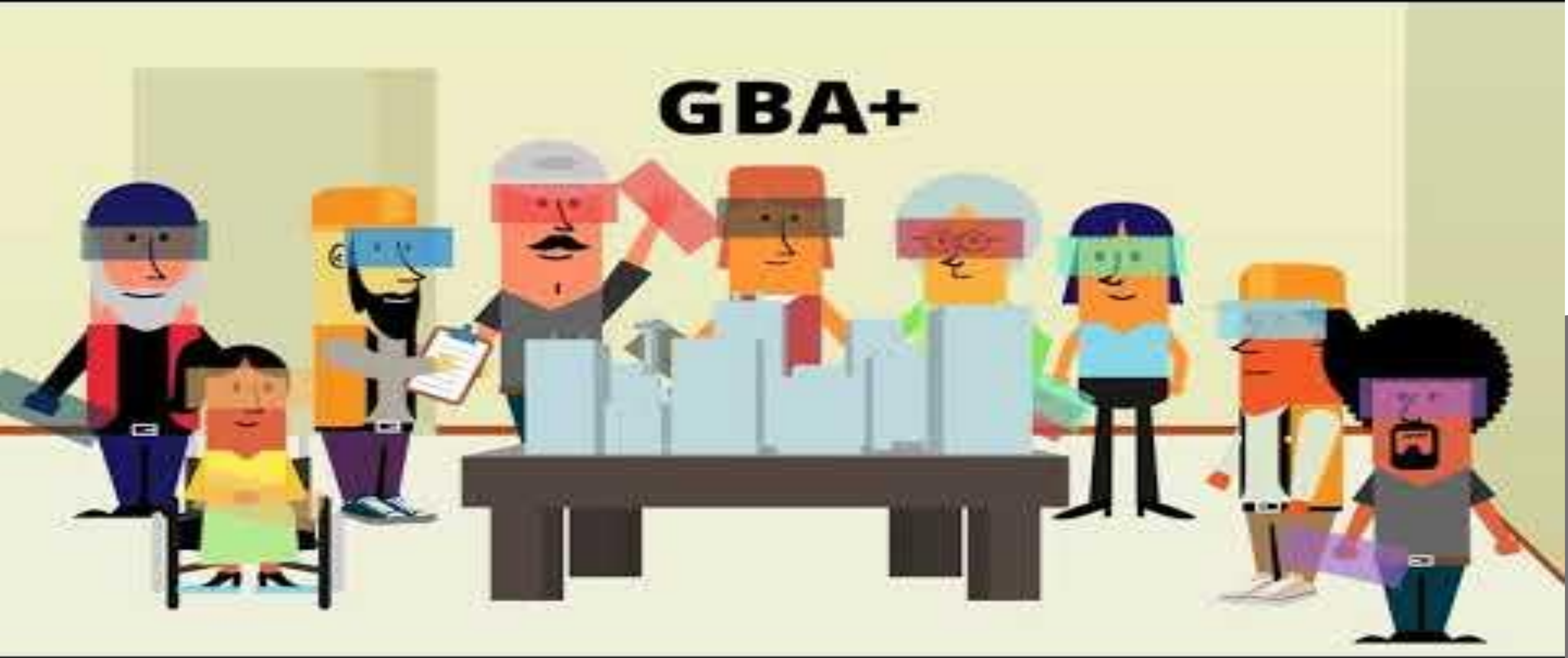
GBA+

What does an EIA involve?

Step 1: Define the issue and challenge assumptions (Today)

Step 2: Undertake further research on EIA focus

Step 3: Propose option/s for meaningful change

Step 4: Make a recommendation

Social Justice Framework 2022-2032

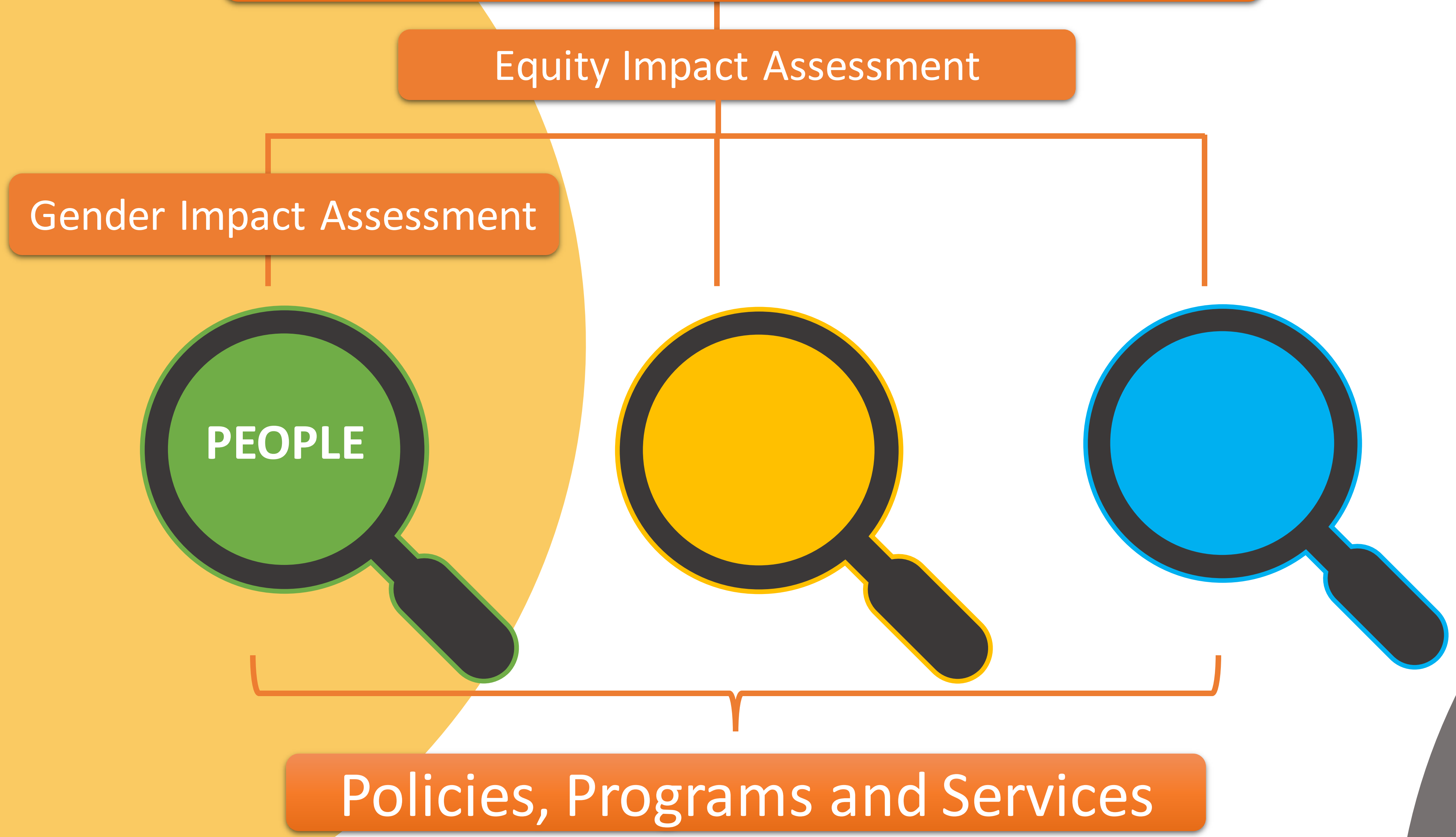

People lens

Helps determine who in Greater Bendigo might be at risk of exclusion, discrimination and disadvantage

- First Nations lens
- Gender lens
- Culture lens
- Lifespan lens (age)
- Abilities/Access lens
- Class/socioeconomic lens
- Sexual orientation (LGBTIQ+) lens
- Religious/faith-based lens

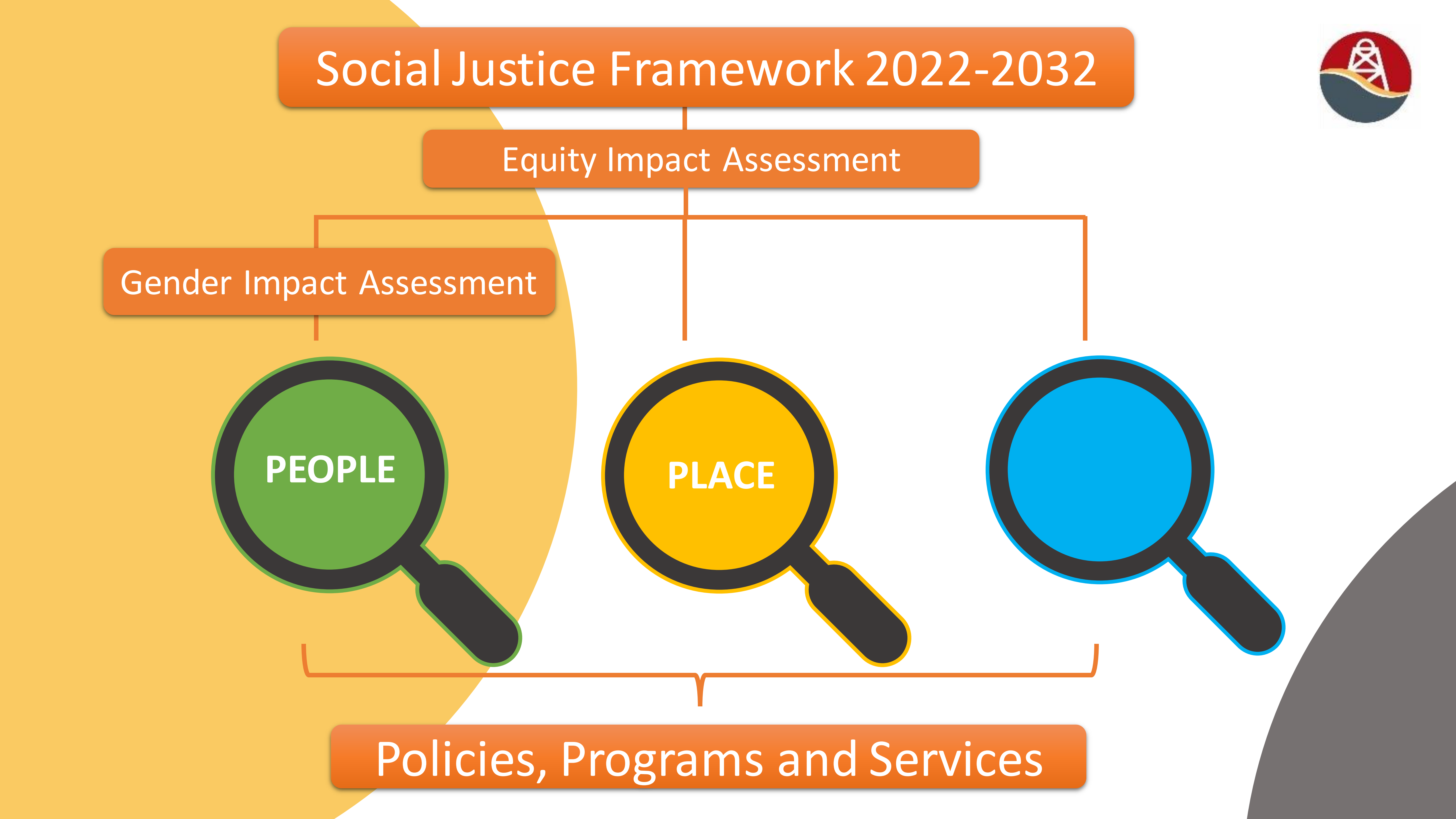
Social Justice Framework 2022-2032
Equity Impact Assessment
Gender Impact Assessment
PEOPLE
PLACE
Policies, Programs and Services

Place lens

Explores the impact of location on the needs of diverse people.

Neighbourhoods

Areas

Regions

Social Justice Framework 2022-2032

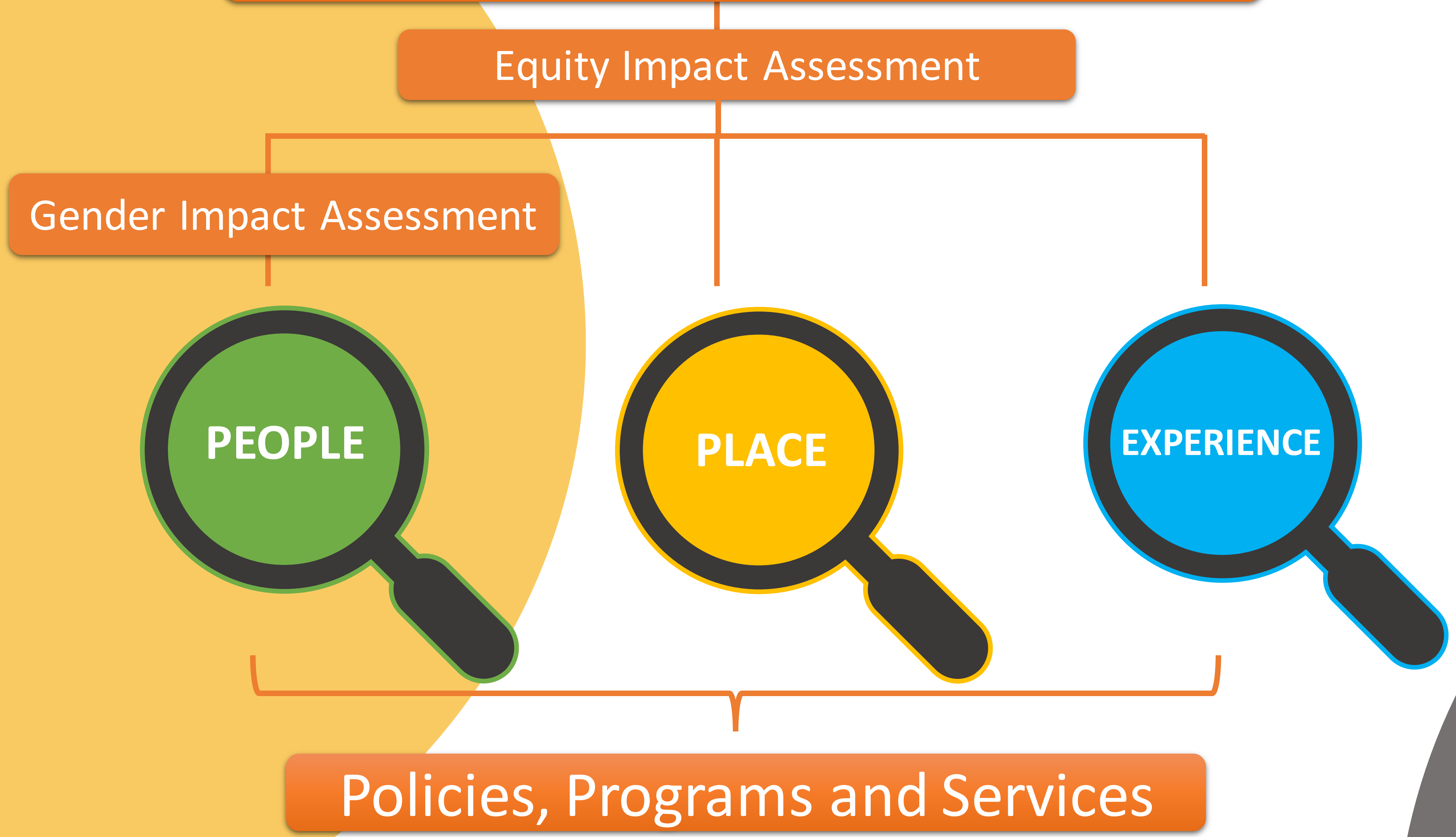

Experience lens

Considers experiences or additional factors that might contribute to exclusion, disadvantage and inequality

- Employment status
- Housing/Homelessness
- Food security
- Migration/refugee/ Seeking asylum
- Past experiences of discrimination
- Family violence
- Household composition (single parent, family with young children, living alone)
- Trauma/ Intergenerational trauma

Intersectionality

Origin of concept based on court case of a group of African-American women alleging a manufacturing company refused to hire them based on their race + gender.

Court ruled against them due to company hiring African-American men on factory floor and white women in reception. Court failed to consider unique discrimination facing African-American women.

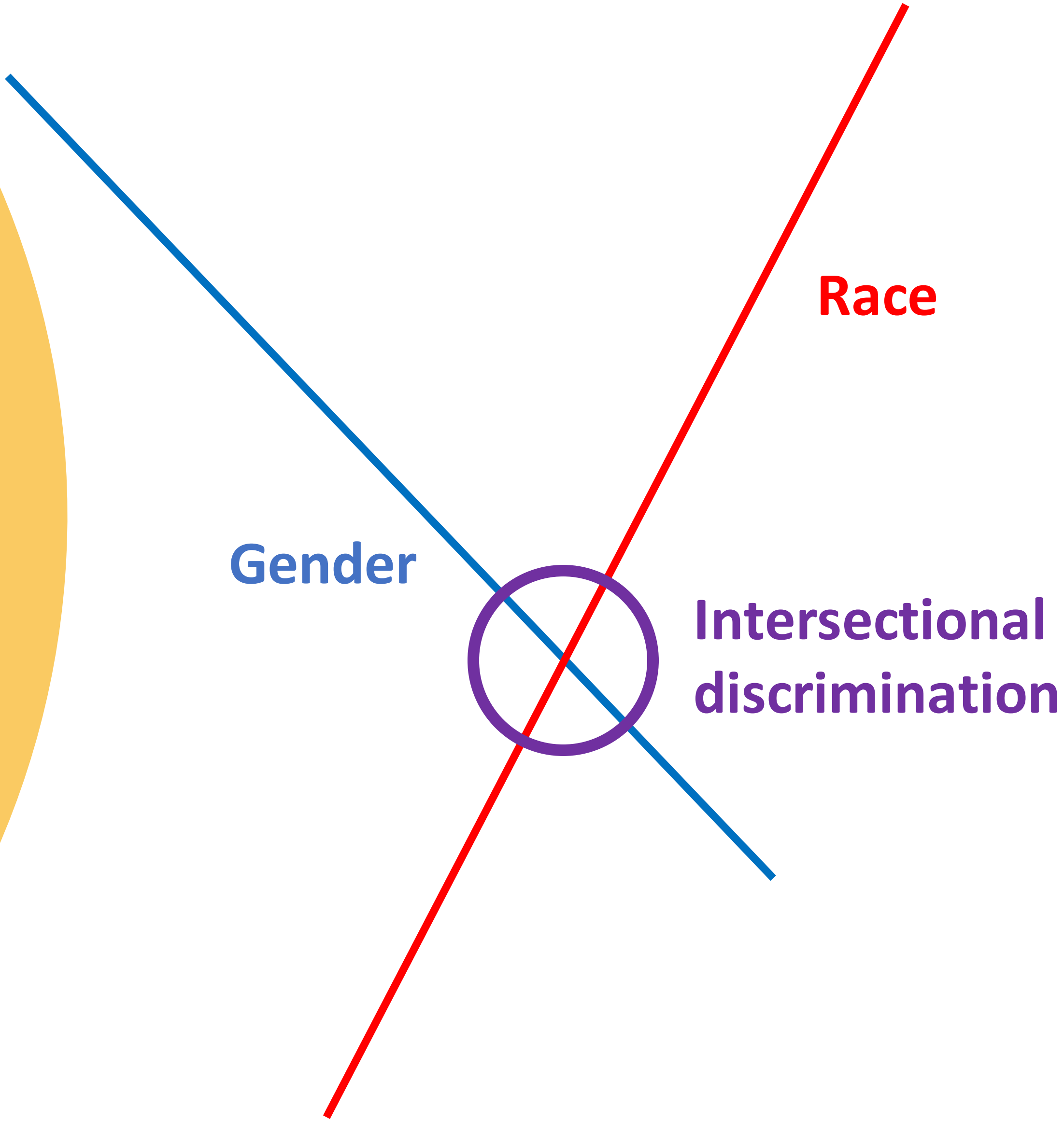

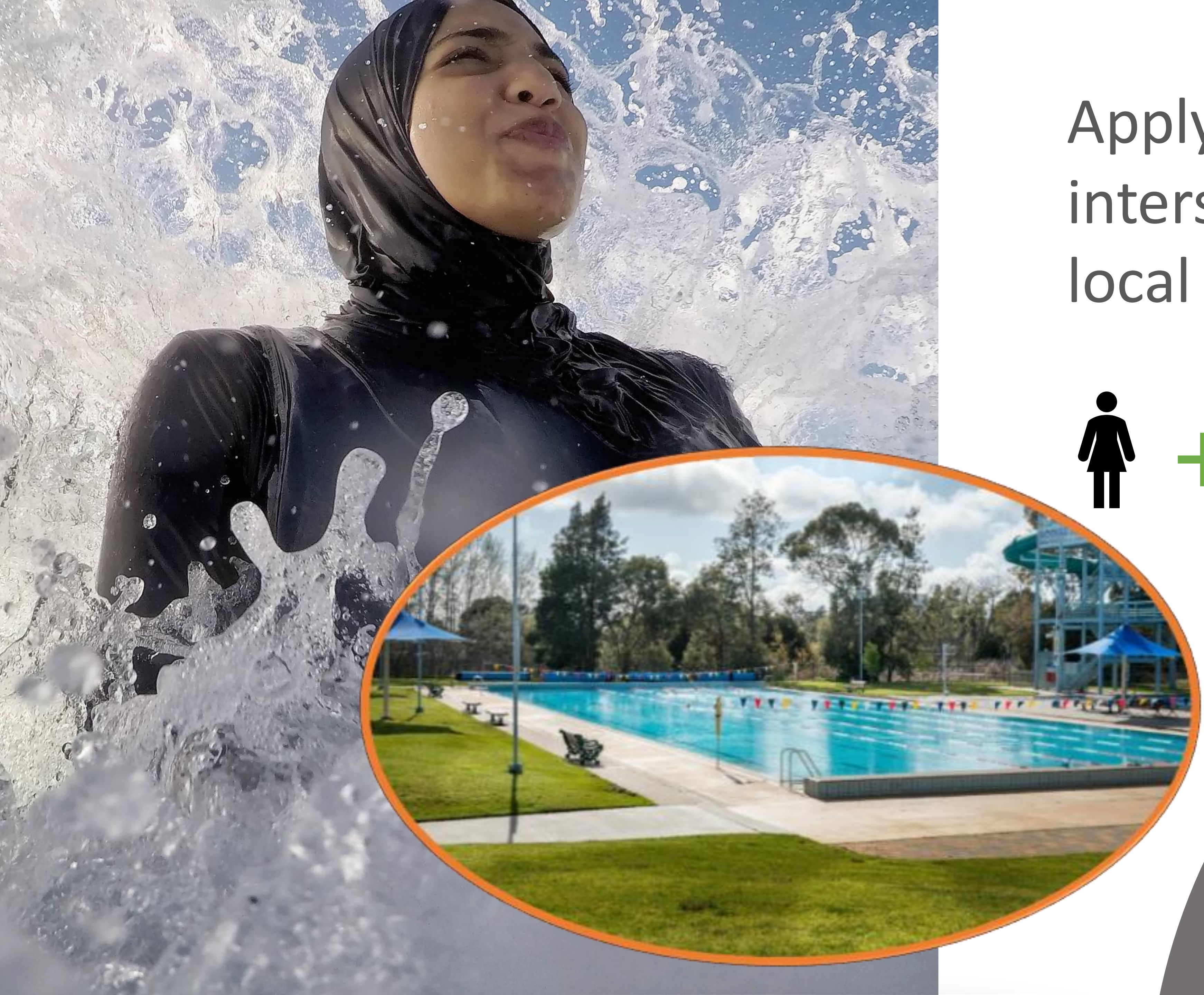

Applying an intersectional lens to local service delivery

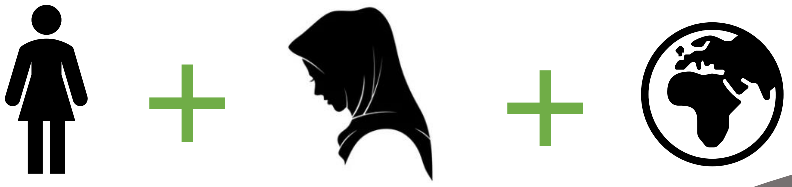

Applying an intersectional lens to local service delivery

+ +

REGIONAL VICTORIANS OF COLOUR

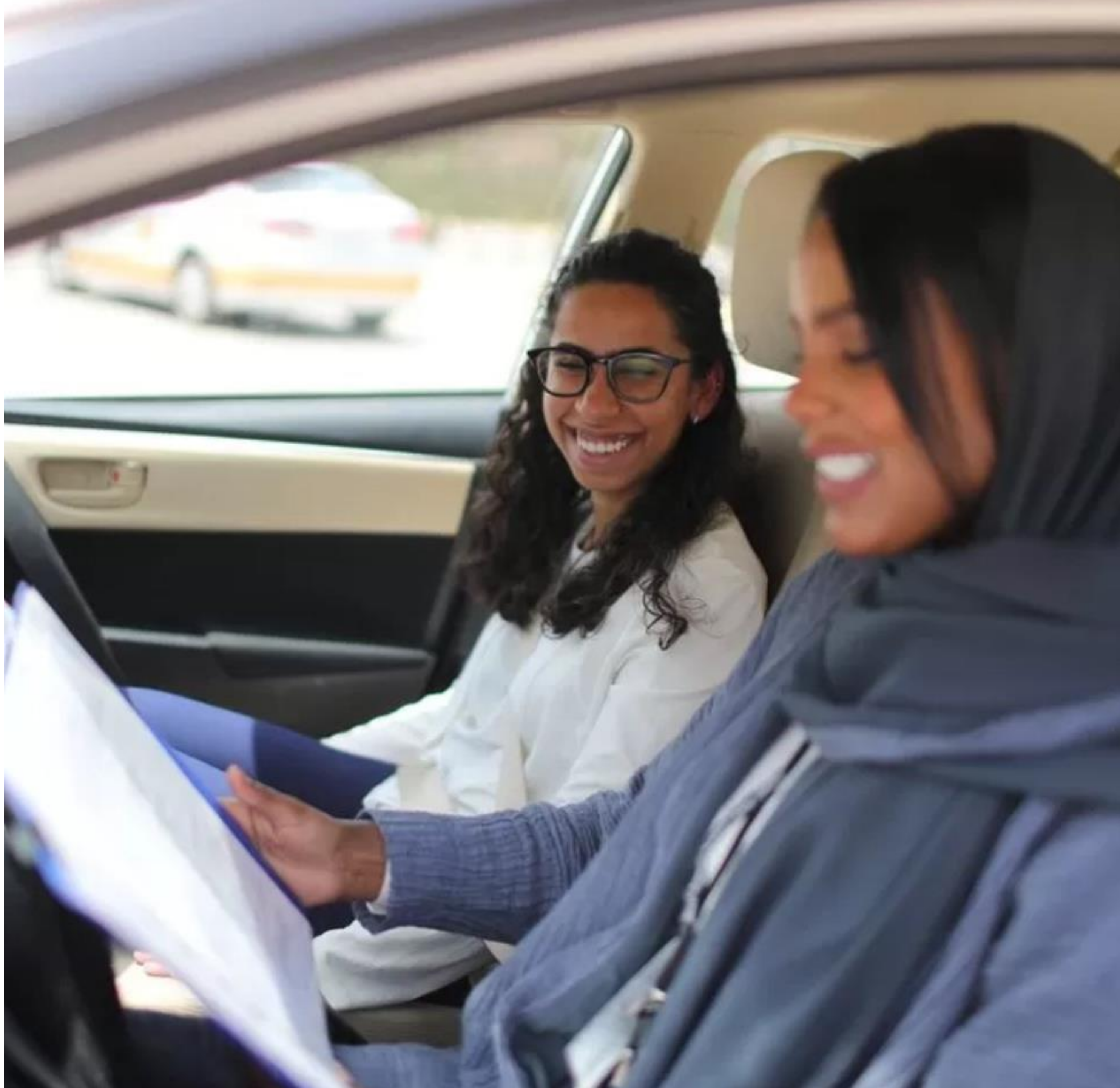

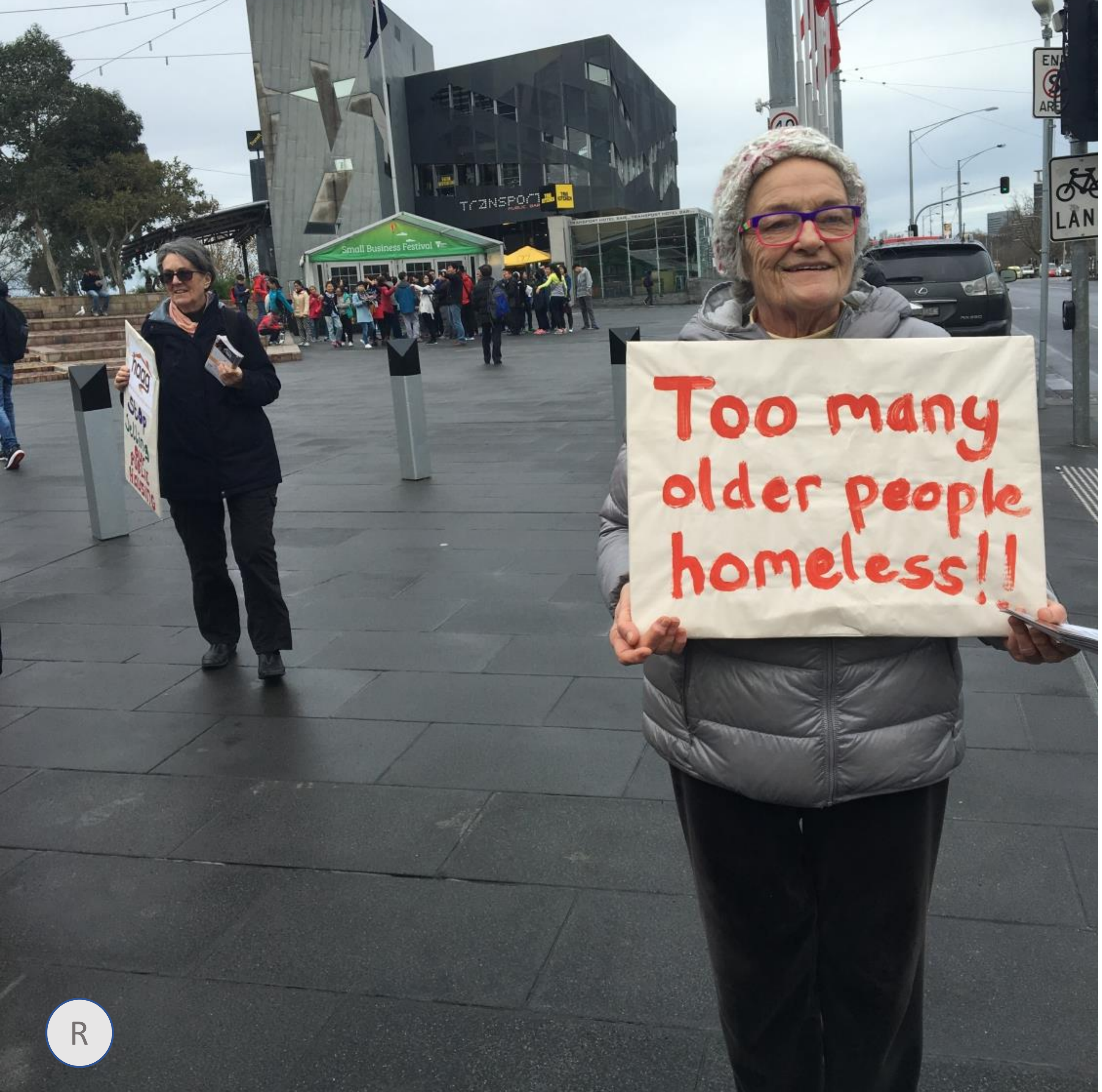

Applying an intersectional lens to local service delivery

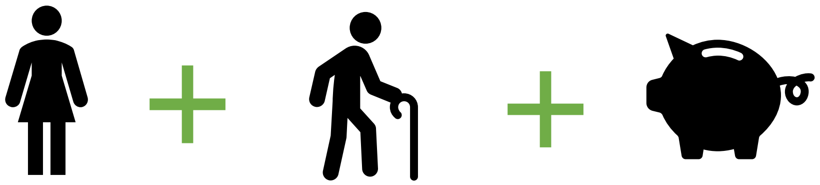

Women over 55 are the fastest growing group of people seeking assistance from homelessness services in Victoria.

Practice

Meeting recorded

Limited number of participants (3-5)

2min thinking time per Q. Answer Q's in chat prior to discussion.

Reference question number in comments. Q4:

'Like' and be active in comments

Open discussion welcome. Raising hand not required.

General

1.

What is the issue the policy is trying to address? Why is it needed?

Be as specific as you can.

2.

Who is impacted by the policy?

Are some more impacted than others?

People - Gender

3.

Do the different social roles and responsibilities people take on influence how they are impacted by the policy?

Consider gender norms, and how these might differ across multi-cultural or religious communities.

4. What are the different needs of different genders who are impacted by the policy?

What action can be taken to better meet these needs?

Women

Gender diverse/ non-binary

Men

People - Intersectionality

5.

What needs might there be for people who experience gender inequality alongside other forms of discrimination?

What action can be taken to address these needs?

Gender + Aboriginality | Age | Disability | Ethnicity | Sexuality | Religion | Socio-economic status

Place

6.

What impact does location have on the needs of diverse people impacted by the policy?

How can this be addressed?

Neighbourhoods

Areas

Regions

Experience

7. What experiences may prevent people from benefiting from the policy?

   How can this be addressed?

- Employment status
- Housing/ Homelessness
- Food security
- Migration/ refugee/ Seeking asylum
- Trauma/ Intergenerational trauma
- Past experiences of discrimination
- Family violence
- Chronic Illness
- Poverty/Low income
- Household composition (single parent, family with young children, living alone)

8.

What barriers and enablers might influence diverse participation in community engagement?

Consider literacy (English, digital and language of origin), time or financial barriers, venue accessibility, level of trust in government bodies, extent and diversity of reach.

9.

What will you focus on in this assessment to support a more equitable outcome for the community?

Ensure your focus addresses gender inequality to be compliant with the Gender Equality Act 2020.

Thank you!
See you in 4-6 weeks at the final workshop

CITY OF GREATER BENDIGO

EIA Final Workshop

<Insert policy, program or service name>

CITY OF GREATER BENDIGO

Acknowledgment of Country

I acknowledge that the City of Greater Bendigo is on Dja Dja Wurrung and Taungurung Country.

I would like to acknowledge and extend my appreciation for the Dja Dja Wurrung* People, the Traditional Owners of the land that we are standing on today.

Today, we pay our respects to leaders and Elders past, present and future for they hold the memories, the traditions, the culture and the hopes of all Dja Dja Wurrung* Peoples.

We express our gratitude in the sharing of this land, our sorrow for the personal, spiritual and cultural costs of that sharing and our hope that we may walk forward together in harmony and in the spirit of healing.

EIA Focus:

<Insert EIA focus>

Step 2:

What did the data, research and targeted consultation say?

Step 3:

Proposed options to improve the policy, program or service

Option #1:

Option #2:

Step 4: Proposed EIA Recommendation

- Meets the different needs of different genders (legislative requirement)
- Addresses gender inequality or promotes gender equality (legislative requirement)

<Insert EIA recommendation>

Q&A

EIA Champion Role Description

What is an Equity Impact Assessment (EIA)?
Equity Impact Assessments (EIAs), incorporating Gender Impact Assessments, consider how policies, programs and services affect different genders and diverse groups in different ways and recommend changes that will help create a more fair and inclusive community.

What is an EIA Champion?
EIA Champions are staff members who are trained to help other staff conduct EIAs. They ensure EIAs are conducted in a way that is legislatively compliant with the *Gender Equality Act 2020* and facilitate workshops to support staff explore ideas of equity and inclusion in relation to their policy, program or service. The City aims to have an EIA Champion appointed in every business unit by the end of 2023.

Why be an EIA Champion?

The EIA Champion role will benefit those who are:

- passionate about social and gender equity
- interested in increasing their visibility at the City
- interested in professional development and networking opportunities

EIA Champions will meaningfully engage with a diverse number of staff across varying units and directorates on a regular basis. They will gain unique insights into how different genders and diverse groups engage with the City's policies, programs and services and what barriers and enablers are at play in achieving a fair and inclusive community. EIA facilitation also supports Champions to develop their interpersonal, facilitation, presentation and time management skills.

EIA Champion qualities:

- open-minded and tolerant of a broad spectrum of beliefs, ideas and perspectives
- foster environments in which people feel safe and welcomed to share honestly
- are confident to facilitate discussion with groups of diverse staff and ensure all voices are heard
- able to keep others on track, complete projects and meet deadlines

Estimated workload
EIA Champions will be requested to facilitate 1 x EIA per quarter which will amount to the equivalent of 2 x working days per quarter. EIA Champions will also be asked to be a point of reference for staff in their unit who may have queries or seek advice about the EIA process.

Support for EIA Champions
EIA Champions will receive specialised training to learn EIA facilitation. They will also receive direct and ongoing support by the City's Gender Equity Officers. Staff will be supported by the City's EIA Guide and EIA Templates which contain all the necessary information and tools to conduct a successful EIA.

Appointing an EIA Champion
Please carefully consider who you wish to nominate as an EIA Champion within your unit, ensuring their interest, capacity and confidence to carry out the role. Once a staff member has accepted your nomination, please notify a Gender Equity Officer (ge@bendigo.vic.gov.au) of this appointment by **Friday 12 August**.

CITY OF GREATER BENDIGO

Equity Impact Assessment (EIA) Case Study

Name of initiative: Community Grants Policy Review

Directorate: Healthy Communities and Environments

Date: March 2021

Policy, program or service background

The City of Greater Bendigo's Community Granting Policy (2018) and Program provides an opportunity for incorporated not-for-profit groups and registered businesses to develop projects that complement areas identified as priorities in the Community Plan 2017-2021 and the City's vision of 'creating the world's most liveable community.'
The Policy is reviewed periodically to ensure the grants program remains relevant and meets community need.

EIA focus

What was identified as the focus for further research?

Is gender diversity represented in the organisations/groups who apply for grants and who have their funding approved, and what changes should be implemented to redress imbalances if they exist?

EIA findings *What did the data, research and targeted consultation say?*

The data collection undertaken revealed that 87.8% of the grants we award funding to, are gender neutral. The desktop research further revealed that gender neutral program and projects rather than benefitting all genders advantage men more than women, given that women are in a more disadvantaged position to begin with.

Researchers have recognised that most responses to our environment happen without our awareness with only 10 percent of discrimination being explained by the conscious mind. One measure to overcome unconscious bias is to involve a cross-section of decisionmakers: research shows that including a critical mass of underrepresented groups in the decision-making process reduces bias.

Australians Investing in Women have developed a Gender-wise Toolkit that has a suite of tools for grant makers to utilise to help them apply a gender-wise lens.

EIA recommendations *What changes were recommended to the policy, program or service in order to create more equitable outcomes for the community?*

To progressively develop and implement tools that will support greater Community Grants Program equality by:
- Improving capacity for data capture and analysis about who is applying for and who is benefitting from the grants
- Improving / diversifying the formats through which information about the Community Grants Program is shared and ways in which community grants can be applied for
- Strengthening grant seeking capabilities within our community
- Supporting people involved in grant allocation decision processes to develop greater gender-wise and intersectionality knowledge and skills.

Equity Impact Assessment (EIA) Case Study

Name of initiative: Graffiti Management Policy

Directorate: Presentation and Assets

Date: October 2021

Policy, program or service background

The purpose of the Graffiti Management Policy is to establish a coordinated approach to managing, preventing and removing graffiti within the Greater Bendigo municipality. In doing so the aim is to:

- address perceptions of public safety
- protect assets
- differentiate graffiti from public art
- address how we deal with graffiti on private property
- understand the City's responsibilities within the State Government Act
- embrace multiple forms of prevention and education around graffiti

EIA focus

What was identified as the focus for further research?

Investigate how the community experiences graffiti, and whether there is merit in re-shaping community perceptions around it.

EIA findings *What did the data, research and targeted consultation say?*

A narrow understanding of street art/graffiti does not consider the substantial research and experience around the world that testifies to the many contributions to city life that street art/graffiti can offer.

Expanding our sites of pedagogy to include public spaces and public art provides us with the opportunity to highlight not only how literacy permeates our everyday lives but also how community literacy—in this case, as street art in public spaces—represents a valuable public pedagogy that can help us communicate more effectively across difference.

EIA recommendations *What changes were recommended to the policy, program or service in order to create more equitable outcomes for the community?*

Vision: to change the tone of the policy to be one of inclusion, education encouragement and celebration.

1. Change definition of mural
2. Create a study/database of the graffiti being removed as an education tool
3. Create more detail around the education and engagement strategies
4. Add an additional responsible person

This GIA recommends that amendments are made to the draft Graffiti Management policy that increase emphasis on prevention, education and engagement elements of the policy, and by doing so, improve community perceptions of safety and promote diversity of expression. To achieve this, the recommendation encourages an additional responsible person be assigned to the policy from the public spaces activation space who will oversee the implementation of prevention, education and engagement initiatives outlined in the strategy.

The recommendation also encourages the City to improve its graffiti literacy by documenting graffiti in the removal process and analysing it to understand what community members are communicating and how/if these needs can be addressed as a form of prevention. Further, the recommendation encourages the definition of mural in the policy be updated to be consistent with industry standards.

Equity Impact Assessment (EIA) Case Study

Name of initiative: Healthy Facilities Policy

Directorate: Healthy Communities and Environments

Date: June 2022

Policy, program or service background

The purpose of this policy is to provide healthy, safe and sustainable food and drink options in all City of Greater Bendigo owned, managed or funded facilities and venues as part of the City's commitment to promoting and protecting the health and wellbeing of all Greater Bendigo residents. This policy also aims to ensure waste minimisation and other sustainable practices are fostered when promoting or selling food and drink items within City owned, managed or funded facilities and venues.

EIA focus

What was identified as the focus for further research?

The focus landed on at the end of the EIA preliminary workshop was how to support volunteers (who are primarily women) with support resources for: Healthy options that are low impact from a time perspective and healthy options that consider affordability

EIA findings *What did the data, research and targeted consultation say?*

General research from the Healthy Facilities Policy consultation indicates that our local sporting clubs are time poor, and will need examples of low cost, quick and easy food and drink options that meet the policy. Current research during the policy consultation period also indicated that sporting clubs have suffered throughout COVID and their priorities are not with the canteen/healthiness of food and drink offerings, but with maintaining and recruiting volunteers to operate.

Research shared from Sports focus also indicated that their most recent survey of clubs conducted across Regional Victoria was pre-pandemic (in 2019) where it was found that 58% of clubs had 40% or more women on their committees, however in terms of gendered roles, 75% of club presidents (leaders) were male whilst 63% of club secretaries (i.e. the club do-ers) were female. From this survey it was also reported that, pre-pandemic the number one rated challenge for club administrators was Lack of volunteers ; number two was recruiting and retaining committee members and number 3 was compliance/red tape . Anecdotally Sports Focus have also reported that volunteer decline has been further exacerbated by COVID-19. Early into the pandemic Sports focus also facilitated approximately 150 guided conversations with clubs across the Greater Bendigo region (predominantly the club secretaries) which did identify some of the gendered impacts of COVID-19. Several women reported they were struggling with home-schooling, working from home and running their club through what was an extremely difficult period of lockdowns and restrictions - with responsibility for covid planning tending to fall back onto the secretaries (majority women). Many indicating their desire to step away from their role with the club. Sports Focus is still receiving daily calls from clubs struggling to fill volunteer roles including in regard to filling canteen rosters now community sport has returned as we 'live with COVID-19'

EIA recommendations *What changes were recommended to the policy, program or service in order to create more equitable outcomes for the community?*

The adopted strategy includes food and drink options in the Healthy Facilities Policy support resources that are low in cost and low in time to prepare. By providing as practical options for club canteen managers/supervisors who are often women, the impact of policy implementation will be lessened.

Equity Impact Assessment (EIA) Case Study

Name of initiative: Kangaroo Flat Skate Park Development

Directorate: Presentation and Assets **Date:** October 2021

Policy, program or service background

The Kangaroo Flat initiative aims to provide for the growing residential demographic and address the lack of community services in the area. The initiative aims to offer a diverse range of active pursuits to complement the City s existing recreational infrastructure.
Skate parks are generally designed by skateboarders, for skateboarders. Kangaroo Flat recreation infrastructure will cater to all gender identifications, age groups and physical abilities.

EIA focus
What was identified as the focus for further research?

To explore ways to activate Bendigo skate parks that encourages higher participation from girls, women, people with different abilities and other under-represented demographics.

EIA findings *What did the data, research and targeted consultation say?*

- Data sourced from Geelong City Council suggests that boys and girls participate equally in wheeled, including mountain bikes, activities and competitions up to the age of approximately 13 years old.
- It is not until 13 years old that participation drops off dramatically for girls and remains unequal amongst adults, due to both social and structural gender stereotypes.
- No available data recording race or gender identification heterosexual, LGBTIQ+.
- Sports spaces are usually rigorously organised along gendered lines due to the broader gender binarization that characterises sports.

EIA recommendations *What changes were recommended to the policy, program or service in order to create more equitable outcomes for the community?*

Festival of Inclusion
Seasonal community engagement events will be held to focus on under-represented user groups. The events would be used as info sessions to showcase existing opportunities; generate valuable design options for recreation infrastructure; and provide opportunities for residents to engage with the greater community. The events will be held at existing activity nodes as a centre piece for the expression off people and place.
Expression Sessions
A Skate Club sporting program will be established for personal progression and having fun. The program will be broken into multiple levels incorporating an elevated degree of technical difficulty, covering the basics of street and transition skateboarding and scootering (the main sports identified in GIA research.)
Group lessons would be offered in numerous bands:
-Skate Club intermediate, ages 5 to 15
-Skate Club advanced, ages 5 to 17
-Go Girls girls only, beginner, ages 5 to 14
-Progression Session girls only, beginner, ages 14+
-First Push beginner / first time, ages 5 to 14
-Adult Skateboard Group Class beginner, 18 and over.

Equity Impact Assessment (EIA) Case Study

Name of initiative: Maiden Gully Site and Infrastructure Plan

Directorate: Healthy Communities and Environments

Date: September 2022

Policy, program or service background

To reimagine and plan the Maiden Gully Recreation Reserve as a future location that provides organised and unorganised sport and recreation options that caters for future community needs in inclusive and diverse ways.

EIA focus

What was identified as the focus for further research?

Explore ways in which diverse community members, who are often under-represented in engagement, can be encouraged to participate in the engagement process. Consider avenues such as LCMS, TGD Bendigo & Beyond, Youth Council and Playgroups.

EIA findings *What did the data, research and targeted consultation say?*

Maiden Gully ABS Quick Stats indicate the average (mean) income of Maiden Gully is higher than the Victorian average and over-represented by young families. It is one of the highest growth areas in Bendigo and does not currently have the recreation facilities to support the current or future population. While considering the average, it is important to also reflect on the intent of the EIA which focuses on the under-represented and vulnerable people within a population, considering the trends and range of incomes, abilities, backgrounds and gender.
As part of our commitment to gender and wider equity, in creating a space for health and wellbeing into the future, we need to hear the voices of all people who will use and potentially use the space.

EIA recommendations *What changes were recommended to the policy, program or service in order to create more equitable outcomes for the community?*

- Record a video for community consultation and EIA explanation
- Complete double the listening posts at different times of the day, including at a community event that is low or no cost
- Conduct focus groups with identified stakeholders women, gender diverse people and women or gender diverse people who experience intersectionality (socio-economic status, culture, young people, disability)

This approach creates equitable outcomes for the community by:
- Specifically speaking to those who are underrepresented and vulnerable
- Allowing for easily accessible material for the community to understand the information we are seeking 1-2 min video
- Multiple pathways for receiving project information visual, written, internet-based, through community connections, signage
- Multiple pathways to give feedback written, internet-based, verbal one-on-one in the community, verbal in targeted focus groups
